# Sex differences in bacterial meningitis and associations with socioeconomic indicators: a systematic review and meta-analysis with metaregression

**DOI:** 10.1136/bmjgh-2024-016802

**Published:** 2025-04-30

**Authors:** Fabian D Liechti, Cornelis N van Ettekoven, Matthijs C Brouwer, Merijn Bijlsma, Diederik van de Beek

**Affiliations:** 1 Department of General Internal Medicine, Inselspital, Bern University Hospital, University of Bern, Bern, Switzerland; 2 Department of Neurology, Amsterdam Neuroscience, Amsterdam UMC, University of Amsterdam, Amsterdam, The Netherlands; 3 Department of Neurology, HagaZiekenhuis, The Hague, The Netherlands; 4 Department of Pediatrics, Amsterdam Neuroscience, Amsterdam UMC, University of Amsterdam, Amsterdam, The Netherlands

**Keywords:** Meningitis, Global Health, Systematic review, Pneumococcal disease

## Abstract

**Introduction:**

We aimed to describe global sex-specific proportions and case fatality ratios of bacterial meningitis and to explore their associations with the Human Development Index (HDI) and Gender Inequality Index (GII).

**Methods:**

Google Scholar and MEDLINE (via PubMed.gov) were searched in January 2022 using the terms “bacterial meningitis” and “mortality”. Studies with a mean observation period after the year 1940 and reporting ≥10 patients with community-acquired bacterial meningitis and their survival status were included, irrespective of the participants’ age. Studies that selected participants by specific risk factors, reported specific pathogens only, or had >10% missing outcomes were disregarded. Data were extracted by one researcher and validated by a second researcher. The main outcomes, sex-specific proportions and case fatality ratios, were analysed using random-effects models. Associations with HDI and GII were explored using metaregression.

**Results:**

In this meta-analysis with metaregression, from 371 studies with 157 656 meningitis episodes, 217 (58%) reported the patients’ sex and 41 (11%) reported sex-specific outcomes. Proportion of males was 58% (95% CI 57%–59%, prediction interval (PI) 45%–71%). Case fatality ratios were slightly higher in females (male-to-female fatality ratio, 0.89, 95% CI 0.78 to 1.01, PI 0.53–1.49). The size of the male proportion was strongly associated with HDI (per index point, −0.64, 95% CI −0.88 to −0.40; R^2^ 16%; p<0.001) and GII (per index point, 0.61, 95% CI 0.39 to 0.83; R^2^ 19%; p<0.001). Sex-specific case fatality ratios were weakly associated with HDI (per index point, 0.53, 95% CI −0.19 to 1.25; R^2^ 2%; p=0.15) and GII (per index point, −0.58, 95% CI −1.55 to 0.39; R^2^ 7%; p=0.24).

**Conclusion:**

Based on worldwide reporting from the last 80 years, we show that indicators of human development and gender inequality are associated with sex-based disparities and case fatality ratios in bacterial meningitis.

**WHAT IS ALREADY KNOWN ON THIS TOPIC**

- Individual studies of bacterial meningitis have often reported unequal proportions of male and female patients, as in other infectious diseases. Furthermore, studies have suggested differences in disease severity and treatment response between sexes. However, sex-based differences in susceptibility or outcome have not been systematically investigated in bacterial meningitis.

**WHAT THIS STUDY ADDS**

- In this systematic review and meta-analysis, we found that most reported cases were male, but patients’ sex was not reported in nearly half of the studies. Sex-specific case fatality ratios were only reported in about 10% of meningitis episodes. Males had higher rates of meningitis and better outcomes than females. However, in countries with the highest income or lowest gender inequality, these disparities diminish, showing similar sex proportions and outcomes between males and females. Higher socioeconomic indicators were consistently linked to lower sex proportions and meningitis mortality.

**HOW THIS STUDY MIGHT AFFECT RESEARCH, PRACTICE OR POLICY**

- The limited sex-specific reporting underscores the need for greater attention to sex and gender in clinical trials and observational studies. Future studies should examine the underlying reasons for sex differences in reported incidence and outcomes of meningitis. Ensuring equal access to healthcare for all individuals, regardless of sex or gender, is crucial.

## Introduction

 Sex and gender have been recognised as important effect modifiers in infectious diseases, influencing susceptibility, clinical characteristics and outcomes.^1 2^ Studies in bacterial meningitis have frequently reported unequal proportions of males and females,^3^,^6^ but the magnitude of the differences reported is variable, and furthermore, we find much smaller differences between the sexes in our own prospective nationwide bacterial meningitis cohorts in the Netherlands and in other large European cohort studies.^12 7^,^9^ Additionally, some studies have suggested differences in illness severity and treatment response between sexes.^110^,^14^ Nonetheless, sex-specific differences in infection ratio or outcome have not been systematically investigated in bacterial meningitis. If such differences do exist, they could be attributed to biological differences (sex-based differences) as well as social, cultural or economic differences (gender-based differences) such as malnutrition, stress, risk behaviour, health-seeking behaviour, comorbidities or differences in quality of care. The objectives of this systematic review and meta-analysis are to describe the global male-to-female ratio of patients with community-acquired bacterial meningitis over time and to investigate sex-specific case fatality ratios (CFRs) worldwide. Subsequently, we explore possible explanations for sex and gender disparities in bacterial meningitis by using indicators of social development, that is, the United Nations Development Programme’s (UNDP) Human Development Index (HDI) and Gender Inequality Index (GII) and income inequality (Gini Index).^15^,^17^

## Methods

### Study selection

For this systematic review and meta-analysis, Google Scholar and MEDLINE (via PubMed.org, last search on 1 January 2022) were searched using the terms “bacterial meningitis” and “mortality” or “case fatality” (online supplemental table 1). Thereby, we made use of a previously published systematic review and meta-analysis on worldwide CFRs over an 80-year period.^18^ Studies were included when they reported on community-acquired bacterial meningitis with their mean study period after 1940 and described at least 10 patients with their survival status. Community-acquired bacterial meningitis was defined as a patient with clinical symptoms of bacterial meningitis, as defined by the authors of the studies. Studies were excluded when they recruited patients based on a specific risk factor (eg*,* immunosuppression and disease severity), when they included 10% or more non-community-acquired bacterial meningitis cases (eg*,* healthcare-associated cases, ventriculitis and tuberculous meningitis) or when 10% or more outcomes were missing.

Duplicates were removed using Rayyan and Endnote (V.21.0.1). One researcher (CNvE) screened titles and abstracts of the remaining 4138 articles and excluded 3162 articles. The remaining 976 articles were assessed in full text by one author (CNvE), and if deemed necessary by this researcher, discussed with a second researcher (FDL). In total, 373 articles were excluded (scope or study design, n=155; overlap, n=167; case reports, n=17; nosocomial meningitis, n=17; tuberculous meningitis, n=7; selection based on risk factor, n=10). Through cross-reference checking 27 additional articles were identified-. Studies describing only specific pathogens (n=259) were disregarded, leaving 371 publications in the analysis, which was confirmed by a second researcher (FDL). Studies needed to report on an individual level the sex of meningitis cases to be included in the analysis of sex proportions and sex-specific outcomes to be included in the analysis of sex-specific CFRs. If only overall sex proportions or sex-specific CFRs were indicated in a publication, studies were included when 90% or more of these cases were community-acquired bacterial meningitis. As the aim was to include studies from diverse settings and over an 80-year span, no formal risk-of-bias assessment was done other than checking for small-study effects and publication bias by visual inspection of funnel plots. Also, exploration of heterogeneity in subgroups and metaregression models was part of the research question.

### Data extraction

The relevant study variables (author, publication year, country, inclusion period and age group) were extracted according to a (non-registered) protocol by one researcher (CNvE) and validated by a second author (FDL). For studies reporting on more than one inclusion period or age group, the sample was split into study periods (k). The total number of patients with community-acquired bacterial meningitis, the number of male patients and female patients and the total and sex-specific CFR were recorded for each study period. No differentiation was made between sex and gender because most studies assessed did not. When specific numbers of patients, deaths or the patients’ sex were not indicated, they were calculated based on provided proportions, if available. Therefore, the number of total patients included did not always round up to the sum of total male and female patients included.

### Socioeconomic indicator data

To explore heterogeneity, we used three country-specific, globally available composite indices. Since 1990, the HDI and the GII have been reported yearly in the UNDP Human Development Report (HDR).^15 16 19^ The HDI is based on three dimensions: life expectancy at birth, expected years of schooling and mean years of schooling and gross national income per capita (range 0–1; values 0.8 and above indicate very high human development).^16^ The GII is based on dimensions of reproductive health, empowerment and the labour market (range 0, indicating equality, to 1, indicating poorest gender equality).^16^ Yearly index values since 1990 were extracted from the HDR 2023–2024.^15 16^ Additionally, we extracted HDI values for 1980 from the HDR 2014 and values for 1950 from Crafts.^20 21^ The annual HDI values for the period 1940–1965 were imputed with those from 1950, the values for the period 1966–1985 were imputed with those from 1980, and the values for the period 1986–1990 were imputed with those from 1990 (online supplemental figure 1). For the GII, analysis was restricted to studies with their mean observation period of 1990 or later (online supplemental figure 2). When individual country data were not available, and for multinational studies, we used instead the values for the respective UNDP region, for example, Arab states, or if not available, values for countries with similar levels of income, for example, very high income, because this allowed the analysis of changes over time and should not lead to false-positive associations.

The Gini Index measures income distribution among individuals or households (range 0, indicating perfect equality, to 100, maximal inequality) and shows—in contrast to HDI and GII—little correlation with time.^17 22^ Yearly index values since 1960 were extracted from the World Bank, Poverty and Inequality Platform with imputing missing values by carrying the last observation forward (online supplemental figure 3).^17^

### Statistical analysis

To describe the male proportion and male-to-female CFR ratio of meningitis patients, random-effects meta-analysis models were built with a continuity correction of 0.5 in studies with a zero cell count, logit transformation and the restricted maximum likelihood (REML) method.^23^ Countries were categorised to World Bank regions for subgroup analysis (online supplemental figure 4), with three multinational studies attributed to the most appropriate region.^24^,^26^ If less than five studies were available, pooled effect estimates were omitted. Additional analyses were performed for the three age groups (neonates, younger than 2 months (regardless of gestational age at birth); children, 2 months to 16 years and adults, older than 16 years). To describe the global male proportion over time, a meta-regression model with the mean observation period as a predictor was constructed.

To explore the associations of sex differences in infection ratio and outcome, metaregression models were built: first, the association of the total CFR with HDI, GII and Gini Index was assessed. Then, we separately evaluated associations of both main outcomes (male proportion, male-to-female CFR ratio) with the socioeconomic indicators, HDI and GII. For metaregression models, we used the REML method and Wald-type CI. The results are presented as effect estimates with 95% CIs and test of moderator P values (based on permutation testing to assess true statistical significance). To assess between-study heterogeneity, we indicate 95% prediction intervals (PI) and the amount of heterogeneity accounted for (R^2^).^27^,^29^ For multiple metaregression models, we did not consider correlating predictors and used the maximum likelihood method to allow model comparison using likelihood ratio tests (LRT, 'anova' function in R).^27^ Sensitivity analyses included restriction to studies with their mean observation period after 2000. All analyses were performed using R Statistical Software (V.4.2.1; R Core Team 2021; online supplemental table 2).

Data collection started on 5 September 2018, with the last systematic literature search on 1 January 2022 and the last data collection, for example, socioeconomic indicators, on 31 January 2024. Data were analysed between March 2024 and July 2024. Reporting follows the Preferred Reporting Items for Systematic reviews and Meta-Analyses and Sex and Gender Equity in Research guidelines.^30 31^ The funder of the study had no role in study design, data collection, data analysis, data interpretation or writing of the report.

## Results

Of the 4138 publications identified, we included 371 original studies from 108 countries, reporting on 157 656 meningitis episodes with 23 728 deaths between 1 January 1935 and 31 December 2019 (mean observation period 1940–2019; flowchart in online supplemental figure 5); list of publications in online supplemental table 3).^612 13 24^,^26 32^ The studies reported on 427 study periods (k) with a median length of observation period of 1826 (IQR 730–3651) days (before 1961, k=32, 4509 episodes; 1961–1970, k=26, 4392 episodes; 1971–1980, k=47, 27 010 episodes; 1981–1990, k=91, 20 073 episodes; 1991–2000, k=107, 27 732 episodes; 2001–2010, k=81, 58 684 episodes; after 2010, k=43, 15 256 episodes) reporting on a median of 100 (IQR 50–259) episodes. Of these, 79 867 episodes (51%) were reported in 201 study periods from very high- or high-income countries (East Asia and Pacific, k=62, 8030 episodes; Europe and Central Asia, k=115, 31 922 episodes; Latin America and Caribbean, k=25, 13 430 episodes; Middle East and North Africa, k=40, 7125 episodes; North America, k=65, 45 934 episodes; South Asia, k=25, 3484 episodes; Sub-Saharan Africa, k=95, 47 731 episodes; online supplemental table 4). The male-to-female ratio of meningitis patients was reported in 217 of 371 (58%) studies (212 of 427 (50%) study periods; online supplemental table 5).^612 13 24 35 40 45 47 49 53 54 58 59 62^,^65 67^

### Sex-based differences in meningitis infection ratio and trends over time

At the individual patient level, of the 157 656 episodes, sex was reported for 79 820 episodes (51%), although some studies did not report sex for all study periods or subgroups differentially. In the random-effects model, 58% (95% CI 57% to 59%, PI 45%–71%) of meningitis patients reported were male, with the highest proportion of males found in South Asia (71%, 95% CI 64% to 76%, PI 43%–88%), while the smallest proportion of males was found in North America (54%, 95% CI 52% to 56%, PI 48%–60%) and Europe and Central Asia (56%, 95% CI 57% to 59%, PI 50%–63%; online supplemental figure 6). No funnel plot asymmetries were found (online supplemental figure 7). In all age groups, neonates (57%, 95% CI 54% to 60%, PI %41%–72%), children (59%, 95% CI 58% to 61%, PI 48%–70%) and adults (57%, 95% CI 54% to 60%, 40%–72%), the infection ratio was higher in males compared with females.

When considering studies with their mean observation period after 2000 only, the proportion of males was 58% (95% CI 56% to 61%, PI 42%–73%). After 2000, regional variations were still large with 76% (95% CI 63% to 85%, PI 29%–96%) males in South Asia and 64% (95% CI 59% to 68%, PI 50%–75%) males in Middle East and North Africa, compared with 55% (95% CI 53% to 57%, PI 53%–57%) males in Europe and Central Asia (online supplemental figure 8). Over time, male proportions did not change significantly using the mean observation period as a predictor in a metaregression model (per year, −0.0007, 95% CI −0.004 to 0.002; p=0.65; k=212; online supplemental figure 9).

### Sex-based differences in meningitis outcomes

CFRs were reported separately for males and females in 41 of 371 (11%) studies (43 of 427 (10%) study periods), corresponding to 15 431 of 157 656 episodes (9.8%; online supplemental table 6).^1213 35 47 53 59 68^,^72 76 79 80 90 111 129 130 133 151 158 163 173 222 230 232 251^

Considering some studies did not report male-to-female ratios on an individual level or not for all subgroups, the outcome was reported for 8957 males and 5945 females (male-to-female ratio 1.5:1). Overall, CFR was slightly higher in females (22%, 95% CI 17% to 28%; PI 4%–64%; online supplemental figure 10) compared with male patients (19%, 95% CI 15% to 25%; PI 3%–65%; online supplemental figure 11). No significant disparities between CFRs in male and female patients were observed across regions, with the CFR being slightly higher in females (male-to-female CFR ratio, 0.89, 95% CI 0.78 to 1.01, PI 0.53–1.49, (online supplemental figure 12). No funnel plot asymmetries were found (online supplemental figure 13).

A lower male-to-female CFR ratio was observed in children (0.80, 95% CI 0.68 to 0.94, PI 0.67–0.96; online supplemental figure 14), indicating CFR was higher in girls (14%, 95% CI 9% to 21%, PI 2%–51%) compared with boys (12%, 95% CI 7% to 19%, PI 2%–51%). Of note, the male-to-female CFR ratio and sex-specific CFR were calculated separately from individual cases reported with random-effects models and can thus not be directly converted. In neonates, the male-to-female CFR ratio was 0.99 (95% CI 0.70 to 1.40, PI 0.41–2.38) with a CFR of 41% (95% CI 25% to 60%, PI 7%–87%) in girls compared with 40% (95% CI 19% to 64%, PI 2%–96%) in boys. In adults, the male-to-female CFR ratio was 1.02 (95% CI 0.76 to 1.36, PI 0.54–1.93) with a CFR of 19% (95% CI 12% to 27%, PI 5%–50%) in women compared with 20% (95% CI 17% to 23%, PI 15%–25%) in men.

### Meningitis outcomes and socioeconomic indicators

The overall CFR was 18% (95% CI 16% to 19%, PI 3%–57%), according to the random effects model (online supplemental figure 15). When including study periods after 2000 only, the CFR was 16% (95% CI 14% to 18%, PI 3%–50%) with the highest CFR in Sub-Saharan Africa (26%, 95% CI 22% to 32%, PI 7%–63%) and the lowest CFR in North America (7%, 95% CI 5% to 11%, PI 2%–27%; online supplemental figure 16).

**Figure 1:**
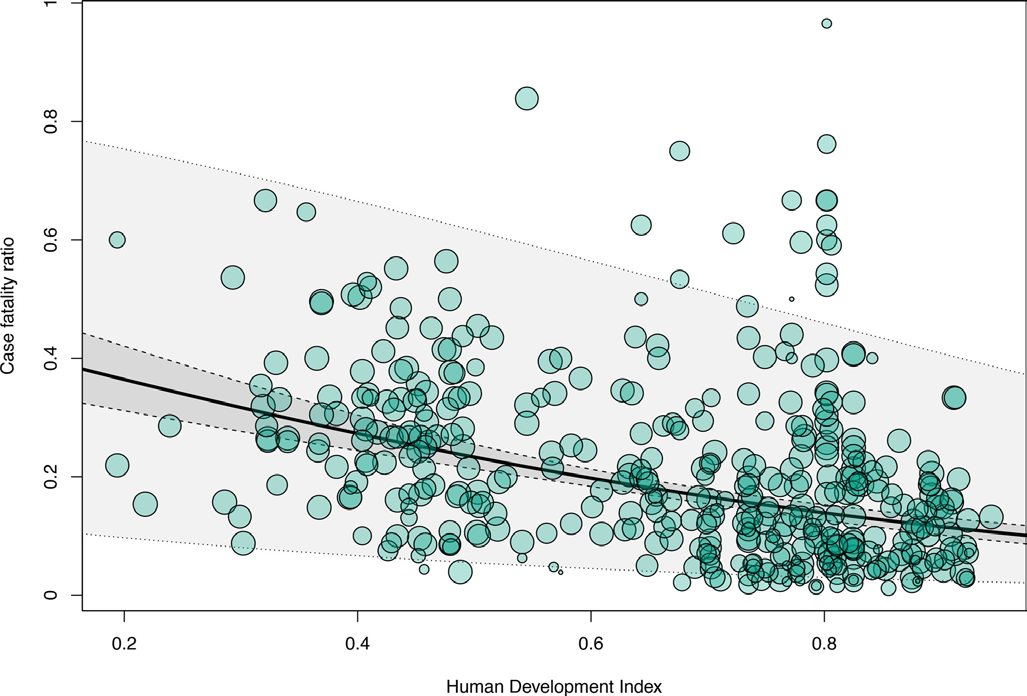
Case fatality ratios and human development. Metaregression of bacterial meningitis case fatality ratios using the Human Development Index as a predictor variable (p<0.001; I^2^=97%; R^2^=18%). (dashed lines, 95% CI; dotted lines, prediction interval).

Metaregression was first performed on the CFR from bacterial meningitis with HDI, GII or Gini Index as predictor variable and then—to explore heterogeneity—in age groups. CFR was lower in studies from countries with highest HDI values, representing very high-income countries (per HDI point, −2.1, 95% CI −2.6 to −1.6; R^2^ 18%; p<0.001), based on 424 study periods since 1940 (figure 1). In subgroup analyses according to age groups, this observation was confirmed in adults (per HDI point, −2.5, 95% CI −3.3 to −1.8; R^2^ 43%; k=78; p<0.001), children (per HDI point, −3.5, 95% CI −4.3 to −2.7; R^2^ 44%; k=132; p<0.001) and neonates (per HDI point, −3.5, 95% CI −3.8 to −0.9; R^2^ 13%; k=82; p=0.0014; online supplemental figure 17A–C).

CFR was higher in studies from countries with higher GII values, representing countries with higher gender inequality (per GII point, 1.5, 95% CI 1.1 to 2.0; R^2^ 17%; p<0.001), based on 236 study periods since 1990 (online supplemental figure 18). In subgroup analyses according to age groups, this observation was confirmed in adults (per GII point, 2.2, 95% CI 1.5 to 2.8; R^2^ 47%; k=61; p<0.001), children (per GII point, 2.7, 95% CI 1.6 to 3.8; R^2^ 27%; k=69; p<0.001) and neonates (per GII point, 1.9, 95% CI 0.4 to 3.3; R^2^ 16%; k=37; p=0.0012; online supplemental figure 19A–C).

CFRs were higher in countries with higher income disparities (per Gini Index, 0.037, 95% CI 0.027 to 0.048; R^2^ 14%; p<0.001), based on 353 study periods since 1960 (online supplemental figure 20). Again, the associations were consistent in the different age groups of adults (per Gini Index, 0.040, 95% CI 0.021 to 0.058; R^2^ 23%; p<0.001; k=70), children (per Gini Index, 0.046, 95% CI 0.026 to 0.065; R^2^ 18%; p<0.001; k=110) and neonates (per Gini Index, 0.027, 95% CI −0.002 to 0.057; R^2^ 4%; p=0.071; k=69; online supplemental figure 21A–C).

To further explore associations between CFR and socioeconomic indicators, we then used multiple metaregression models. For evaluating the association between CFR and HDI, adding the mean observation period (per HDI point, −2.2, 95% CI −2.7 to −1.8; per year, −0.02, 95% CI −0.02 to −0.01; R^2^ 26%; LRT p<0.001) improved the metaregression model only slightly. Additionally, adding the age group further improved the model (R^2^ 48%; p<0.001). For evaluating the association between CFR and the Gini Index, adding the mean observation period (per Gini Index point, 0.04, 95% CI 0.03 to 0.05; per year, −0.01, 95% CI −0.02 to −0.005; R^2^ 17%; LRT p<0.001) improved the metaregression model only slightly. Additionally, adding the age group further improved the model (R^2^ 28%; LRT p<0.001). Finally, the observations were also confirmed in the sensitivity analysis including studies with their mean observation period after 2000 only (online supplemental figures 22 and 23).

### Associations of sex-based differences in infection ratio and outcomes with socioeconomic indicators

**Figure 2:**
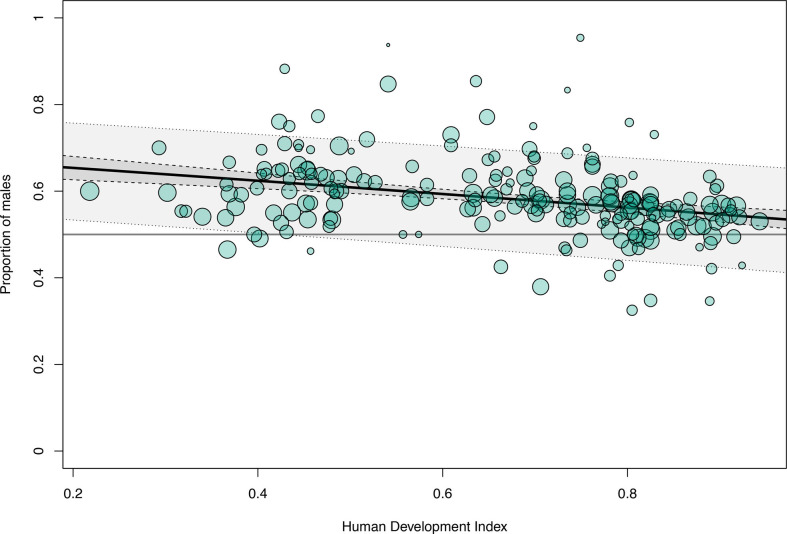
Sex proportions and human development. Metaregression of the proportion of male cases of bacterial meningitis using the Human Development Index as a predictor variable (p<0.001; I^2^=82%; R^2^=16%) (dashed lines, 95% CI; dotted lines, prediction interval).

**Figure 3:**
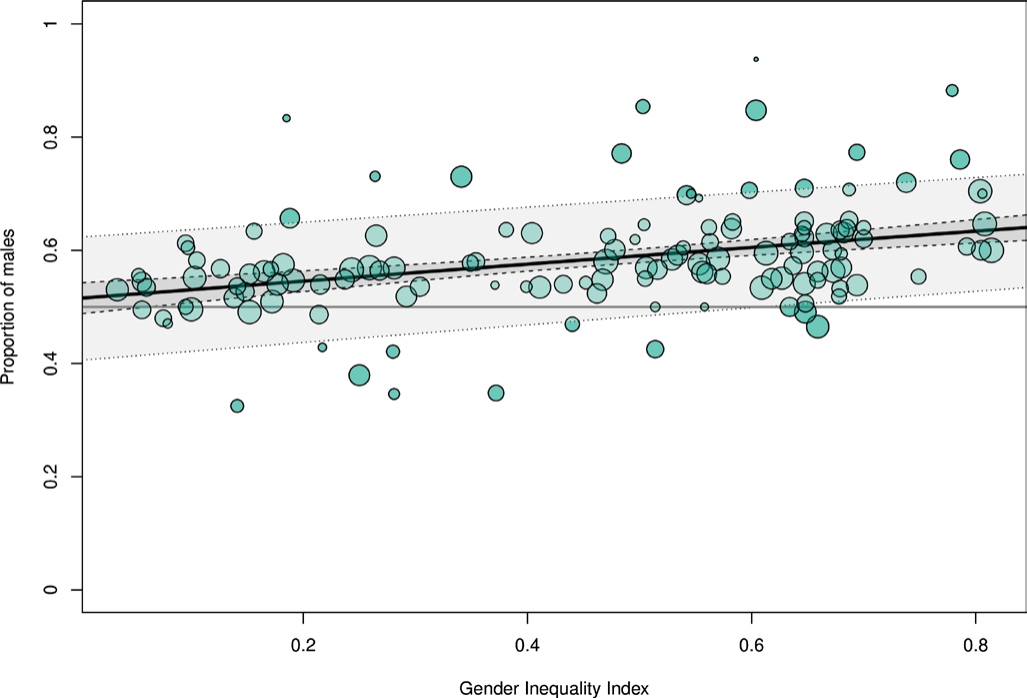
Sex proportions and gender inequality. Metaregression of the proportion of male cases of bacterial meningitis using the Gender Inequality Index as predictor variable (p<0.001; I^2^=75%; R^2^=19%), based on studies with a mean observation period of 1990 or later (dashed lines, 95% CI; dotted lines, prediction interval).

To explore the underlying mechanisms of the sex- or gender-based differences in infection ratio and outcome, metaregression models were built using socioeconomic indicators (HDI and GII) as predictors. In highly developed countries with a HDI close to 1, the male-to-female ratio of meningitis cases approached parity, whereas in countries with lower human development, higher proportions of meningitis cases were reported in males (per HDI point, −0.64, 95% CI −0.88 to −0.40; R^2^ 16%; p<0.001; k=211; figure 2). The male-to-female ratio approached parity when GII (available since 1990) was used as a predictor. In countries with higher gender inequality, more male patients were reported to have meningitis (per GII point, 0.61, 95% CI 0.39 to 0.83; R^2^ 19%; k=134; p<0.001; figure 3).

**Figure 4:**
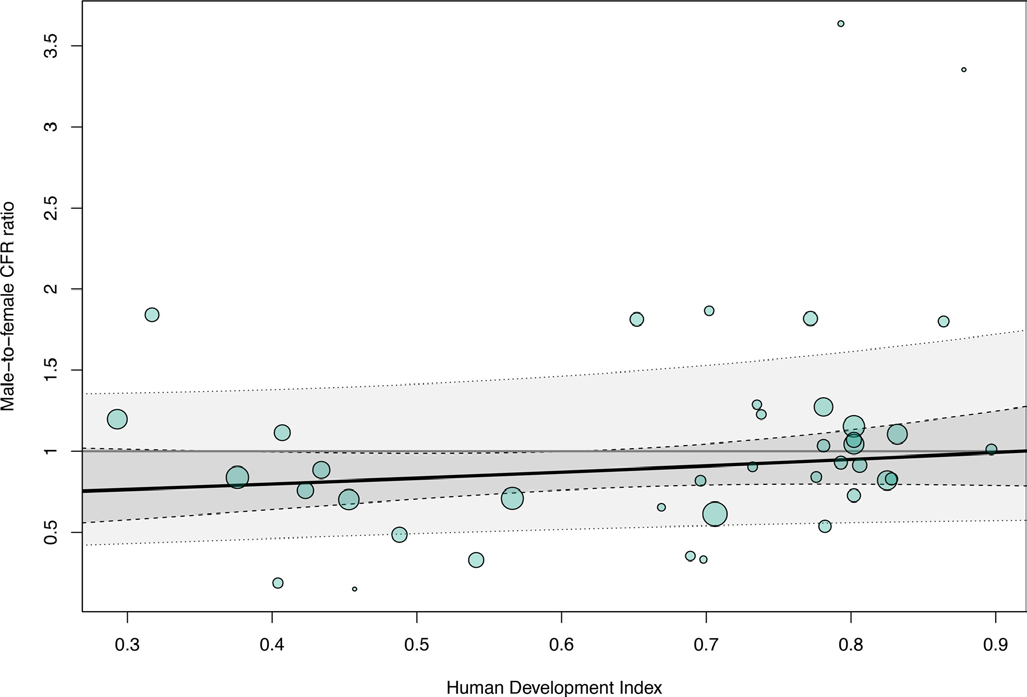
Sex-specific case fatality. Metaregression of the male-to-female case fatality ratio (CFR) ratio in bacterial meningitis using the Human Development Index as a predictor variable (p=0.26; I^2^ 57%; R^2^ 0%) (dashed lines, 95% CI; dotted lines, prediction interval).

In countries with a lower HDI value, female patients tended to have a higher CFR than male patients; conversely, in countries with a higher HDI, the male-to-female CFR ratio was close to 1 (per HDI point, 0.58, 95% CI −0.36 to 1.52; R^2^ 0%; k=41; p=0.26; figure 4). The effect was comparable when using GII as predictor, with lower gender inequality corresponding to lower differences in CFRs, although the evidence was weaker due to the smaller number of studies available for comparison since 1990 (per GII, −0.11, 95% CI −1.50 to 1.28; R^2^ 7%; k=19; p=0.88; online supplemental figure 22) and HDI and GII were correlated (Pearson correlation coefficient r=0.88) despite using different parameters.

## Discussion

Our study showed that human development, gender inequality and income disparities are associated with sex-based disparities and CFRs in community-acquired bacterial meningitis. The unadjusted analysis revealed a higher prevalence of bacterial meningitis among males, which disappeared in countries with higher human development and lower gender inequality. Higher levels of human development were strongly associated with lower CFRs, and larger income disparities were associated with higher CFRs. In countries with higher human development or lower gender inequality, CFRs were lower. These findings suggest that gender roles and the interaction between sex and gender could contribute to the discrepancies in infection ratio or outcome between males and females, although gender inequality is strongly associated with economic factors and can thus not be interpreted on its own.

Most reported cases were male, but patients’ sex was not reported in nearly half of the studies. Sex-specific CFRs were only reported in about 10% of meningitis episodes. The under-reporting of sex- and gender-specific results and the neglect of sex-based differences is well-documented.^31 398 399^ In less developed countries, where women’s access to healthcare may be more limited due to sociocultural, financial or structural barriers, differences in health-seeking behaviour, such as delays in seeking care, may partly explain the lower proportion of females admitted to hospitals and included in studies.^400^,^402^ Gender differences might also affect neonates and children, with girls potentially receiving less care than boys.^402 403^ This includes the notion that in many low-income countries, girls are less likely to attend school and may therefore be at a lower risk of contracting meningitis.^404^ Lower enrolment of female patients in studies, for example, due to different clinical presentation, health seeking behaviour or diagnostic testing strategies, may have influenced our results, as may different sex ratios between different countries and age groups, but we would expect these effects to be smaller than the effects we observed for meningitis incidence.

Differences in smoking rates may also have contributed to higher male susceptibility to bacterial meningitis. According to the WHO global report on trends in prevalence of tobacco, more men are smokers, although in high-income countries the proportions of men and women smoking are more balanced.^405^ Smoking increases the risk for both pneumococcal and meningococcal disease—the two most important pathogens of community-acquired meningitis.^406^,^408^ However, sex-based differences in outcomes were observed in children and neonates, suggesting that biological differences also play a role. Therefore, the biological risk for infections in males may indeed be higher, as reported earlier for neonates, while these sex-based differences may be less important in environments with better availability of healthcare and preventive measures regardless of the sex.^403^ As the GII is highly correlated with economic factors, it is difficult to assess gender inequality on its own. The socioeconomic factors used in the metaregression analysis on male-to-female CFR ratios explain only a small part of the heterogeneity compared with the subgroup analysis by age group or the other metaregression analyses. But the observation that sex discrepancies in CFR and incidence come close to disappearing in wealthier nations leads to the conclusion that the discrepancies observed in other populations are not primarily attributable to intrinsic, biological factors.

Better access to healthcare for all people in countries with high HDI and low Gini Index could explain the association of total CFRs with these two indices and the higher sex-specific discrepancies in CFRs in countries with lower development and more gender inequality. The association between high HDI values and low CFRs might be influenced by general developments in healthcare, such as advanced treatment options such as intensive care. However, among high-income countries, economic inequality as measured by the Gini Index varies;, for example, it was relatively low in the USA in recent years.^409^ Thus, the Gini Index is far less affected by technological or scientific improvements over time and decreased in several regions; still, the Gini Index was associated with CFRs in the current analysis.^22^ Despite possible biological differences, gender-based differences in treatment are a plausible explanation for discrepancies in CFRs between males and females.^2 10 14^ For example, one study showed that women were less likely to be admitted to an intensive care unit and to receive mechanical ventilation than men.^10^ The underlying mechanisms leading to such observations remain to be elucidated.

Our study has some limitations. First, this was a secondary analysis of previously reviewed data without quality assessment of included studies.^18^ Only reports including at least 10 patients were included, aiming for diverse settings, and no publication or small-study biases were found in funnel plots.^410^ Differential under-reporting cannot be excluded, especially since some analyses had a small number of studies. Under-reporting and delayed or forgone healthcare access can worsen outcomes, especially because bacterial meningitis is acute and fatal if untreated.^411^ Misclassification is also possible, for example, by not performing lumbar punctures, and diagnostic testing may be lower in females.^402^ However, misclassification bias would reduce differences in resource scarce settings. Confounding by other variables is likely, but the consistency of associations in the main analyses and in the sensitivity analyses, including different age groups, supports the reliability of results. Sex disparities consistently disappear when modelling settings approaching lowest gender inequality or highest human development. Using HDI, GII and Gini Index values required certain assumptions, e.g., using the last observation carried forward method for data imputation and the use of the original version for HDI values prior to 1990. While these composite measures have been criticised,^19 22 412^ they are available for almost all countries and over several decades, allowing for comparisons that other measures could not provide. Also, the three indices incorporate distinct parameters that are not directly correlated with healthcare measures, which could have caused false associations.

In conclusion, our study reveals that males are more likely to be infected with meningitis and have better outcomes than females. However, in countries with the highest income or lowest gender inequality, these disparities diminish, with similar infection ratios and outcomes between males and females. Lower socioeconomic indicators were consistently linked to more infections and higher meningitis mortality. The limited sex-specific reporting underscores the need for greater attention to sex and gender in clinical trials and observational studies. Ensuring equal access to healthcare for all individuals, regardless of sex or gender, is crucial.

## Supplementary material

10.1136/bmjgh-2024-016802 online supplemental file 1

## Data Availability

Data are available in a public, open access repository. All data relevant to the study are included in the article or uploaded as supplementary information.
