# Supplementary material for: Sex differences in bacterial meningitis and associations with socioeconomic indicators: a systematic review and meta-analysis with metaregression
Source: BMJ Glob Health. 2025 Apr 30;10(4):e016802. doi: 10.1136/bmjgh-2024-016802 (PMC12049963; doi:10.1136/bmjgh-2024-016802)
Supplement: online supplemental file 1 [file bmjgh-10-4-s001.pdf]

SUPPLEMENT

SUPPLEMENTARY TABLES 3
Supplementary Table 1. Search strategies used. 3
Supplementary Table 2. R commands used in the meta-analysis and meta-regression. 4
Supplementary Table 3. Characteristics of studies included. 5
Supplementary Table 4. Characteristics of studies reporting sex proportions among patients with bacterial meningitis. 18
Supplementary Table 5. Characteristics of studies reporting sex-specific case fatality ratios (CFRs) 26
Supplementary Table 6. Study periods included in the meta-analyses by mean observation period interval and World Bank region. 29

SUPPLEMENTARY FIGURES 28
Supplementary Figure 1. Imputed Human Development Index (HDI) values per country (colours) and year. . 31
Supplementary Figure 2. Imputed Gender Inequality Index (GII) values per country (colours) and year. 32
Supplementary Figure 3. Imputed Gini Index values per country (colours) and year. 33
Supplementary Figure 4. Classification of regions according to World Bank. 34
Supplementary Figure 5. Flow-chart of the study selection process. 35
Supplementary Figure 6. Proportions of males in bacterial meningitis patients stratified by World Bank regions (Forest plots with individual studies suppressed) indicating the estimates of the subgroups and the overall pooled estimate. 36
Supplementary Figure 7. Funnel plot studies indicating male proportions in bacterial meningitis patients. 37
Supplementary Figure 8. Proportions of males in bacterial meningitis patients including mean observation periods after 2000 only (sensitivity analysis) stratified by World Bank regions (Forest plots with individual studies suppressed) indicating the estimates of the subgroups and the overall pooled estimate. 38
Supplementary Figure 9. Proportions of males in bacterial meningitis patients using a meta–regression model with the mean observation period as predictor variable (P = 0.65). 39
Supplementary Figure 10. Forest plot illustrating case fatality ratios (CFR) in female patients with World Bank regions as subgroups. 40
Supplementary Figure 11. Forest plot illustrating case fatality ratios (CFR) in male patients with World Bank regions as subgroups. 41
Supplementary Figure 12. Forest plot illustrating case fatality ratios (CFR) male-to-female ratio with World Bank regions as subgroups. 42
Supplementary Figure 13. Funnel plot of studies indicating sex-specific case fatality ratios. 43
Supplementary Figure 14. Forest plot illustrating male-to-female case fatality ratio (CFR) ratios with age groups as subgroups. 44
Supplementary Figure 15. Meta-analysis of case fatality ratios (CFR) with regions as subgroups (all studies with mean observation period after 1940). 45
Supplementary Figure 16. Meta-analysis of case fatality ratios (CFR) with regions as subgroups (all studies with mean observation period after 2000 only. 46
Supplementary Figure 17. Case fatality ratio in bacterial meningitis with the Human Development Index as predictor, stratified by the age group (**A**, adults, ≥ 16 years old; **B**, children 2 months to 16 years old; **C**, neonates, < 2 months old). 47
Supplementary Figure 18. Case fatality ratio in bacterial meningitis with Gender Inequality Index (GII) as predictor. Studies with a mean observation period 1990 or later were included. 50
Supplementary Figure 19. Case fatality ratio in bacterial meningitis with Gender Inequality Index (GII) as predictor, stratified by the age group (**A**, adults, ≥ 16 years old; **B**, children 2 months to 16 years old; **C**, neonates, < 2 months old). Studies with a mean observation period 1990 or later were included. 51
Supplementary Figure 20. Case fatality ratio in bacterial meningitis with the Gini Index as predictor. Studies with a mean observation period 1960 or later were included. 54

Supplementary Figure 21. Case fatality ratio in bacterial meningitis with the Gini Index as predictor, stratified by the age group (**A**, adults, ≥ 16 years old; **B**, children 2 months to 16 years old; **C**, neonates, < 2 months old). 55
Supplementary Figure 22. Case fatality ratio in bacterial meningitis with the Human Development Index as predictor (including mean study periods after 2000, sensitivity analysis). 58
Supplementary Figure 23. Case fatality ratio in bacterial meningitis with the Gini Index as predictor (including mean study periods after 2000, sensitivity analysis). 59
Supplementary Figure 24. Male-to-female case fatality ratio (CFR) ratio in bacterial meningitis with Gender Inequality Index as predictor (mean study period 1990 or later). 60

REFERENCES 58

Supplementary Tables

Supplementary Table 1. Search strategies used.

Database	Search term
**Medline (PubMed), since inception, searched on January 1, 2022**	(("meningitis, bacterial"[MeSH Terms] OR ("meningitis"[All Fields] AND "bacterial"[All Fields]) OR "bacterial meningitis"[All Fields] OR ("bacterial"[All Fields] AND "meningitis"[All Fields])) AND ("mortality"[Subheading] OR "mortality"[All Fields] OR "mortality"[MeSH Terms])) AND ("humans"[MeSH Terms] AND (English[lang] OR French[lang] OR German[lang] OR Spanish[lang]))
**Google Scholar, first 100 results, searched on January 5, 2024**	(bacterial AND meningitis) AND (mortality OR "case fatality") -animal, until 2022

Supplementary Table 2. R commands used in the meta-analysis and meta-regression.

- Balduzzi S, Rücker G, Schwarzer G (2019). "How to perform a meta-analysis with R: a practical tutorial." Evidence-Based Mental Health, 153-160
- Bates D, Maechler M, Jagan M (2023). Matrix: Sparse and Dense Matrix Classes and Methods. R package version 1.6-1.1, https://CRAN.R-project.org/package=Matrix
- Gilbert P, Varadhan R (2019). numDeriv: Accurate Numerical Derivatives. R package version 2016.8-1.1, https://CRAN.R-project.org/package=numDeriv
- Grolemund G, Wickham H (2011). "Dates and Times Made Easy with lubridate." Journal of Statistical Software, *40*(3), 1-25. https://www.jstatsoft.org/v40/i03/.
- Müller K, Wickham H (2023). tibble: Simple Data Frames. R package version 3.2.1, https://CRAN.R-project.org/package=tibble.
- Pebesma E, Bivand R (2023). Spatial Data Science: With applications in R. Chapman and Hall/CRC. https://doi.org/10.1201/9780429459016; https://r-spatial.org/book/. Pebesma E (2018). "Simple Features for R: Standardized Support for Spatial Vector Data." The R Journal, *10*(1), 439-446. https://doi.org/10.32614/RJ-2018-009.
- R Core Team (2023). R: A Language and Environment for Statistical Computing. R Foundation for Statistical Computing, Vienna, Austria. https://www.R-project.org/.
- Sjoberg D, Whiting K, Curry M, Lavery J, Larmarange J (2021). "Reproducible Summary Tables with the gtsummary Package." The R Journal, *13*, 570-580. https://doi.org/10.32614/RJ-2021-053; https://doi.org/10.32614/RJ-2021-053.
- Viechtbauer W (2010). "Conducting meta-analyses in R with the metafor package." Journal of Statistical Software, *36*(3), 1-48. https://doi.org/10.18637/jss.v036.i03; https://doi.org/10.18637/jss.v036.i03.
- White T, Noble D, Senior A, Hamilton W, Viechtbauer W (2022). metadat: Meta-Analysis Datasets. R package version 1.2-0, https://CRAN.R-project.org/package=metadat.
- Wickham H (2016). ggplot2: Elegant Graphics for Data Analysis. Springer-Verlag New York. ISBN 978-3-319-24277-4, https://ggplot2.tidyverse.org.
- Wickham H (2023). forcats: Tools for Working with Categorical Variables (Factors). R package version 1.0.0, https://CRAN.R-project.org/package=forcats.
- Wickham H (2023). httr: Tools for Working with URLs and HTTP. R package version 1.4.7, https://CRAN.R-project.org/package=httr.
- Wickham H (2023). stringr: Simple, Consistent Wrappers for Common String Operations. R package version 1.5.1, https://CRAN.R-project.org/package=stringr.
- Wickham H, Averick M, Bryan J, Chang W, McGowan LD, François R, Grolemund G, Hayes A, Henry L, Hester J, Kuhn M, Pedersen TL, Miller E, Bache SM, Müller K, Ooms J, Robinson D, Seidel DP, Spinu V, Takahashi K, Vaughan D, Wilke C, Woo K, Yutani H (2019). "Welcome to the tidyverse." Journal of Open Source Software, *4*(43), 1686. https://doi.org/10.21105/joss.01686.
- Wickham H, François R, Henry L, Müller K, Vaughan D (2023). dplyr: A Grammar of Data Manipulation. R package version 1.1.4, https://CRAN.R-project.org/package=dplyr.
- Wickham H, Henry L (2023). purrr: Functional Programming Tools. R package version 1.0.2, https://CRAN.R-project.org/package=purrr.
- Wickham H, Hester J, Bryan J (2024). readr: Read Rectangular Text Data. R package version 2.1.5, https://CRAN.R-project.org/package=readr.
- - Wickham H, Vaughan D, Girlich M (2024). tidyr: Tidy Messy Data. R package version 1.3.1, https://CRAN.R-project.org/package=tidyr.

Supplementary Table 3. Characteristics of studies included.

First author, reference	Year	Country	Inclusion period	Age group	Patients	Deaths	More than one study period
**Brainerd** [1]	1947	United States	1943 - 1946	Not specified	250	73	No
**Smith** [2]	1954	United States	1944 - 1953	Neonates and children	354	88	No
**Watson** [3]	1957	Multinational	1949 - 1955	Neonates	45	29	Yes, country
**Shaper** [4]	1958	Uganda	1957 - 1957	Not specified	110	59	No
**Eigler** [5]	1961	United States	1948 - 1958	Not specified	294	50	No
**Esrachowitz** [6]	1961	South Africa	1955 - 1957	Not specified	303	28	Yes, age group
**Groover** [7]	1961	United States	1948 - 1959	Neonates	39	26	No
**Carpenter** [8]	1962	United States	1950 - 1960	Not specified	209	83	No
**Quaade** [9]	1962	Denmark	1949 - 1959	Not specified	658	75	No
**Yu** [10]	1963	Australia	1953 - 1961	Neonates	47	28	No
**Gossage** [11]	1964	Canada	1942 - 1962	Children	255	66	Yes, observation period
**Heycock** [12]	1964	United Kingdom	1950 - 1962	Neonates and children	337	29	Yes, age group
**Wilson** [13]	1964	United States	1942 - 1963	Children and adults	94	39	Yes, observation period
**Schmuziger** [14]	1965	Switzerland	1950 - 1962	Not specified	122	32	No
**Swartz** [15]	1965	United States	1956 - 1962	Not specified	207	49	No
**Berman** [16]	1966	United States	1958 - 1965	Neonates	29	28	No
**Fortuine** [17]	1966	United States	1957 - 1964	Not specified	69	21	No
**McNiel** [18]	1966	Saudi Arabia	1956 - 1964	Neonates and children	91	23	No
**Donald** [19]	1968	United Kingdom	NA - NA	Not specified	210	7	No
**Fosson** [20]	1968	United States	1960 - 1966	Neonates	21	16	No
**Chevrie** [21]	1969	France	NA - NA	Neonates	36	22	No
**Justitz** [22]	1970	Switzerland	1955 - 1966	Children	240	19	No
**Overall** [23]	1970	United States	1959 - 1966	Neonates	25	15	No
**Seriki** [24]	1970	Nigeria	1964 - 1966	Neonates and children	156	38	Yes, age group
**Jonsson** [25]	1971	Sweden	1956 - 1967	Not specified	472	89	Yes, observation period
**Kendall** [26]	1971	Zimbabwe	1967 - 1969	Neonates and children	197	78	Yes, age group
**McDonald** [27]	1972	South Africa	1956 - 1970	Neonates	82	41	No
**Wiebe** [28]	1972	United States	1965 - 1965	Children and adults	194	23	No
**Fraser** [29]	1973	United States	1935 - 1946	Not specified	30	20	No

**Fraser** [30]	1973	United States	1961 - 1971	Not specified	260	52	No
**Floyd** [31]	1974	United States	1963 - 1971	Not specified	389	83	No
**Fraser** [32]	1974	United States	1964 - 1971	Not specified	179	42	No
**Santhanakrishnan** [33]	1974	India	1972 - 1973	Neonates	69	34	No
**Chintu** [34]	1975	Zambia	1973 - 1974	Neonates and children	85	35	No
**Fraser** [35]	1975	United States	1967 - 1970	Children	126	10	No
**Hashemi** [36]	1975	Iran	1961 - 1971	Children	160	45	No
**Hodges** [37]	1975	United States	1949 - 1973	Not specified	349	90	No
**Kaiser** [38]	1975	Hungary	1964 - 1973	Children	34	10	No
**Dawson** [39]	1976	New Zealand	NA - NA	Children	53	7	No
**Goldacre** [40]	1976	United Kingdom	1969 - 1973	Neonates and children	738	94	Yes, age group
**Yeung** [41]	1976	Hong Kong	NA - NA	Neonates	20	4	No
**Agranat** [42]	1977	Israel	1954 - 1975	Adults	87	35	No
**Finland** [43]	1977	United States	1947 - 1972	Not specified	365	146	Yes, observation period
**Gilsdorf** [44]	1977	United States	1971 - 1974	Not specified	39	1	No
**Lang** [45]	1977	New Zealand	1971 - 1976	Children	227	20	No
**Moazami** [46]	1977	Iran	1968 - 1975	Neonates and children	383	96	No
**Hailemeskel** [47]	1978	Ethiopia	1975 - 1976	Children	120	26	No
**Bieler** [48]	1979	Switzerland	1967 - 1978	Neonates	22	13	No
**Chattopadhyay** [49]	1980	United Kingdom	1971 - 1978	Not specified	48	9	No
**Geiseler** [50]	1980	United States	1954 - 1976	Not specified	1,289	95	No
**Horwitz** [51]	1980	United States	1967 - 1976	Children	302	10	No
**Perez-Yarza** [52]	1980	Spain	1976 - 1979	Children	140	13	No
**Shann** [53]	1981	Papua New Guinea	1977 - 1979	Children	73	19	No
**Wotton** [54]	1981	Canada	1972 - 1977	Children and adults	37	5	No
**Davey** [55]	1982	United Kingdom	1968 - 1977	Not specified	270	30	No
**Guggenbichler** [56]	1982	Austria	1970 - 1979	Neonates and children	312	50	No
**Helwig** [57]	1982	Germany	1972 - 1982	Children	74	4	No
**Onile** [58]	1982	Nigeria	1976 - 1979	Not specified	447	137	No
**Bohr** [59]	1983	Denmark	1966 - 1976	Not specified	875	93	Yes, age group
**Guirguis** [60]	1983	Egypt	1977 - 1978	Not specified	350	125	No

**Ispahani** [61]	1983	United Kingdom	1974 - 1980	Children and adults	141	28	No
**Gorse** [62]	1984	United States	1970 - 1982	Adults	86	28	No
**McCracken** [63]	1984	United States	1969 - 1982	Neonates and children	1,787	114	No
**Mulder** [64]	1984	Netherlands	1976 - 1982	Neonates	280	75	No
**Mulla** [65]	1984	South Africa	1979 - 1980	Children	358	122	No
**Rodriguez** [66]	1985	Dominican Rep.	1984 - 1985	Children	100	20	No
**Schlech** [67]	1985	United States	1978 - 1981	Not specified	13,974	1,847	No
**Shann** [68]	1985	Papua New Guinea	1979 - 1983	Children	367	97	No
**Skoch** [69]	1985	United States	1979 - 1982	Not specified	130	10	No
**Valmari** [70]	1985	Finland	1978 - 1978	Neonates and children	130	5	No
**Benderly** [71]	1986	Israel	1979 - 1984	Neonates	17	5	No
**Jadavji** [72]	1986	Canada	1979 - 1983	Children	235	15	No
**Rantakallio** [73]	1986	Finland	1966 - 1981	Neonates and children	54	8	No
**Yost** [74]	1986	United States	1978 - 1983	Neonates and children	101	8	No
**Bennhagen** [75]	1987	Sweden	1976 - 1983	Neonates	52	11	Yes, observation period
**Girgis** [76 77]	1987	Egypt	1979 - 1980	Not specified	99	24	No
**Kilpatrick** [78]	1987	Pakistan	1985 - 1985	Neonates	17	11	No
**Mir** [79]	1987	Sweden	1956 - 1975	Children	201	16	Yes, observation period
**Salwen** [80]	1987	Switzerland	1978 - 1982	Adults	46	15	No
**Zimmerli** [81]	1988	New Zealand	1975 - 1987	Children	144	2	No
**Dawson** [82]	1988	Egypt	NA - NA	Children and adults	100	17	Yes, age group
**Bell** [83]	1989	Ireland	1973 - 1986	Neonates	41	20	No
**Bhat** [84]	1989	India	1972 - 1980	Children	256	78	No
**Cisse** [85]	1989	Senegal	1983 - 1988	Neonates and children	409	135	No
**Lim** [86]	1989	Singapore	1984 - 1987	Neonates and children	36	5	Yes, age group
**Martinez** [87]	1989	Spain	1984 - 1986	Adults	200	16	No
**Rosenthal** [88]	1989	Israel	1981 - 1985	Neonates and children	107	13	No
**Sakakihara** [89]	1989	Japan	1940 - 1981	Children	68	37	Yes, observation period
**Shaltout** [90]	1989	Kuwait	1981 - 1987	Neonates and children	92	5	No
**Bryan** [91]	1990	Brazil	1973 - 1982	Not specified	3,973	1,312	Yes, age group
**Carter** [92]	1990	United Kingdom	1946 - 1987	Neonates and children	559	39	Yes, observation period

**Choo** [93]	1990	Malaysia	1985 - 1987	Children	58	11	No
**de Bary** [94]	1990	Côte d'Ivoire	1985 - 1986	Neonates and children	150	49	No
**Pomeroy** [95]	1990	United States	1973 - 1977	Children	191	4	No
**Salih** [96]	1990	Sudan	1985 - 1985	Children	43	8	No
**Wenger** [97]	1990	United States	1986 - 1986	Not specified	2,158	228	No
**Zaki** [98]	1990	Kuwait	1983 - 1988	Neonates	45	10	No
**Zaki** [99]	1990	Kuwait	1981 - 1987	Neonates and children	107	10	Yes, age group
**de Louvois** [100]	1991	Switzerland	1980 - 1986	Adults	104	29	No
**Dufour** [101]	1991	Australia	1984 - 1988	Children	270	16	No
**Hanna** [102]	1991	India	1989 - 1989	Neonates and children	852	137	No
**Kabra** [103]	1991	Australia	1975 - 1989	Neonates	43	9	No
**Minutillo** [104]	1991	Zimbabwe	1987 - 1988	Neonates	94	39	No
**Nathoo** [105]	1991	Nigeria	1986 - 1987	Neonates and children	46	12	No
**Olanrewaju** [106]	1991	Multinational	1989 - 1990	Children and adults	528	157	No
**Pecoul** [107]	1991	United Kingdom	1985 - 1987	Neonates and children	1,810	154	Yes, age group
**Al-Jurayyan** [108]	1992	Saudi Arabia	1982 - 1990	Children	71	5	No
**Craig** [109]	1992	New Zealand	1987 - 1991	Children	62	3	No
**Francis** [110]	1992	Australia	1987 - 1989	Neonates	115	30	No
**Franco** [111]	1992	United States	1993 - 2002	Neonates	26	5	No
**Mackie** [112]	1992	Ghana	1989 - 1990	Children	69	19	No
**Pallangyo** [113]	1992	Tanzania	1989 - 1990	Children and adults	78	19	No
**Rasmussen** [114]	1992	Denmark	1976 - 1988	Adults	42	12	No
**Rothrock** [115]	1992	United States	1979 - 1990	Neonates and children	258	23	No
**Shattuck** [116]	1992	United States	1974 - 1989	Neonates	98	17	No
**Srair** [117]	1992	Saudi Arabia	1988 - 1991	Children	50	6	No
**Tefuarani** [118]	1992	Papua New Guinea	1989 - 1990	Neonates and children	108	18	Yes, age group
**Thomas** [119]	1992	Australia	1979 - 1989	Neonates and children	80	5	Yes, age group
**Airede** [120]	1993	Nigeria	1988 - 1990	Neonates	36	12	No
**Ballantyne** [121]	1993	United Kingdom	1964 - 1991	Not specified	97	19	Yes, observation period
**Boehme** [122]	1993	Chile	1988 - 1991	Children	90	12	No
**Brivet** [123]	1993	France	1981 - 1992	Adults	80	31	No

**Durand** [124]	1993	United States	1962 - 1988	Adults	253	63	No
**Fortnum** [125]	1993	United Kingdom	1980 - 1989	Children	262	25	No
**Liu** [126]	1993	Taiwan	1988 - 1992	Children	41	7	No
**Pfister** [127]	1993	Germany	1984 - 1989	Adults	86	16	No
**Ara** [128]	1994	Spain	1985 - 1988	Not specified	340	35	No
**Carroll** [129]	1994	Vanuatu	1988 - 1991	Not specified	83	13	No
**Chotpitayasunondh** [130]	1994	Thailand	1980 - 1990	Neonates and children	618	129	Yes, age group
**Commey** [131]	1994	Ghana	1991 - 1993	Children	103	22	No
**Dagan** [132]	1994	Israel	1988 - 1991	Neonates and children	467	18	No
**Ford** [133]	1994	South Africa	1991 - 1992	Not specified	85	33	Yes, age group
**Kallio** [134]	1994	Finland	1984 - 1991	Children	325	12	No
**Moreno** [135]	1994	Panama	1975 - 1992	Neonates	107	37	No
**Ozumba** [136]	1994	Nigeria	1989 - 1993	Neonates and children	76	22	Yes, age group
**Synnott** [137]	1994	United Kingdom	1975 - 1991	Neonates	1,846	249	No
**Ali** [138]	1995	Trinidad and Tobago	1988 - 1990	Neonates	50	7	No
**Almirante** [139]	1995	Spain	1985 - 1994	Adults	210	39	No
**Ciana** [140]	1995	Mozambique	1989 - 1989	Children	70	20	No
**Daoud** [141]	1995	Jordan	1990 - 1992	Children	118	13	No
**Gedlu** [142]	1995	Ethiopia	1990 - 1994	Children	132	37	No
**Kaaresen** [143]	1995	Norway	1980 - 1993	Children	92	4	No
**Kilpi** [144]	1995	Finland	1987 - 1991	Children	122	2	No
**Lecour** [145]	1995	Portugal	1981 - 1994	Neonates and children	256	16	Yes, age group
**Lutsar** [146]	1995	Estonia	1983 - 1990	Children	84	3	No
**Patwari** [147]	1995	India	1987 - 1988	Children	60	16	No
**Salaun-Saraux** [148]	1995	Rwanda	1983 - 1990	Neonates and children	262	99	No
**Singhi** [149]	1995	India	NA - NA	Children	50	9	No
**Ahmed** [150]	1996	Sudan	1989 - 1990	Children	56	16	No
**Berg** [151]	1996	Sweden	1987 - 1989	Not specified	921	85	No
**Bergemann** [152]	1996	South Africa	1994 - 1995	Children and adults	64	27	No
**Gomes** [153]	1996	Brazil	1993 - 1993	Neonates and children	276	56	No
**Ichiyama** [154]	1996	Japan	1984 - 1994	Neonates and children	13	1	No

**Ishikawa** [155]	1996	Japan	1984 - 1993	Neonates and children	320	24	Yes, age group
**Laguna** [156]	1996	Spain	1982 - 1995	Adults	77	5	No
**Macaluso** [157]	1996	Brazil	1991 - 1992	Children	179	34	No
**Qazi** [158]	1996	Pakistan	1990 - 1992	Children	89	17	No
**Awasthi** [159]	1997	India	1995 - 1996	Children	97	18	No
**Grobler** [160]	1997	South Africa	1990 - 1995	Children	61	12	No
**Hussey** [161]	1997	South Africa	1991 - 1992	Children	201	10	No
**Imuekehme** [162]	1997	Nigeria	1988 - 1988	Children	40	4	No
**Sigurdardottir** [163]	1997	Iceland	1975 - 1994	Adults	127	25	No
**Sung** [164]	1997	Hong Kong	1984 - 1993	Children	41	2	No
**Chang** [165]	1998	Taiwan	1989 - 1995	Children	101	27	No
**Fernandez-Jaen** [166]	1998	Spain	1986 - 1995	Children	166	6	No
**Gutierrez** [167]	1998	Spain	1995 - 1997	Children and adults	40	4	No
**Honnas** [168]	1998	Kenya	1996 - 1996	Children and adults	32	12	No
**Hussain** [169]	1998	Malaysia	1995 - 1995	Children	71	9	No
**Imananagha** [170]	1998	Nigeria	1991 - 1994	Children	62	14	No
**Kim** [171]	1998	Republic of Korea	1986 - 1995	Children	140	17	No
**Molyneux** [172]	1998	Malawi	1996 - 1997	Neonates and children	260	104	No
**Müller** [173]	1998	Germany	1992 - 1996	Adults	47	8	No
**Rios-Reategui** [174]	1998	Mexico	1990 - 1995	Neonates	31	9	No
**Schutte** [175]	1998	South Africa	NA - NA	Adults	33	7	No
**Shembesh** [176]	1998	Libya	1994 - 1995	Children	77	10	No
**Campagne** [177]	1999	Niger	1989 - 1996	Not specified	4,177	641	No
**Daoud** [178]	1999	Jordan	1993 - 1995	Neonates	52	12	No
**Dawson** [179]	1999	United States	1981 - 1995	Neonates and children	793	27	Yes, age group
**Moyen** [180]	1999	Republic of the Congo	1994 - 1996	Children	138	60	No
**Nathoo** [181]	1999	Zimbabwe	1995 - 1996	Neonates and children	125	41	No
**Okome** [182]	1999	Gabon	1991 - 1995	Adults	85	15	No
**Palmer** [183]	1999	Gambia	1991 - 1994	Neonates and children	420	111	Yes, age group
**Pena** [184]	1999	Venezuela	1996 - 1998	Children	152	12	No
**Silber** [185]	1999	South Africa	1996 - 1996	Adults	12	2	No

**Struillou [186]**	1999	France	1995 - 1998	Children and adults	100	17	Yes, age group
**Tang [187]**	1999	Taiwan	1981 - 1998	Adults	263	70	No
**Chotmongkol [188]**	2000	Thailand	1984 - 1998	Adults	71	26	No
**Gordon [189]**	2000	Malawi	1998 - 1999	Adults	248	123	No
**Klinger [190]**	2000	Canada	1979 - 1998	Neonates	101	13	Yes, observation period
**Moller [191]**	2000	Denmark	1997 - 1999	Adults	17	1	No
**Nel [192]**	2000	South Africa	1981 - 1992	Neonates	88	30	No
**Ray [193]**	2000	India	NA - NA	Children	32	14	No
**Zanelli [194]**	2000	France	1982 - 1997	Neonates	35	4	No
**Almuneef [195]**	2001	Saudi Arabia	1995 - 2000	Neonates and children	76	4	No
**Berkley [196]**	2001	Kenya	1999 - 2000	Children	45	15	No
**Bonsu [197]**	2001	United States	1984 - 1996	Children	288	6	No
**Holt [198]**	2001	United Kingdom	1996 - 1997	Neonates	144	14	No
**Johnson [199]**	2001	Nigeria	1992 - 1996	Neonates and children	62	30	Yes, age group
**Madhi [200]**	2001	South Africa	1997 - 1999	Children	147	29	No
**McMillan [201]**	2001	United States	1970 - 1998	Adults	294	77	No
**Miner [202]**	2001	United States	1987 - 1997	Not specified	118	9	Yes, age group
**Neuman [203]**	2001	United States	1988 - 1998	Children	216	12	No
**Sahai [204]**	2001	India	1994 - 1996	Children	100	25	No
**Weiss [205]**	2001	Brazil	1997 - 1998	Not specified	274	24	No
**Ahsan [206]**	2002	Pakistan	1998 - 2000	Children and adults	68	6	No
**Barboza [207]**	2002	Argentina	1988 - 1998	Adults	87	19	No
**Beyrer [208]**	2002	Germany	1998 - 2000	Not specified	256	20	Yes, age group
**Chan [209]**	2002	Singapore	1993 - 2000	Adults	26	5	No
**Chinchankar [210]**	2002	India	1997 - 1999	Children	54	17	No
**Duke [211]**	2002	India	1998 - 2000	Children	120	20	No
**Hemalatha [212]**	2002	Spain	1997 - 1998	Neonates	82	7	No
**Lopez [213]**	2002	Madagascar	1998 - 2000	Children	83	26	No
**Migliani [214]**	2002	Malawi	1997 - 2001	Children	598	200	No
**Molyneux [215]**	2002	Kenya	1994 - 1998	Neonates and children	223	74	No
**Mwangi [216]**	2002	Netherlands	1988 - 1998	Children	170	2	No

**Oostenbrink** [217]	2003	Saudi Arabia	1999 - 2001	Neonates and children	208	5	No
**Al-Mazrou** [218]	2003	Taiwan	1986 - 2001	Neonates	60	6	Yes, observation period
**Chang** [219]	2003	Papua New Guinea	1997 - 2000	Children	346	59	No
**Flores-Cordero** [220]	2003	Spain	1995 - 2000	Adults	108	9	No
**Kirimi** [221]	2003	Turkey	1999 - 2000	Children	48	6	No
**Rabbani** [222]	2003	Pakistan	1995 - 2002	Adults	192	43	No
**Celal** [223]	2004	Turkey	1996 - 2002	Adults	186	29	No
**Khwannimit** [224]	2004	Thailand	1982 - 2001	Adults	74	11	No
**Luca** [225]	2004	Romania	2000 - 2002	Neonates and children	56	3	No
**Ostergaard** [226]	2004	Denmark	1988 - 2002	Children and adults	54	8	No
**Sallam** [227]	2004	Yemen	2001 - 2002	Neonates and children	20	3	No
**Singhi** [228]	2004	India	1993 - 1996	Children	222	19	No
**van de Beek** [229]	2004	Tanzania	1999 - 2002	Children	130	66	No
**Wiersinga** [230]	2004	Netherlands	1998 - 2002	Adults	696	143	No
**Amsalu** [231]	2005	Ethiopia	1998 - 2003	Children	151	20	No
**Bekondi** [232]	2005	Central African Rep.	1999 - 2003	Adults	60	40	No
**Deeks** [233]	2005	Canada	1994 - 2001	Not specified	7,227	813	No
**Farag** [234]	2005	Egypt	2002 - 2003	Children	202	28	No
**Hui** [235]	2005	Hong Kong	1992 - 2001	Adults	35	11	No
**Lucena** [236]	2005	Brazil	1997 - 1997	Children	83	14	No
**May** [237]	2005	Australia	1992 - 2002	Neonates	78	11	No
**Odetola** [238]	2005	United States	1995 - 2000	Children	334	34	No
**Ogunlesi** [239]	2005	Nigeria	1998 - 2003	Children	124	33	No
**Al Khoransani** [240]	2006	Yemen	1999 - 2001	Children	160	16	No
**Bregani** [241]	2006	Chad	2001 - 2001	Not specified	595	52	No
**Elsaid** [242]	2006	Qatar	1998 - 2002	Neonates and children	64	1	No
**Garges** [243]	2006	United States	1997 - 2004	Neonates and children	73	4	No
**Mbelesso** [244]	2006	Central African Rep.	1998 - 2003	Adults	502	160	No
**Molyneux** [245]	2006	United Kingdom	1984 - 1991	Neonates and children	197	14	No
**Pizon** [246]	2006	United States	1992 - 2003	Adults	38	7	No
**Shabani** [247]	2006	Kuwait	2001 - 2001	Children	42	1	No

**Singhi** [248]	2006	India	NA - NA	Children	16	1	No
**Afifi** [249]	2007	Egypt	1998 - 2004	Children and adults	1,101	239	No
**Boisier** [250]	2007	Niger	2003 - 2006	Not specified	1,935	304	No
**Dauchy** [251]	2007	France	2001 - 2004	Adults	60	13	No
**Faustini** [252]	2007	Italy	1996 - 2000	Not specified	525	98	No
**Johnson** [253]	2007	Nigeria	1998 - 2005	Neonates and children	62	28	No
**Krebs** [254]	2007	Brazil	1994 - 2004	Neonates	87	10	No
**Lepur** [255]	2007	Croatia	1990 - 2004	Adults	286	65	No
**Nguyen** [256]	2007	Vietnam	1996 - 2005	Children and adults	300	31	No
**Peltola** [257]	2007	Multinational	1996 - 2003	Children	654	86	No
**Scarborough** [258]	2007	Malawi	2002 - 2005	Adults	322	162	No
**Theodoridou** [259]	2007	Greece	1974 - 2005	Children	1,331	34	No
**Airede** [260]	2008	Nigeria	1992 - 1995	Neonates	50	17	No
**Bercion** [261]	2008	Central African Rep.	2004 - 2005	Neonates and children	130	51	No
**Franco** [262]	2008	Mexico	1993 - 2002	Children and adults	218	36	No
**Lagunju** [263]	2008	Nigeria	2004 - 2007	Children	97	26	No
**Lazzarini** [264]	2008	Italy	2002 - 2005	Adults	289	20	No
**Mongelluzzo** [265]	2008	United States	2001 - 2006	Neonates and children	2,780	117	No
**Pelkonen** [266]	2008	Angola	2004 - 2004	Children	403	133	No
**Sigauque** [267]	2008	Mozambique	1998 - 2003	Neonates and children	65	23	No
**Cabellos** [268]	2009	Spain	1977 - 2006	Adults	675	124	No
**Dzupova** [269]	2009	Czechia	1997 - 2006	Adults	279	55	No
**Gurley** [270]	2009	Bangladesh	2003 - 2005	Not specified	189	26	No
**Ishihara** [271]	2009	Japan	1998 - 2007	Adults	71	16	No
**Roca** [272]	2009	Mozambique	2006 - 2007	Neonates and children	43	11	No
**Tiskumara** [273]	2009	Multinational	2005 - 2005	Neonates	76	15	No
**Traore** [274]	2009	Multinational	2002 - 2006	Not specified	860	262	Yes, age group
**Abdulrab** [275]	2010	Yemen	2006 - 2007	Adults	59	16	No
**Aletayeb** [276]	2010	Iran	1997 - 2007	Neonates	14	2	No
**Ba** [277]	2010	Senegal	2006 - 2008	Neonates and children	206	79	No
**Bentlin** [278]	2010	Brazil	1997 - 2006	Neonates	21	6	No

**Cho** [279]	2010	Republic of Korea	1996 - 2005	Neonates and children	402	38	No
**Erdem** [280]	2010	Turkey	2001 - 2008	Adults	159	34	No
**Hudeckova** [281]	2010	Slovakia	1997 - 2007	Not specified	1,210	148	Yes, observation period
**Mankhambo** [282]	2010	Malawi	2004 - 2006	Children	211	58	No
**Moon** [283]	2010	Republic of Korea	1998 - 2008	Adults	172	33	No
**Perez** [284]	2010	Cuba	1998 - 2007	Not specified	4,798	1,157	No
**Su** [285]	2010	Taiwan	1986 - 2007	Adults	217	90	No
**Talbert** [286]	2010	Kenya	2001 - 2009	Neonates	152	29	No
**Vibha** [287]	2010	India	2004 - 2008	Children and adults	380	34	No
**Ajdukiewicz** [288]	2011	Malawi	2006 - 2008	Adults	116	64	No
**Pelkonen** [289]	2011	Angola	2005 - 2008	Children	723	272	No
**Thigpen** [290]	2011	United States	1998 - 2007	Not specified	3,157	466	No
**Vashishtha** [291]	2011	India	2009 - 2010	Children	67	7	No
**World Health Organization** [292]	2011	Multinational	2010 - 2010	Not specified	22,831	2,415	No
**Fonseca de Souza** [293]	2012	Brazil	2004 - 2009	Not specified	1,049	168	No
**Juganariu** [294]	2012	Romania	2005 - 2010	Neonates and children	100	5	No
**Kra** [295]	2012	Côte d'Ivoire	2004 - 2004	Adults	15	5	No
**Namani** [296]	2012	Kosovo	1997 - 2002	Neonates and children	257	12	No
**Nansera** [297]	2012	Uganda	2003 - 2008	Neonates and children	51	23	No
**Tarvij Eslami** [298]	2012	Iran	2005 - 2007	Neonates	60	10	No
**Vazquez** [299]	2012	Argentina	2009 - 2009	Adults	13	3	No
**Ben Hamouda** [300]	2013	Tunisia	1996 - 2010	Neonates	44	7	No
**Butsashvili** [301]	2013	Georgia	2006 - 2010	Not specified	100	9	No
**Ergaz** [302]	2013	Israel	1993 - 2009	Neonates	109	8	No
**Kavuncuoglu** [303]	2013	Turkey	2003 - 2010	Neonates	325	8	No
**Khowaja** [304]	2013	Pakistan	2008 - 2011	Neonates and children	188	64	No
**Mahmoudi** [305]	2013	Iran	2009 - 2011	Children	20	2	No
**Porobic** [306]	2013	Bosnia and Herz.	1999 - 2009	Children	140	3	No
**Scott** [307]	2013	Mongolia	2002 - 2010	Children	254	24	No
**Snaebjarnardottir** [308]	2013	Iceland	1975 - 2010	Neonates and children	477	21	No
**Teleb** [309]	2013	Multinational	2004 - 2010	Children	1,263	121	No

**Banajeh** [310]	2014	Yemen	2000 - 2010	Children	2,280	165	Yes, observation period
**Bodilsen** [311]	2014	Denmark	1998 - 2012	Adults	172	33	Yes, observation period
**Levy** [312]	2014	France	2001 - 2012	Neonates and children	4,808	414	Yes, age group
**Molyneux** [313]	2014	Malawi	2008 - 2012	Children and adults	360	93	No
**Namani** [314]	2014	Kosovo	2000 - 2010	Children and adults	83	8	Yes, observation period and age group
**Okike** [315]	2014	United Kingdom	2010 - 2011	Neonates	282	22	No
**Thornorethardottir** [316]	2014	Iceland	1995 - 2010	Adults	111	18	No
**Correa-Lima** [317]	2015	Brazil	2004 - 2008	Children	270	34	No
**Hu** [318]	2015	China	2012 - 2013	Children	25	4	No
**Kamoun** [319]	2015	Tunisia	1990 - 2012	Neonates	55	22	No
**Lin** [320]	2015	Taiwan	1984 - 2012	Neonates and children	291	50	No
**Mora Mora** [321]	2015	Argentina	2003 - 2013	Adults	69	28	No
**Olson** [322]	2015	Guatemala	2000 - 2007	Neonates and children	800	192	No
**Shresta** [323]	2015	Nepal	2012 - 2013	Neonates and children	18	6	No
**Softic** [324]	2015	Bosnia and Herz.	2012 - 2013	Neonates	18	2	No
**Tan** [325]	2015	China	2008 - 2014	Neonates	232	7	No
**Bari** [326]	2016	Pakistan	2012 - 2012	Children	199	20	No
**Baunbaek** [327]	2016	Denmark	2003 - 2010	Adults	147	49	No
**Coldiron** [328]	2016	Niger	2015 - 2015	Not specified	473	70	No
**Glimaker** [329]	2016	Sweden	1995 - 2014	Adults	1,746	214	Yes, observation period
**Kambire** [330]	2016	Burkina Faso	2011 - 2013	Not specified	2,858	472	No
**Wee** [331]	2016	Singapore	1998 - 2013	Neonates and children	112	7	No
**Gudina** [332]	2017	Ethiopia	2013 - 2015	Adults	64	15	No
**Hasbun** [333]	2017	United States	2011 - 2014	Adults	3,692	299	No
**Kaburi** [334]	2017	Ghana	2010 - 2015	Not specified	83	10	No
**Kafle** [335]	2017	Nepal	2015 - 2016	Not specified	21	1	No
**Lien** [336]	2017	Taiwan	2006 - 2015	Adults	50	20	No
**Ouchenir** [337]	2017	Canada	2013 - 2014	Neonates	113	8	No
**Park** [338]	2017	Republic of Korea	2009 - 2016	Not specified	80	13	No
**Polkowska** [339]	2017	Finland	2004 - 2014	Not specified	633	65	No
**Sadeq** [340]	2017	Kuwait	2010 - 2014	Children	57	5	No

**Wall** [341]	2017	Malawi	2012 - 2013	Adults	117	66	No
**Amare** [342]	2018	Ethiopia	2011 - 2013	Children	80	6	No
**Jumanne** [343]	2018	Tanzania	2011 - 2012	Children	13	5	No
**Kumar** [344]	2018	India	NA - NA	Neonates	89	10	No
**Brown** [345]	2019	United States	2015 - 2016	Adults	14	1	No
**De Almeida** [346]	2019	Brazil	2006 - 2017	Not specified	49	9	No
**El-Naggar** [347]	2019	Canada	2010 - 2016	Neonates	246	31	No
**Fuentes-Antras** [348]	2019	Spain	2007 - 2014	Adults	79	12	No
**Haydar** [349]	2019	Lebanon	2008 - 2016	Not specified	46	4	No
**Larsen** [350]	2019	Denmark	2015 - 2017	Adults	379	50	No
**Mwenda** [351]	2019	Multinational	2011 - 2016	Children	1,670	186	No
**Pruitt** [352]	2019	United States	2011 - 2016	Neonates	71	2	No
**Sonko** [353]	2019	Senegal	2010 - 2016	Neonates and children	115	18	No
**Tagbo** [354]	2019	Nigeria	2010 - 2016	Neonates and children	153	23	No
**Tsolenyanu** [355]	2019	Togo	2010 - 2016	Neonates and children	98	21	No
**Huang** [356]	2020	Taiwan	2007 - 2013	Neonates	12	0	No
**Johansson** [357]	2020	Sweden	1986 - 2015	Children	101	6	No
**Liu** [358]	2020	China	2016 - 2018	Neonates	111	5	No
**Loutfi** [359]	2020	Morocco	2014 - 2018	Not specified	35	10	No
**Matulyte** [360]	2020	Lithuania	2009 - 2016	Adults	159	9	No
**Peros** [361]	2020	Netherlands	2004 - 2016	Neonates	45	15	No
**Pomar** [362]	2020	Spain	1982 - 2017	Adults	715	124	No
**Tubiana** [363]	2020	France	2013 - 2015	Adults	533	90	No
**Adil** [364]	2021	United States	2008 - 2015	Neonates and children	1,632	62	No
**Aimbudlop** [365]	2021	Thailand	2013 - 2017	Adults	28	3	No
**Bumburidi** [366]	2021	Kazakhstan	2017 - 2018	Not specified	37	2	No
**Kumar** [367]	2021	India	2018 - 2019	Adults	39	17	No
**Pelkonen** [368]	2021	Angola	2016 - 2017	Neonates	139	34	No
**Salmanov** [369]	2021	Ukraine	2017 - 2019	Neonates	86	28	No
**Savonius** [370]	2021	Angola	2012 - 2017	Children	375	148	No

**Sunwoo** [371]	2021	Republic of Korea	2007 - 2016	Adults	43	5	No
**Wong** [372]	2021	Hong Kong	2004 - 2019	Neonates	139	4	No

Supplementary Table 4. Characteristics of studies reporting sex proportions among patients with bacterial meningitis.

First author, reference	Year	Country	Inclusion period	Age group	Total patients*	Male patients	Female patients	Proportion of male patients
**Shaper** [4]	1958	Uganda	1957–1957	Not specified	110	77	41	0.70
**Quaade** [9]	1962	Denmark	1949–1959	Not specified	658	387	271	0.59
**Schmuziger** [14]	1965	Switzerland	1950–1962	Not specified	122	76	46	0.62
**Berman** [16]	1966	United States	1958–1965	Neonates	29	22	7	0.76
**McNiel** [18]	1966	Saudi Arabia	1956–1964	Neonates and children	91	53	38	0.58
**Justitz** [22]	1970	Switzerland	1955–1966	Children	240	138	102	0.58
**Overall** [23]	1970	United States	1959–1966	Neonates	25	13	12	0.52
**McDonald** [27]	1972	South Africa	1956–1970	Neonates	82	44	38	0.54
**Wiebe** [28]	1972	United States	1965–1965	Children and adults	194	91	103	0.47
**Floyd** [31]	1974	United States	1963–1971	Not specified	389	189	149	0.49
**Fraser** [32]	1974	United States	1964–1971	Not specified	179	102	77	0.57
**Santhanakrishnan** [33]	1974	India	1972–1973	Neonates	69	46	23	0.67
**Chintu** [34]	1975	Zambia	1973–1974	Neonates and children	85	55	30	0.65
**Hashemi** [36]	1975	Iran	1961–1971	Children	160	96	64	0.60
**Hodges** [37]	1975	United States	1949–1973	Not specified	349	198	151	0.57
**Kaiser** [38]	1975	Hungary	1964–1973	Children	34	22	12	0.65
**Dawson** [39]	1976	New Zealand	NA–NA	Children	53	33	20	0.62
**Goldacre** [40]	1976	United Kingdom	1969–1973	Neonates and children	738	434	304	0.59
**Yeung** [41]	1976	Hong Kong	NA–NA	Neonates	20	15	5	0.75
**Agranat** [42]	1977	Israel	1954–1975	Adults	87	83	4	0.95
**Finland** [43]	1977	United States	1947–1972	Not specified	365	193	172	0.53
**Lang** [45]	1977	New Zealand	1971–1976	Children	227	121	78	0.53
**Hailemeskel** [47]	1978	Ethiopia	1975–1976	Children	120	71	49	0.59
**Bieler** [48]	1979	Switzerland	1967–1978	Neonates	22	14	8	0.64
**Chattopadhyay** [49]	1980	United Kingdom	1971–1978	Not specified	48	33	34	0.69
**Geiseler** [50]	1980	United States	1954–1976	Not specified	1,289	715	591	0.55
**Perez-Yarza** [52]	1980	Spain	1976–1979	Children	140	83	57	0.59

* Some studies did not report sex proportions for all study periods or all subgroups; therefore, the total patients may diverge from the sum of male plus female patients.

**Davey** [55]	1982	United Kingdom	1968–1977	Not specified	270	162	108	0.60
**Bohr** [59]	1983	Denmark	1966–1976	Not specified	875	486	389	0.56
**Guirguis** [60]	1983	Egypt	1977–1978	Not specified	350	200	150	0.57
**Mulder** [64]	1984	Netherlands	1976–1982	Neonates	280	160	120	0.57
**Rodriguez** [66]	1985	Dominican Rep.	1984–1985	Children	100	62	38	0.62
**Schlech** [67]	1985	United States	1978–1981	Not specified	13,974	7,127	6,847	0.51
**Jadavji** [72]	1986	Canada	1979–1983	Children	235	117	118	0.50
**Bennhagen** [75]	1987	Sweden	1976–1983	Neonates	52	28	24	0.54
**Salwen** [80]	1987	Sweden	1956–1975	Children	201	103	98	0.51
**Zimmerli** [81]	1987	Switzerland	1978–1982	Adults	46	23	23	0.50
**Dawson** [82]	1988	New Zealand	1975–1987	Children	144	70	73	0.49
**Girgis** [76 77]	1988	Egypt	NA–NA	Children and adults	100	71	29	0.71
**Bell** [83]	1989	Ireland	1973–1986	Neonates	41	19	22	0.46
**Bhat** [84]	1989	India	1972–1980	Children	256	152	104	0.59
**Lim** [86]	1989	Singapore	1984–1987	Neonates and children	36	22	14	0.61
**Bryan** [91]	1990	Brazil	1973–1982	Not specified	3,973	2,384	1,589	0.60
**Choo** [93]	1990	Malaysia	1985–1987	Children	58	39	19	0.67
**de Bary** [94]	1990	United States	1973–1977	Children	191	113	78	0.59
**Pomeroy** [95]	1990	United States	1986–1986	Not specified	2,158	1,122	1,036	0.52
**Wenger** [97]	1990	Kuwait	1983–1988	Neonates	45	25	20	0.56
**Zaki** [98]	1990	Kuwait	1981–1987	Neonates and children	107	63	47	0.59
**Zaki** [99]	1990	Côte d'Ivoire	1985–1986	Neonates and children	150	79	71	0.53
**de Louvois** [100]	1991	Australia	1984–1988	Children	270	149	122	0.55
**Hanna** [102]	1991	Zimbabwe	1987–1988	Neonates	94	57	34	0.61
**Nathoo** [105]	1991	Nigeria	1986–1987	Neonates and children	46	32	14	0.70
**Olanrewaju** [106]	1991	United Kingdom	1985–1987	Neonates and children	1,810	1,050	760	0.58
**Al-Jurayyan** [108]	1992	Saudi Arabia	1982–1990	Children	71	48	23	0.68
**Craig** [109]	1992	New Zealand	1987–1991	Children	62	29	33	0.47
**Franco** [111]	1992	United States	1993–2002	Neonates	26	9	17	0.35
**Pallangyo** [113]	1992	Tanzania	1989–1990	Children and adults	78	48	30	0.62
**Rasmussen** [114]	1992	Denmark	1976–1988	Adults	42	17	31	0.40

**Rothrock** [115]	1992	United States	1979–1990	Neonates and children	258	148	110	0.57
**Srair** [117]	1992	Saudi Arabia	1988–1991	Children	50	34	16	0.68
**Thomas** [119]	1992	Australia	1979–1989	Neonates and children	80	40	39	0.50
**Airede** [120]	1993	Nigeria	1988–1990	Neonates	36	23	13	0.64
**Ballantyne** [121]	1993	United Kingdom	1964–1991	Not specified	97	53	44	0.55
**Boehme** [122]	1993	Chile	1988–1991	Children	90	52	38	0.58
**Liu** [126]	1993	Taiwan	1988–1992	Children	41	29	12	0.71
**Pfister** [127]	1993	Germany	1984–1989	Adults	86	47	39	0.55
**Chotpitayasunondh** [130]	1994	Thailand	1980–1990	Neonates and children	618	352	266	0.57
**Commey** [131]	1994	Ghana	1991–1993	Children	103	59	44	0.57
**Ford** [133]	1994	South Africa	1991–1992	Not specified	85	47	38	0.55
**Kallio** [134]	1994	Finland	1984–1991	Children	325	160	165	0.49
**Ozumba** [136]	1994	Nigeria	1989–1993	Neonates and children	76	51	25	0.67
**Synnott** [137]	1994	United Kingdom	1975–1991	Neonates	1,846	1,052	794	0.57
**Ali** [138]	1995	Trinidad and Tobago	1988–1990	Neonates	50	34	20	0.68
**Almirante** [139]	1995	Spain	1985–1994	Adults	210	139	71	0.66
**Daoud** [141]	1995	Jordan	1990–1992	Children	118	75	46	0.64
**Gedlu** [142]	1995	Ethiopia	1990–1994	Children	132	86	46	0.65
**Lecour** [145]	1995	Portugal	1981–1994	Neonates and children	256	137	119	0.54
**Patwari** [147]	1995	India	1987–1988	Children	60	45	15	0.75
**Singhi** [149]	1995	India	NA–NA	Children	50	32	18	0.64
**Ahmed** [150]	1996	Sudan	1989–1990	Children	56	31	25	0.55
**Bergemann** [152]	1996	South Africa	1994–1995	Children and adults	64	40	24	0.63
**Gomes** [153]	1996	Brazil	1993–1993	Neonates and children	276	155	121	0.56
**Laguna** [156]	1996	Spain	1982–1995	Adults	77	52	25	0.68
**Macaluso** [157]	1996	Brazil	1991–1992	Children	179	100	79	0.56
**Qazi** [158]	1996	Pakistan	1990–1992	Children	89	54	35	0.61
**Awasthi** [159]	1997	India	1995–1996	Children	97	75	22	0.77
**Hussey** [161]	1997	South Africa	1991–1992	Children	201	155	46	0.77
**Chang** [165]	1998	Taiwan	1989–1995	Children	101	66	35	0.65
**Fernandez-Jaen** [166]	1998	Spain	1986–1995	Children	166	109	57	0.66

**Gutierrez** [167]	1998	Spain	1995–1997	Children and adults	40	13	27	0.33
**Honnas** [168]	1998	Kenya	1996–1996	Children and adults	32	19	13	0.59
**Hussain** [169]	1998	Malaysia	1995–1995	Children	71	41	30	0.58
**Kim** [171]	1998	Republic of Korea	1986–1995	Children	140	75	65	0.54
**Molyneux** [172]	1998	Malawi	1996–1997	Neonates and children	260	140	120	0.54
**Rios-Reategui** [174]	1998	Mexico	1990–1995	Neonates	31	20	11	0.65
**Shembesh** [176]	1998	Libya	1994–1995	Children	77	41	36	0.53
**Campagne** [177]	1999	Niger	1989–1996	Not specified	4,177	2,506	1,671	0.60
**Moyen** [180]	1999	Republic of the Congo	1994–1996	Children	138	86	52	0.62
**Okome** [182]	1999	Gabon	1991–1995	Adults	85	60	25	0.71
**Palmer** [183]	1999	Gambia	1991–1994	Neonates and children	420	229	191	0.55
**Pena** [184]	1999	Venezuela	1996–1998	Children	152	106	46	0.70
**Struillou** [186]	1999	France	1995–1998	Children and adults	100	42	42	0.42
**Tang** [187]	1999	Taiwan	1981–1998	Adults	263	174	89	0.66
**Klinger** [190]	2000	Canada	1979–1998	Neonates	101	58	43	0.57
**Moller** [191]	2000	Denmark	1997–1999	Adults	17	8	9	0.47
**Nel** [192]	2000	South Africa	1981–1992	Neonates	88	51	37	0.58
**Zanelli** [194]	2000	France	1982–1997	Neonates	35	15	20	0.43
**Holt** [198]	2001	United Kingdom	1996–1997	Neonates	144	79	65	0.55
**Johnson** [199]	2001	Nigeria	1992–1996	Neonates and children	62	39	23	0.63
**Madhi** [200]	2001	South Africa	1997–1999	Children	147	77	70	0.52
**McMillan** [201]	2001	United States	1970–1998	Adults	294	152	142	0.52
**Neuman** [203]	2001	United States	1988–1998	Children	216	112	104	0.52
**Sahai** [204]	2001	India	1994–1996	Children	100	62	38	0.62
**Weiss** [205]	2001	Brazil	1997–1998	Not specified	274	160	114	0.58
**Ahsan** [206]	2002	Pakistan	1998–2000	Children and adults	68	60	8	0.88
**Chan** [209]	2002	Singapore	1993–2000	Adults	26	19	7	0.73
**Chinchankar** [210]	2002	India	1997–1999	Children	54	28	26	0.52
**Hemalatha** [212]	2002	India	1998–2000	Children	120	72	48	0.60
**Lopez** [213]	2002	Spain	1997–1998	Neonates	82	44	38	0.54
**Migliani** [214]	2002	Madagascar	1998–2000	Children	83	42	41	0.51

**Molyneux** [215]	2002	Malawi	1997–2001	Children	598	337	261	0.56
**Mwangi** [216]	2002	Kenya	1994–1998	Neonates and children	223	127	96	0.57
**Al-Mazrou** [218]	2003	Saudi Arabia	1999–2001	Neonates and children	208	117	91	0.56
**Chang** [219]	2003	Taiwan	1986–2001	Neonates	60	33	27	0.55
**Duke** [211]	2003	Papua New Guinea	1997–2000	Children	346	185	161	0.53
**Kirimi** [221]	2003	Turkey	1999–2000	Children	48	29	19	0.60
**Rabbani** [222]	2003	Pakistan	1995–2002	Adults	192	146	48	0.76
**Celal** [223]	2004	Turkey	1996–2002	Adults	186	110	76	0.59
**Ostergaard** [226]	2004	Denmark	1988–2002	Children and adults	54	27	27	0.50
**Sallam** [227]	2004	Yemen	2001–2002	Neonates and children	20	14	7	0.70
**van de Beek** [229]	2004	Tanzania	1999–2002	Children	130	65	65	0.50
**Wiersinga** [230]	2004	Netherlands	1998–2002	Adults	696	345	351	0.50
**Odetola** [238]	2005	United States	1995–2000	Children	334	190	144	0.57
**Ogunlesi** [239]	2005	Nigeria	1998–2003	Children	124	88	36	0.71
**Al Khoransani** [240]	2006	Yemen	1999–2001	Children	160	96	64	0.60
**Bregani** [241]	2006	Chad	2001–2001	Not specified	595	355	240	0.60
**Elsaid** [242]	2006	Qatar	1998–2002	Neonates and children	64	35	29	0.55
**Molyneux** [245]	2006	United Kingdom	1984–1991	Neonates and children	197	109	85	0.55
**Pizon** [246]	2006	United States	1992–2003	Adults	38	16	22	0.42
**Singhi** [248]	2006	India	NA–NA	Children	16	15	1	0.94
**Afifi** [249]	2007	Egypt	1998–2004	Children and adults	1,101	655	446	0.59
**Dauchy** [251]	2007	France	2001–2004	Adults	60	34	26	0.57
**Faustini** [252]	2007	Italy	1996–2000	Not specified	525	287	238	0.55
**Johnson** [253]	2007	Nigeria	1998–2005	Neonates and children	62	39	23	0.63
**Krebs** [254]	2007	Brazil	1994–2004	Neonates	87	37	50	0.43
**Lepur** [255]	2007	Croatia	1990–2004	Adults	286	179	107	0.63
**Nguyen** [256]	2007	Vietnam	1996–2005	Children and adults	300	219	81	0.73
**Peltola** [257]	2007	Multinational	1996–2003	Children	654	377	275	0.58
**Scarborough** [258]	2007	Malawi	2002–2005	Adults	322	158	164	0.49
**Theodoridou** [259]	2007	Greece	1974–2005	Children	1,331	785	546	0.59
**Airede** [260]	2008	Nigeria	1992–1995	Neonates	50	32	18	0.64

**Franco** [262]	2008	Mexico	1993–2002	Children and adults	218	131	87	0.60
**Lagunju** [263]	2008	Nigeria	2004–2007	Children	97	62	35	0.64
**Lazzarini** [264]	2008	Italy	2002–2005	Adults	289	156	133	0.54
**Mongelluzzo** [265]	2008	United States	2001–2006	Neonates and children	2,780	1,583	1,197	0.57
**Pelkonen** [266]	2008	Angola	2004–2004	Children	403	222	181	0.55
**Sigauque** [267]	2008	Mozambique	1998–2003	Neonates and children	65	36	29	0.55
**Dzupova** [269]	2009	Czechia	1997–2006	Adults	279	157	122	0.56
**Gurley** [270]	2009	Bangladesh	2003–2005	Not specified	189	136	53	0.72
**Ishihara** [271]	2009	Japan	1998–2007	Adults	71	45	26	0.63
**Bentlin** [278]	2010	Brazil	1997–2006	Neonates	21	13	8	0.62
**Cho** [279]	2010	Republic of Korea	1996–2005	Neonates and children	402	231	169	0.57
**Erdem** [280]	2010	Turkey	2001–2008	Adults	159	90	69	0.57
**Mankhambo** [282]	2010	Malawi	2004–2006	Children	211	116	95	0.55
**Moon** [283]	2010	Republic of Korea	1998–2008	Adults	172	96	76	0.56
**Perez** [284]	2010	Cuba	1998–2007	Not specified	4,798	2,783	2,015	0.58
**Su** [285]	2010	Taiwan	1986–2007	Adults	217	137	80	0.63
**Vibha** [287]	2010	India	2004–2008	Children and adults	380	322	58	0.85
**Pelkonen** [289]	2011	Angola	2005–2008	Children	723	386	340	0.53
**Juganariu** [294]	2012	Romania	2005–2010	Neonates and children	100	58	42	0.58
**Namani** [296]	2012	Kosovo	1997–2002	Neonates and children	257	162	115	0.63
**Tarvij Eslami** [298]	2012	Iran	2005–2007	Neonates	60	33	27	0.55
**Vazquez** [299]	2012	Argentina	2009–2009	Adults	13	7	6	0.54
**Butsashvili** [301]	2013	Georgia	2006–2010	Not specified	100	54	46	0.54
**Kavuncuoglu** [303]	2013	Turkey	2003–2010	Neonates	325	185	140	0.57
**Mahmoudi** [305]	2013	Iran	2009–2011	Children	20	14	6	0.70
**Porobic** [306]	2013	Bosnia and Herz.	1999–2009	Children	140	79	61	0.56
**Snaebjarnardottir** [308]	2013	Iceland	1975–2010	Neonates and children	477	243	234	0.51
**Banajeh** [310]	2014	Yemen	2000–2010	Children	2,280	1,529	747	0.67
**Bodilsen** [311]	2014	Denmark	1998–2012	Adults	172	84	88	0.49
**Thornorethardottir** [316]	2014	Iceland	1995–2010	Adults	111	63	48	0.57
**Correa-Lima** [317]	2015	Brazil	2004–2008	Children	270	148	122	0.55

**Kamoun** [319]	2015	Tunisia	1990–2012	Neonates	55	35	20	0.64
**Lin** [320]	2015	Taiwan	1984–2012	Neonates and children	291	183	108	0.63
**Mora Mora** [321]	2015	Argentina	2003–2013	Adults	69	24	45	0.35
**Olson** [322]	2015	Guatemala	2000–2007	Neonates and children	800	468	332	0.59
**Shresta** [323]	2015	Nepal	2012–2013	Neonates and children	18	9	9	0.50
**Softic** [324]	2015	Bosnia and Herz.	2012–2013	Neonates	18	15	3	0.83
**Tan** [325]	2015	China	2008–2014	Neonates	232	88	144	0.38
**Bari** [326]	2016	Pakistan	2012–2012	Children	199	127	72	0.64
**Baunbaek** [327]	2016	Denmark	2003–2010	Adults	147	80	67	0.54
**Coldiron** [328]	2016	Niger	2015–2015	Not specified	473	220	149	0.47
**Glimaker** [329]	2016	Sweden	1995–2014	Adults	1,746	1,066	1,095	0.61
**Wee** [331]	2016	Singapore	1998–2013	Neonates and children	112	59	63	0.53
**Gudina** [332]	2017	Ethiopia	2013–2015	Adults	64	41	23	0.64
**Kaburi** [334]	2017	Ghana	2010–2015	Not specified	83	51	32	0.61
**Park** [338]	2017	Republic of Korea	2009–2016	Not specified	80	49	31	0.61
**Amare** [342]	2018	Ethiopia	2011–2013	Children	80	52	28	0.65
**Jumanne** [343]	2018	Tanzania	2011–2012	Children	13	9	4	0.69
**Kumar** [344]	2018	India	NA–NA	Neonates	89	76	13	0.85
**Brown** [345]	2019	United States	2015–2016	Adults	14	6	8	0.43
**De Almeida** [346]	2019	Brazil	2006–2017	Not specified	49	23	26	0.47
**Fuentes-Antras** [348]	2019	Spain	2007–2014	Adults	79	46	33	0.58
**Larsen** [350]	2019	Denmark	2015–2017	Adults	379	201	178	0.53
**Huang** [356]	2020	Taiwan	2007–2013	Neonates	12	6	6	0.50
**Johansson** [357]	2020	Sweden	1986–2015	Children	101	54	47	0.53
**Liu** [358]	2020	China	2016–2018	Neonates	111	54	57	0.49
**Loutfi** [359]	2020	Morocco	2014–2018	Not specified	35	19	16	0.54
**Matulyte** [360]	2020	Lithuania	2009–2016	Adults	159	82	77	0.52
**Peros** [361]	2020	Netherlands	2004–2016	Neonates	45	25	20	0.56
**Pomar** [362]	2020	Spain	1982–2017	Adults	715	351	364	0.49
**Tubiana** [363]	2020	France	2013–2015	Adults	533	294	239	0.55
**Adil** [364]	2021	United States	2008–2015	Neonates and children	1,632	923	709	0.57

**Aimbudlop** [365]	2021	Thailand	2013–2017	Adults	28	15	13	0.54
**Savonius** [370]	2021	Angola	2012–2017	Children	375	216	157	0.58
**Sunwoo** [371]	2021	Republic of Korea	2007–2016	Adults	43	26	17	0.60
**Wong** [372]	2021	Hong Kong	2004–2019	Neonates	139	75	64	0.54

Supplementary Table 5. Characteristics of studies reporting sex-specific case fatality ratios (CFRs)

First author, reference	Year	Country	Inclusion period	Age group	Male patients	Deaths in males	Female patients	Deaths in females	CFR in males	CFR in females
**Shaper** [4]	1958	Uganda	1957–1957	Not specified	77	45	41	20	0.58	0.49
**Berman** [16]	1966	United States	1958–1965	Neonates	22	22	7	6	1.00	0.86
**Justitz** [22]	1970	Switzerland	1955–1966	Children	138	8	102	11	0.06	0.11
**Wiebe** [28]	1972	United States	1965–1965	Children and adults	91	9	103	14	0.10	0.14
**Hodges** [37]	1975	United States	1949–1973	Not specified	198	52	151	38	0.26	0.25
**Kaiser** [38]	1975	Hungary	1964–1973	Children	22	6	12	4	0.27	0.33
**Dawson** [39]	1976	New Zealand	NA–NA	Children	33	6	20	1	0.18	0.05
**Goldacre** [40]	1976	United Kingdom	1969–1973	Neonates and children	434	59	304	35	0.14	0.12
**Yeung** [41]	1976	Hong Kong	NA–NA	Neonates	15	2	5	2	0.13	0.40
**Lang** [45]	1977	New Zealand	1971–1976	Children	121	13	78	9	0.11	0.12
**Bieler** [48]	1979	Switzerland	1967–1978	Neonates	14	8	8	5	0.57	0.63
**Chattopadhyay** [49]	1980	United Kingdom	1971–1978	Not specified	33	5	34	4	0.15	0.12
**Bohr** [59]	1983	Denmark	1966–1976	Not specified	486	54	389	39	0.11	0.10
**Salwen** [80]	1987	Sweden	1956–1975	Children	103	8	98	8	0.08	0.08
**Zaki** [98]	1990	Kuwait	1983–1988	Neonates	25	7	20	3	0.29	0.15
**Zaki** [99]	1990	Kuwait	1981–1987	Neonates and children	63	8	47	2	0.13	0.04
**Hanna** [102]	1991	Australia	1984–1988	Children	149	11	122	5	0.07	0.04
**Airede** [120]	1993	Nigeria	1988–1990	Neonates	23	3	13	9	0.23	0.77
**Pfister** [127]	1993	Germany	1984–1989	Adults	47	8	39	8	0.17	0.21
**Dagan** [132]	1994	Israel	1988–1991	Neonates and children	NA	9	NA	28	0.02	0.06
**Gedlu** [142]	1995	Ethiopia	1990–1994	Children	86	25	46	12	0.29	0.26
**Moller** [191]	2000	Denmark	1997–1999	Adults	8	1	9	0	0.13	0.00

**Johnson** [199]	2001	Nigeria	1992–1996	Neonates and children	39	18	23	12	0.46	0.52
**McMillan** [201]	2001	United States	1970–1998	Adults	152	36	142	41	0.24	0.29
**Molyneux** [215]	2002	Malawi	1997–2001	Children	337	104	261	96	0.31	0.37
**Kirimi** [221]	2003	Turkey	1999–2000	Children	29	3	19	3	0.10	0.16
**Rabbani** [222]	2003	Pakistan	1995–2002	Adults	146	30	48	13	0.21	0.27
**Celal** [223]	2004	Turkey	1996–2002	Adults	110	21	76	8	0.19	0.11
**Lucena** [236]	2005	Brazil	1997–1997	Children	NA	17	NA	13	0.20	0.16
**Faustini** [252]	2007	Italy	1996–2000	Not specified	287	56	238	42	0.20	0.18
**Johnson** [253]	2007	Nigeria	1998–2005	Neonates and children	39	18	23	12	0.46	0.52
**Sigauque** [267]	2008	Mozambique	1998–2003	Neonates and children	36	16	29	7	0.44	0.24
**Perez** [284]	2010	Cuba	1998–2007	Not specified	2783	1056	2015	1247	0.22	0.26
**Vibha** [287]	2010	India	2004–2008	Children and adults	322	22	58	12	0.07	0.21
**Namani** [296]	2012	Kosovo	1997–2002	Neonates and children	162	4	115	8	0.02	0.07
**Tarvij Eslami** [298]	2012	Iran	2005–2007	Neonates	33	6	27	4	0.18	0.15
**Banajeh** [310]	2014	Yemen	2000–2010	Children	1529	92	747	73	0.06	0.10
**Olson** [322]	2015	Guatemala	2000–2007	Neonates and children	468	96	332	96	0.20	0.29
**Park** [338]	2017	Republic of Korea	2009–2016	Not specified	49	8	31	5	0.16	0.16
**De Almeida** [346]	2019	Brazil	2006–2017	Not specified	23	4	26	5	0.17	0.19
**Huang** [356]	2020	Taiwan	2007–2013	Neonates	6	0	6	0	0.00	0.00

Supplementary Table 6. Study periods included in the meta-analyses by mean observation period interval and World Bank region.

Interval	World Bank Region	Study periods [k]	Episodes	Deaths
**Before 1961**	East Asia & Pacific	4	108	62
Before 1961	Europe & Central Asia	8	1,959	261
Before 1961	Middle East & North Africa	1	91	23
Before 1961	North America	15	1,938	601
Before 1961	Sub-Saharan Africa	4	413	87
1961-1970	East Asia & Pacific	1	5	2
1961-1970	Europe & Central Asia	5	571	69
1961-1970	Middle East & North Africa	2	247	80
1961-1970	North America	13	3,134	543
1961-1970	Sub-Saharan Africa	5	435	157
1971-1980	East Asia & Pacific	5	375	51
1971-1980	Europe & Central Asia	20	3,595	492
1971-1980	Latin America & Caribbean	3	3,973	1,312
1971-1980	Middle East & North Africa	3	832	245
1971-1980	North America	10	16,900	2,090
1971-1980	South Asia	2	325	112
1971-1980	Sub-Saharan Africa	4	1,010	320
1981-1990	East Asia & Pacific	24	2,825	462
1981-1990	Europe & Central Asia	26	8,777	819
1981-1990	Latin America & Caribbean	4	347	76
1981-1990	Middle East & North Africa	11	1,056	89
1981-1990	North America	9	4,170	401
1981-1990	South Asia	3	929	164
1981-1990	Sub-Saharan Africa	14	1,969	619
1991-2000	East Asia & Pacific	12	1,749	340
1991-2000	Europe & Central Asia	25	5,798	837
1991-2000	Latin America & Caribbean	10	2,041	300
1991-2000	Middle East & North Africa	7	755	61
1991-2000	North America	10	8,087	889
1991-2000	South Asia	10	1,024	188
1991-2000	Sub-Saharan Africa	33	8,278	1,943
2001-2010	East Asia & Pacific	9	2,310	267
2001-2010	Europe & Central Asia	23	9,183	969
2001-2010	Latin America & Caribbean	7	7,020	1,588
2001-2010	Middle East & North Africa	13	4,006	503
2001-2010	North America	2	5,937	583
2001-2010	South Asia	5	840	132
2001-2010	Sub-Saharan Africa	22	29,388	4,150
After 2010	East Asia & Pacific	7	658	41
After 2010	Europe & Central Asia	8	2,039	241
After 2010	Latin America & Caribbean	1	49	9

After 2010	Middle East & North Africa	3	138	19
After 2010	North America	6	5,768	403
After 2010	South Asia	5	366	54
After 2010	Sub-Saharan Africa	13	6,238	1,074

SUPPLEMENTARY FIGURES

Supplementary Figure 1. Imputed Human Development Index (HDI) values per country (colours) and year.

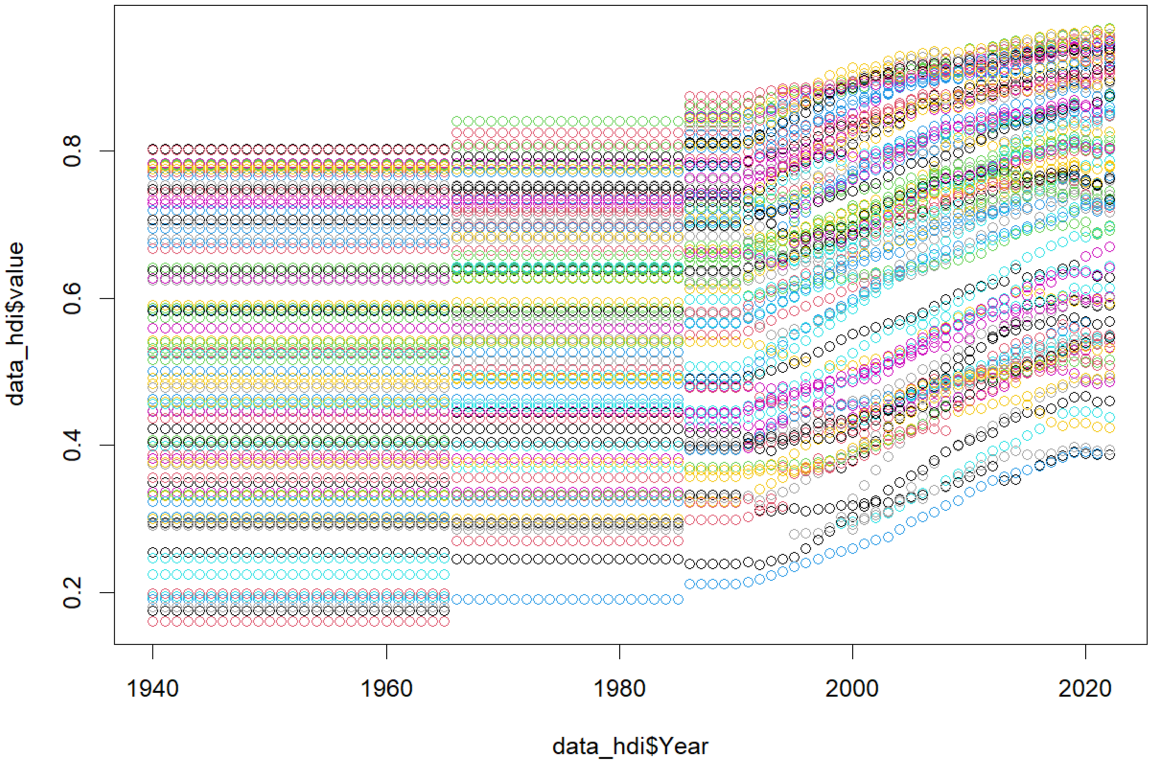

Supplementary Figure 2. Imputed Gender Inequality Index (GII) values per country (colours) and year.

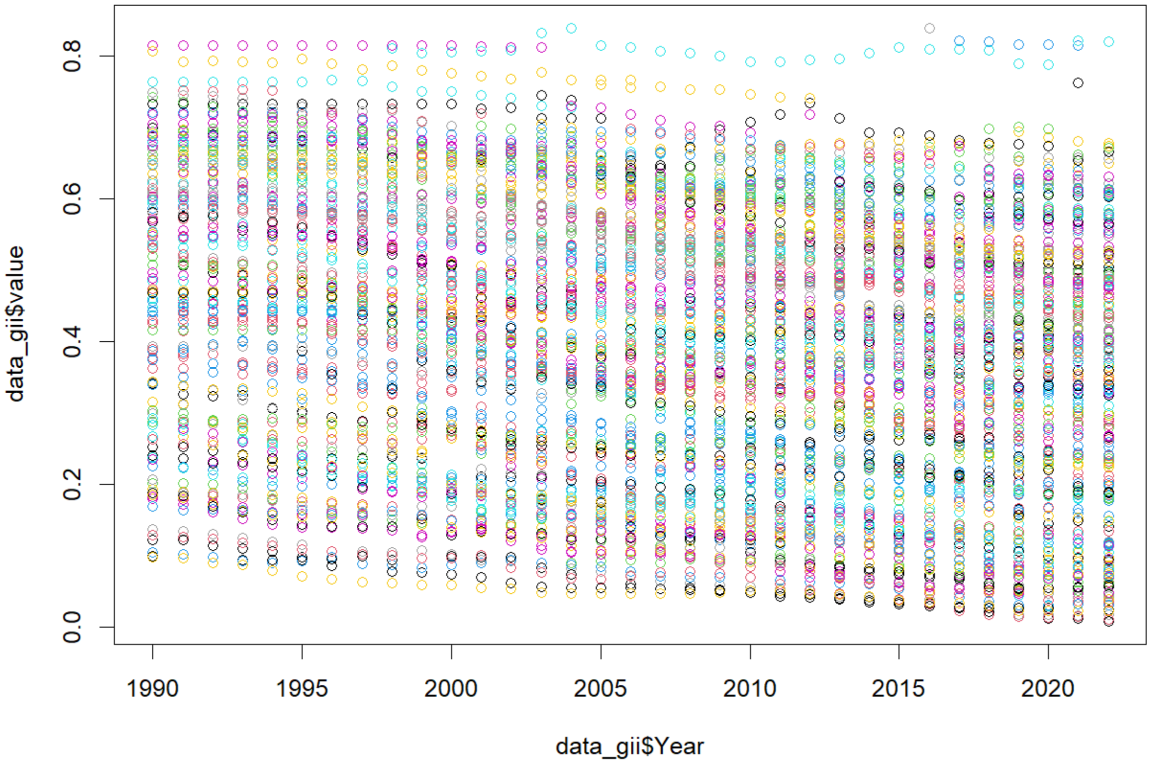

Supplementary Figure 3. Imputed Gini Index values per country (colours) and year.

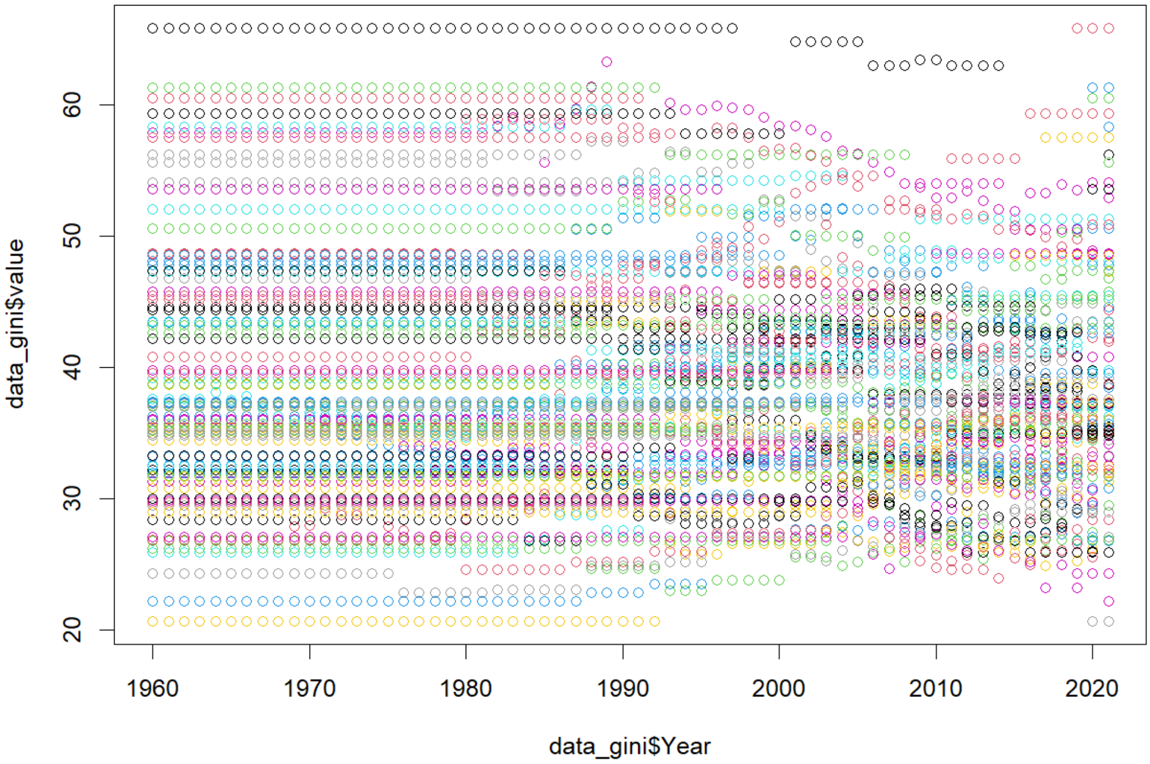

Supplementary Figure 4. Classification of regions according to World Bank.

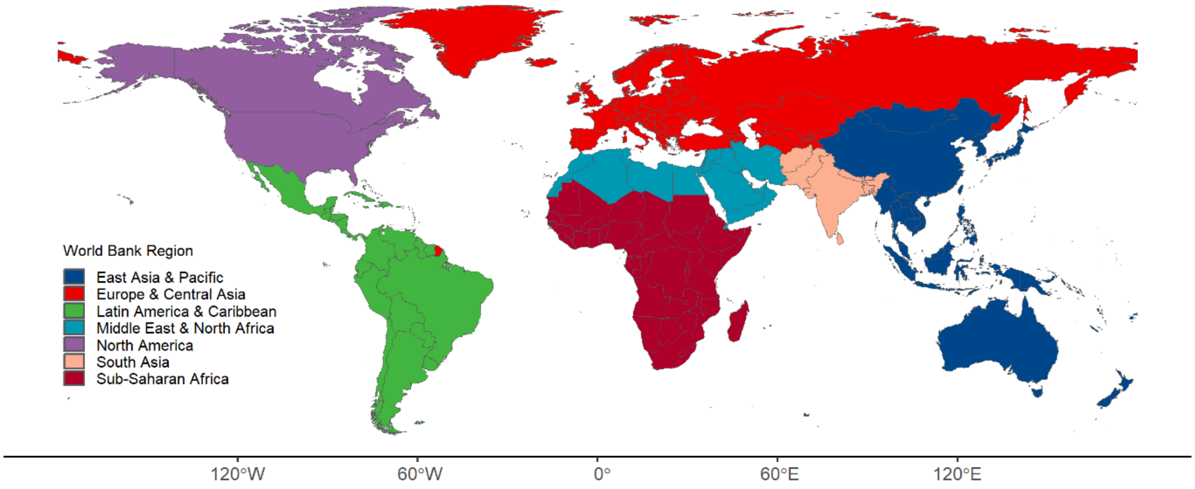

Supplementary Figure 5. Flow-chart of the study selection process.

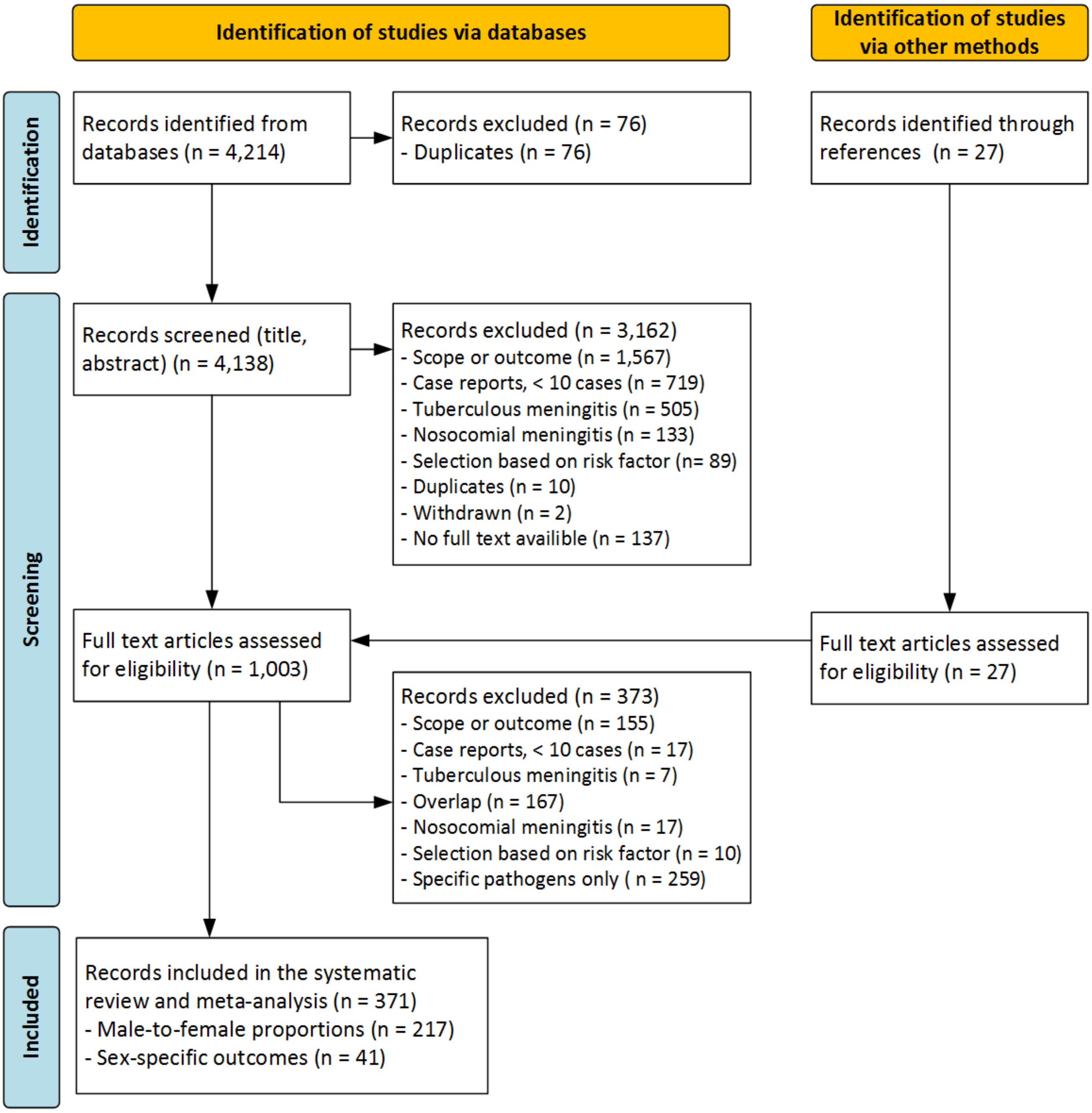

Supplementary Figure 6. Proportions of males in bacterial meningitis patients stratified by World Bank regions (Forest plots with individual studies suppressed) indicating the estimates of the subgroups and the overall pooled estimate.[†]

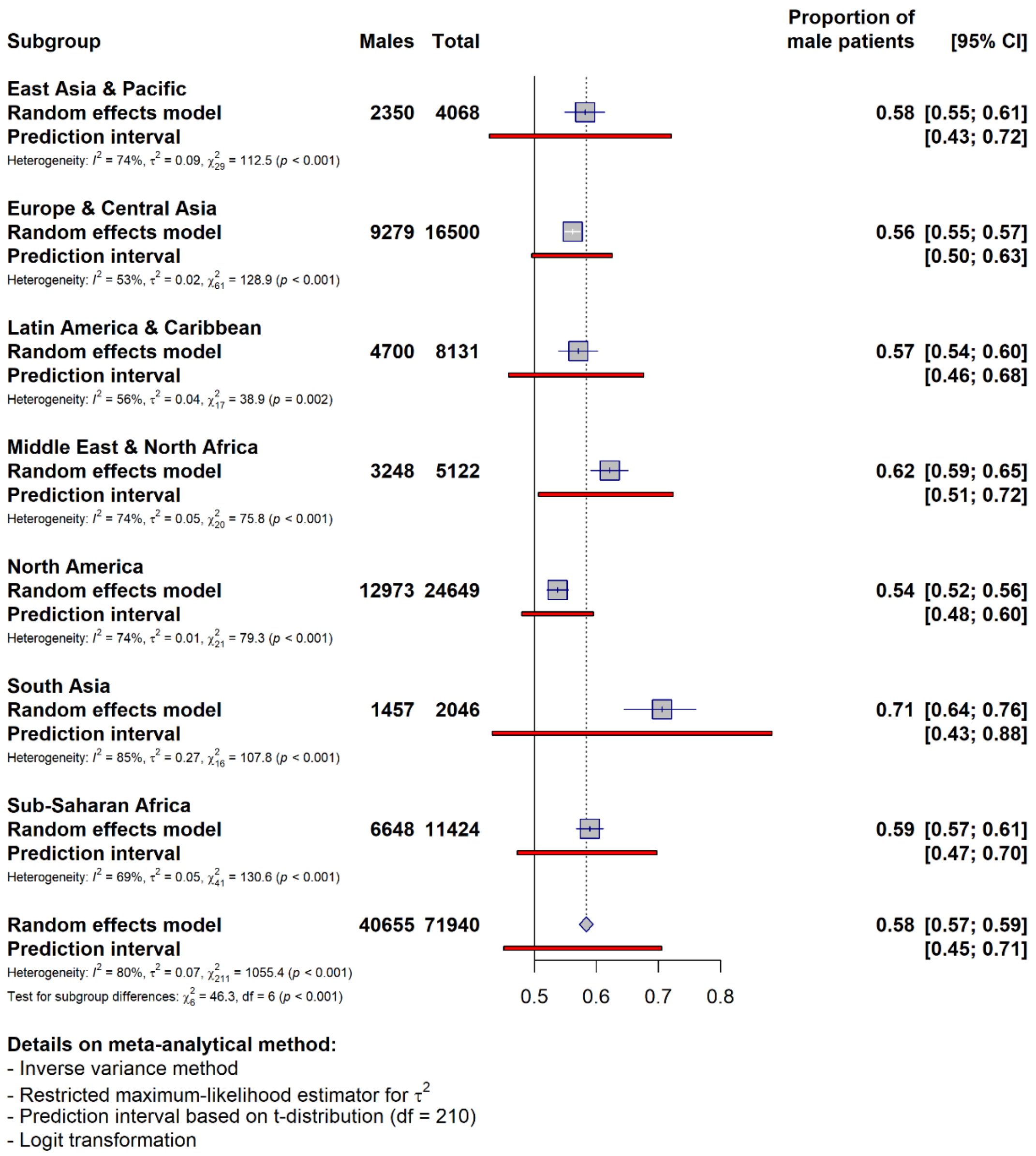

[†] Sub-Saharan Africa, k = 42; East Asia & Pacific, k = 30; Europe & Central Asia, k = 62; South Asia, k = 17; Middle East & North Africa, k = 21; Latin America & Caribbean, k = 18; North America, k = 22

Supplementary Figure 7. Funnel plot studies indicating male proportions in bacterial meningitis patients.

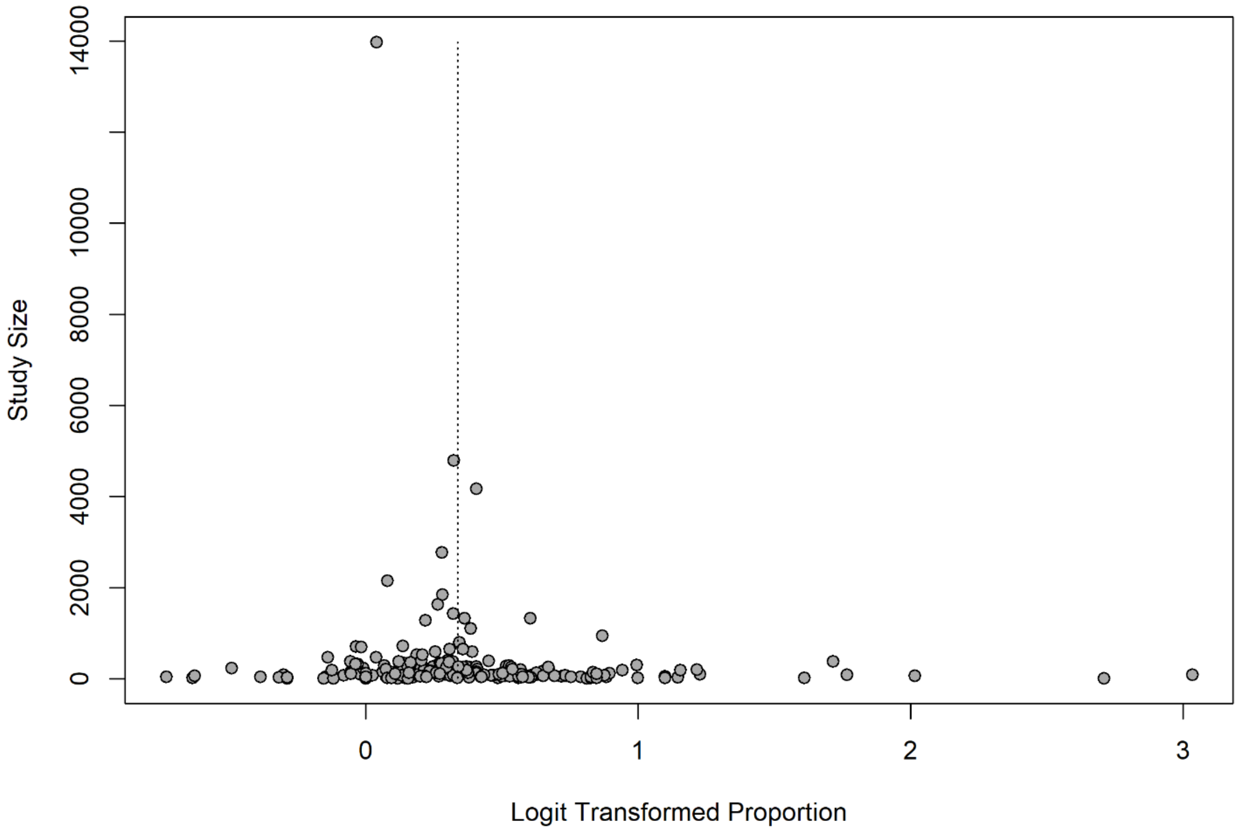

Supplementary Figure 8. Proportions of males in bacterial meningitis patients including mean observation periods after 2000 only (sensitivity analysis) stratified by World Bank regions (Forest plots with individual studies suppressed) indicating the estimates of the subgroups and the overall pooled estimate.‡

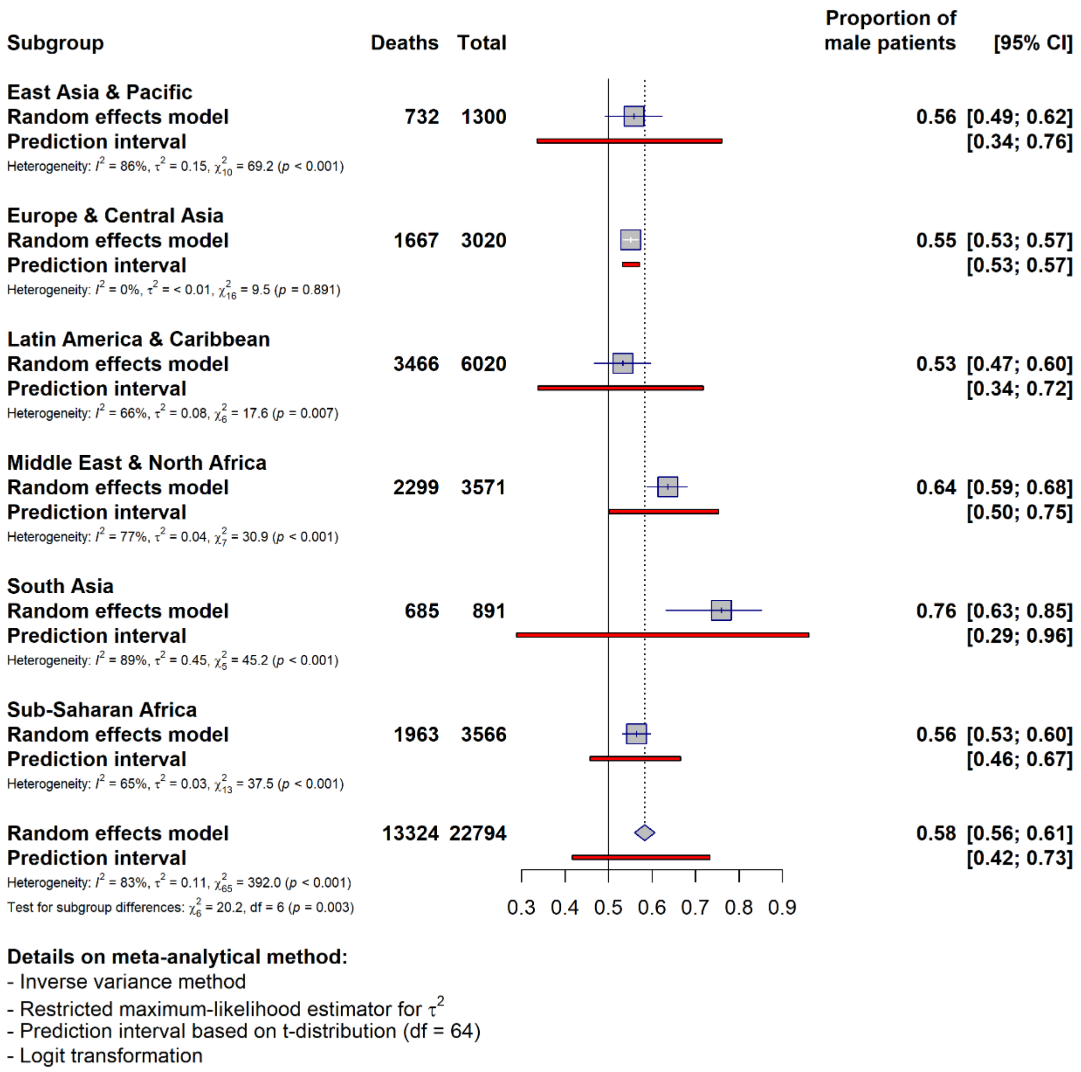

‡ Sub-Saharan Africa, k = 14; East Asia & Pacific, k = 11; Europe & Central Asia, k = 17; South Asia, k = 6; Middle East & North Africa, k = 8; Latin America & Caribbean, k = 7; North America, k = 3 (not shown).

Supplementary Figure 9. Proportions of males in bacterial meningitis patients using a meta–regression model with the mean observation period as predictor variable (P = 0.65).

(dashed lines, 95% CI; dotted lines, prediction interval)

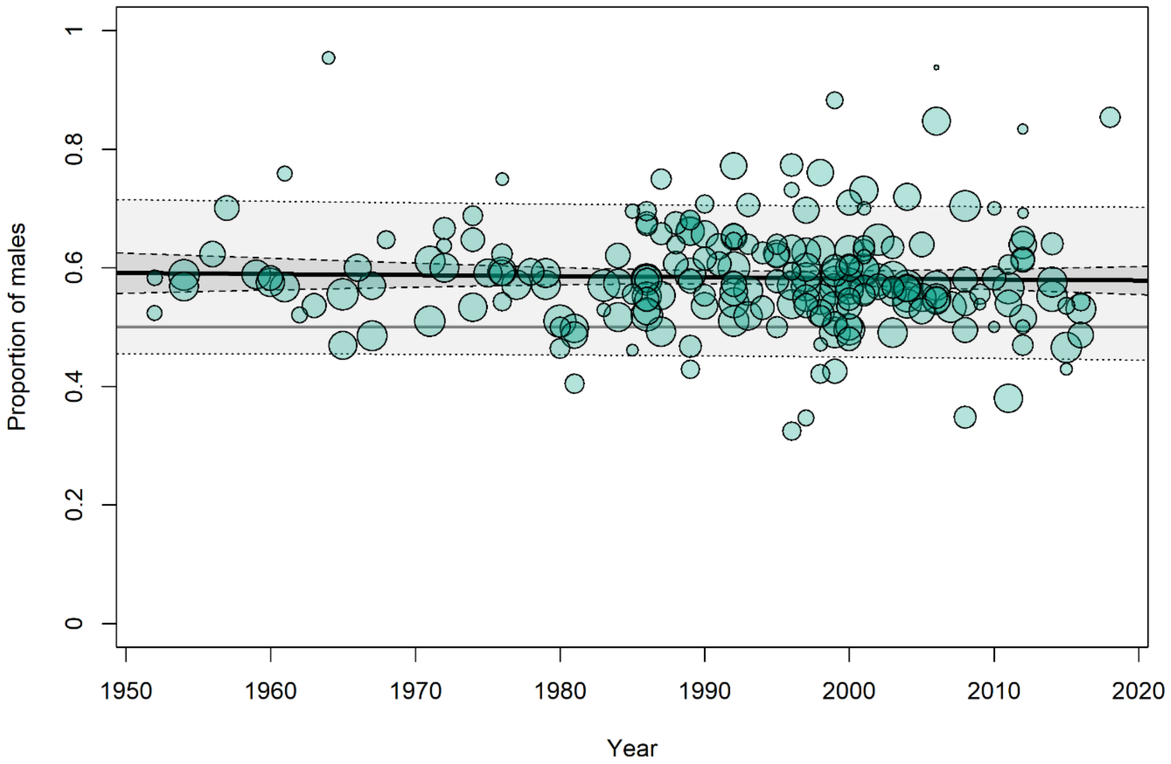

Supplementary Figure 10. Forest plot illustrating case fatality ratios (CFR) in female patients with World Bank regions as subgroups.

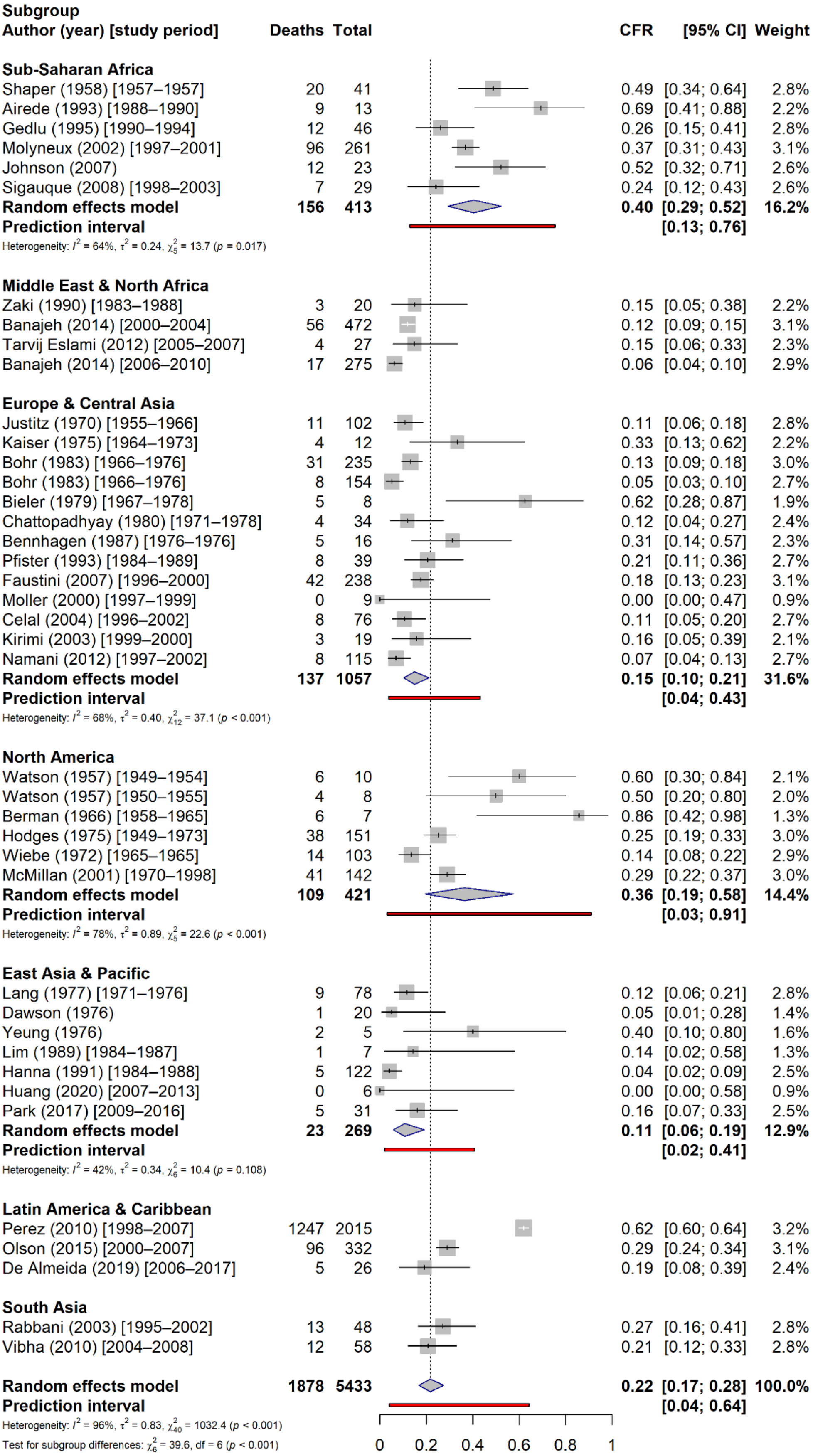

Supplementary Figure 11. Forest plot illustrating case fatality ratios (CFR) in male patients with World Bank regions as subgroups.

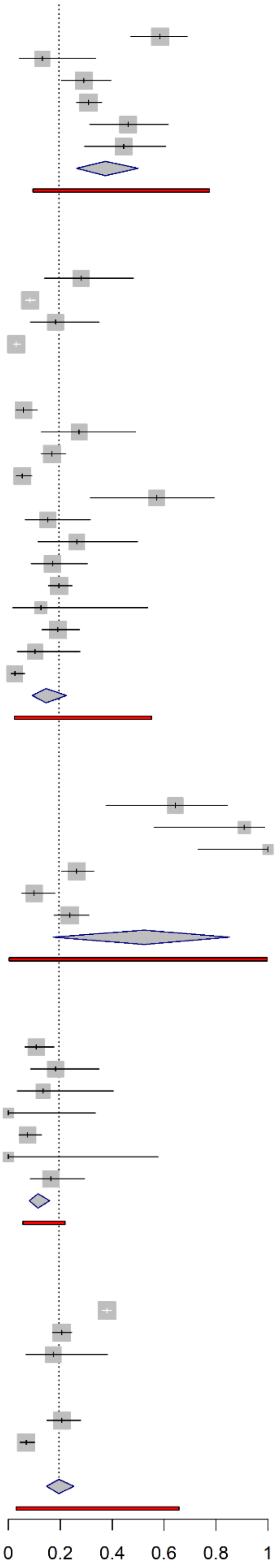

Subgroup / Author (year) [study period]	Deaths	Total	CFR	[95% CI]	Weight
**Sub-Saharan Africa**					
Shaper (1958) [1957–1957]	45	77	0.58	[0.47; 0.69]	2.8%
Airede (1993) [1988–1990]	3	23	0.13	[0.04; 0.34]	2.2%
Gedlu (1995) [1990–1994]	25	86	0.29	[0.20; 0.39]	2.8%
Molyneux (2002) [1997–2001]	104	337	0.31	[0.26; 0.36]	2.9%
Johnson (2007)	18	39	0.46	[0.31; 0.62]	2.7%
Sigauque (2008) [1998–2003]	16	36	0.44	[0.29; 0.61]	2.7%
**Random effects model**	**211**	**598**	**0.37**	**[0.26; 0.50]**	**16.1%**
**Prediction interval**				**[0.09; 0.77]**	
Heterogeneity: $I^2 = 83\%$, $\tau^2 = 0.33$, $\chi^2_5 = 28.7$ ($p < 0.001$)					
**Middle East & North Africa**					
Zaki (1990) [1983–1988]	7	25	0.28	[0.14; 0.48]	2.5%
Banajeh (2014) [2000–2004]	72	864	0.08	[0.07; 0.10]	2.9%
Tarvij Eslami (2012) [2005–2007]	6	33	0.18	[0.08; 0.35]	2.5%
Banajeh (2014) [2006–2010]	20	665	0.03	[0.02; 0.05]	2.8%
**Europe & Central Asia**					
Justitz (1970) [1955–1966]	8	138	0.06	[0.03; 0.11]	2.6%
Kaiser (1975) [1964–1973]	6	22	0.27	[0.13; 0.49]	2.4%
Bohr (1983) [1966–1976]	41	244	0.17	[0.13; 0.22]	2.9%
Bohr (1983) [1966–1976]	13	242	0.05	[0.03; 0.09]	2.7%
Bieler (1979) [1967–1978]	8	14	0.57	[0.32; 0.79]	2.3%
Chattopadhyay (1980) [1971–1978]	5	33	0.15	[0.06; 0.32]	2.4%
Bennhagen (1987) [1976–1976]	5	19	0.26	[0.11; 0.50]	2.3%
Pfister (1993) [1984–1989]	8	47	0.17	[0.09; 0.31]	2.6%
Faustini (2007) [1996–2000]	56	287	0.20	[0.15; 0.25]	2.9%
Moller (2000) [1997–1999]	1	8	0.12	[0.02; 0.54]	1.4%
Celal (2004) [1996–2002]	21	110	0.19	[0.13; 0.28]	2.8%
Kirimi (2003) [1999–2000]	3	29	0.10	[0.03; 0.28]	2.2%
Namani (2012) [1997–2002]	4	162	0.02	[0.01; 0.06]	2.4%
**Random effects model**	**179**	**1355**	**0.15**	**[0.09; 0.22]**	**32.0%**
**Prediction interval**				**[0.02; 0.55]**	
Heterogeneity: $I^2 = 81\%$, $\tau^2 = 0.73$, $\chi^2_{12} = 64.2$ ($p < 0.001$)					
**North America**					
Watson (1957) [1949–1954]	9	14	0.64	[0.38; 0.84]	2.3%
Watson (1957) [1950–1955]	10	11	0.91	[0.56; 0.99]	1.4%
Berman (1966) [1958–1965]	22	22	1.00	[0.73; 1.00]	1.0%
Hodges (1975) [1949–1973]	52	198	0.26	[0.21; 0.33]	2.9%
Wiebe (1972) [1965–1965]	9	91	0.10	[0.05; 0.18]	2.6%
McMillan (2001) [1970–1998]	36	152	0.24	[0.18; 0.31]	2.9%
**Random effects model**	**138**	**488**	**0.52**	**[0.17; 0.85]**	**13.1%**
**Prediction interval**				**[0.00; 1.00]**	
Heterogeneity: $I^2 = 88\%$, $\tau^2 = 3.81$, $\chi^2_5 = 41.7$ ($p < 0.001$)					
**East Asia & Pacific**					
Lang (1977) [1971–1976]	13	121	0.11	[0.06; 0.18]	2.7%
Dawson (1976)	6	33	0.18	[0.08; 0.35]	2.5%
Yeung (1976)	2	15	0.13	[0.03; 0.41]	1.9%
Lim (1989) [1984–1987]	0	16	0.00	[0.00; 0.34]	1.0%
Hanna (1991) [1984–1988]	11	149	0.07	[0.04; 0.13]	2.7%
Huang (2020) [2007–2013]	0	6	0.00	[0.00; 0.58]	1.0%
Park (2017) [2009–2016]	8	49	0.16	[0.08; 0.29]	2.6%
**Random effects model**	**40**	**389**	**0.11**	**[0.08; 0.16]**	**14.3%**
**Prediction interval**				**[0.06; 0.22]**	
Heterogeneity: $I^2 = 3\%$, $\tau^2 = 0.05$, $\chi^2_6 = 6.2$ ($p = 0.402$)					
**Latin America & Caribbean**					
Perez (2010) [1998–2007]	1056	2783	0.38	[0.36; 0.40]	3.0%
Olson (2015) [2000–2007]	96	468	0.21	[0.17; 0.24]	2.9%
De Almeida (2019) [2006–2017]	4	23	0.17	[0.07; 0.38]	2.3%
**South Asia**					
Rabbani (2003) [1995–2002]	30	146	0.21	[0.15; 0.28]	2.8%
Vibha (2010) [2004–2008]	22	322	0.07	[0.05; 0.10]	2.8%
**Random effects model**	**1881**	**8159**	**0.19**	**[0.15; 0.25]**	**100.0%**
**Prediction interval**				**[0.03; 0.66]**	
Heterogeneity: $I^2 = 95\%$, $\tau^2 = 1.02$, $\chi^2_{40} = 758.0$ ($p < 0.001$)					
Test for subgroup differences: $\chi^2_6 = 29.6$, df = 6 ($p < 0.001$)					

Supplementary Figure 12. Forest plot illustrating case fatality ratios (CFR) male-to-female ratio with World Bank regions as subgroups.

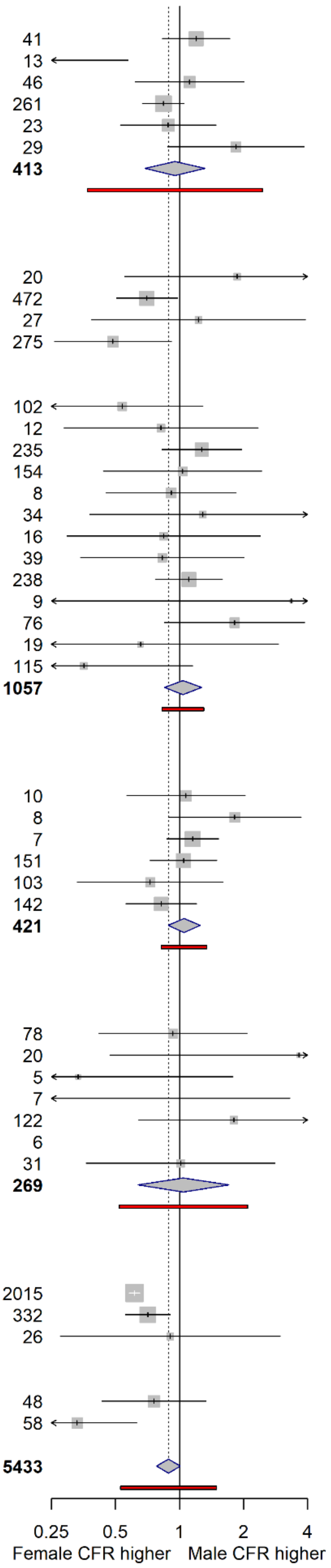

Subgroup / Author (year) [study period]	Males Deaths	Males Total	Females Deaths	Females Total	CFR ratio	[95% CI]	Weight
**Sub-Saharan Africa**							
Shaper (1958) [1957–1957]	45	77	20	41	1.20	[0.83; 1.73]	4.6%
Airede (1993) [1988–1990]	3	23	9	13	0.19	[0.06; 0.57]	1.2%
Gedlu (1995) [1990–1994]	25	86	12	46	1.11	[0.62; 2.01]	2.9%
Molyneux (2002) [1997–2001]	104	337	96	261	0.84	[0.67; 1.05]	5.9%
Johnson (2007)	18	39	12	23	0.88	[0.53; 1.48]	3.4%
Sigauque (2008) [1998–2003]	16	36	7	29	1.84	[0.88; 3.86]	2.2%
**Random effects model**		**598**		**413**	**0.95**	**[0.69; 1.32]**	**20.1%**
**Prediction interval**						**[0.37; 2.45]**	
Heterogeneity: $I^2 = 65\%$, $\tau^2 = 0.09$, $\chi^2_5 = 14.2$ ($p = 0.01$)							
**Middle East & North Africa**							
Zaki (1990) [1983–1988]	7	25	3	20	1.87	[0.55; 6.31]	1.0%
Banajeh (2014) [2000–2004]	72	864	56	472	0.70	[0.50; 0.98]	4.9%
Tarvij Eslami (2012) [2005–2007]	6	33	4	27	1.23	[0.39; 3.91]	1.1%
Banajeh (2014) [2006–2010]	20	665	17	275	0.49	[0.26; 0.91]	2.7%
**Europe & Central Asia**							
Justitz (1970) [1955–1966]	8	138	11	102	0.54	[0.22; 1.29]	1.7%
Kaiser (1975) [1964–1973]	6	22	4	12	0.82	[0.29; 2.34]	1.3%
Bohr (1983) [1966–1976]	41	244	31	235	1.27	[0.83; 1.96]	4.0%
Bohr (1983) [1966–1976]	13	242	8	154	1.03	[0.44; 2.44]	1.8%
Bieler (1979) [1967–1978]	8	14	5	8	0.91	[0.45; 1.85]	2.3%
Chattopadhyay (1980) [1971–1978]	5	33	4	34	1.29	[0.38; 4.38]	1.0%
Bennhagen (1987) [1976–1976]	5	19	5	16	0.84	[0.30; 2.40]	1.3%
Pfister (1993) [1984–1989]	8	47	8	39	0.83	[0.34; 2.01]	1.7%
Faustini (2007) [1996–2000]	56	287	42	238	1.11	[0.77; 1.59]	4.6%
Moller (2000) [1997–1999]	1	8	0	9	3.35	[0.16; 71.79]	0.2%
Celal (2004) [1996–2002]	21	110	8	76	1.81	[0.85; 3.88]	2.1%
Kirimi (2003) [1999–2000]	3	29	3	19	0.66	[0.15; 2.91]	0.7%
Namani (2012) [1997–2002]	4	162	8	115	0.35	[0.11; 1.15]	1.1%
**Random effects model**		**1355**		**1057**	**1.04**	**[0.85; 1.27]**	**23.7%**
**Prediction interval**						**[0.83; 1.30]**	
Heterogeneity: $I^2 = 0\%$, $\tau^2 = 0$, $\chi^2_{12} = 10.2$ ($p = 0.60$)							
**North America**							
Watson (1957) [1949–1954]	9	14	6	10	1.07	[0.57; 2.03]	2.6%
Watson (1957) [1950–1955]	10	11	4	8	1.82	[0.89; 3.73]	2.3%
Berman (1966) [1958–1965]	22	22	6	7	1.15	[0.87; 1.53]	5.4%
Hodges (1975) [1949–1973]	52	198	38	151	1.04	[0.73; 1.50]	4.6%
Wiebe (1972) [1965–1965]	9	91	14	103	0.73	[0.33; 1.60]	2.0%
McMillan (2001) [1970–1998]	36	152	41	142	0.82	[0.56; 1.21]	4.4%
**Random effects model**		**488**		**421**	**1.05**	**[0.88; 1.25]**	**21.3%**
**Prediction interval**						**[0.82; 1.34]**	
Heterogeneity: $I^2 = 2\%$, $\tau^2 = < 0.01$, $\chi^2_5 = 5.1$ ($p = 0.40$)							
**East Asia & Pacific**							
Lang (1977) [1971–1976]	13	121	9	78	0.93	[0.42; 2.07]	1.9%
Dawson (1976)	6	33	1	20	3.64	[0.47; 28.05]	0.4%
Yeung (1976)	2	15	2	5	0.33	[0.06; 1.79]	0.6%
Lim (1989) [1984–1987]	0	16	1	7	0.15	[0.01; 3.30]	0.2%
Hanna (1991) [1984–1988]	11	149	5	122	1.80	[0.64; 5.04]	1.3%
Huang (2020) [2007–2013]	0	6	0	6			0.0%
Park (2017) [2009–2016]	8	49	5	31	1.01	[0.36; 2.82]	1.3%
**Random effects model**		**389**		**269**	**1.04**	**[0.64; 1.70]**	**5.7%**
**Prediction interval**						**[0.52; 2.09]**	
Heterogeneity: $I^2 = 15\%$, $\tau^2 = < 0.01$, $\chi^2_5 = 5.9$ ($p = 0.32$)							
**Latin America & Caribbean**							
Perez (2010) [1998–2007]	1056	2783	1247	2015	0.61	[0.58; 0.65]	7.1%
Olson (2015) [2000–2007]	96	468	96	332	0.71	[0.55; 0.91]	5.7%
De Almeida (2019) [2006–2017]	4	23	5	26	0.90	[0.28; 2.97]	1.0%
**South Asia**							
Rabbani (2003) [1995–2002]	30	146	13	48	0.76	[0.43; 1.33]	3.1%
Vibha (2010) [2004–2008]	22	322	12	58	0.33	[0.17; 0.63]	2.6%
**Random effects model**		**8159**		**5433**	**0.89**	**[0.78; 1.01]**	**100.0%**
**Prediction interval**						**[0.53; 1.49]**	
Heterogeneity: $I^2 = 66\%$, $\tau^2 = 0.06$, $\chi^2_{39} = 114.8$ ($p < 0.01$)							
Test for subgroup differences: $\chi^2_6 = 37.3$, df = 6 ($p < 0.01$)							

Supplementary Figure 13. Funnel plot of studies indicating sex-specific case fatality ratios.

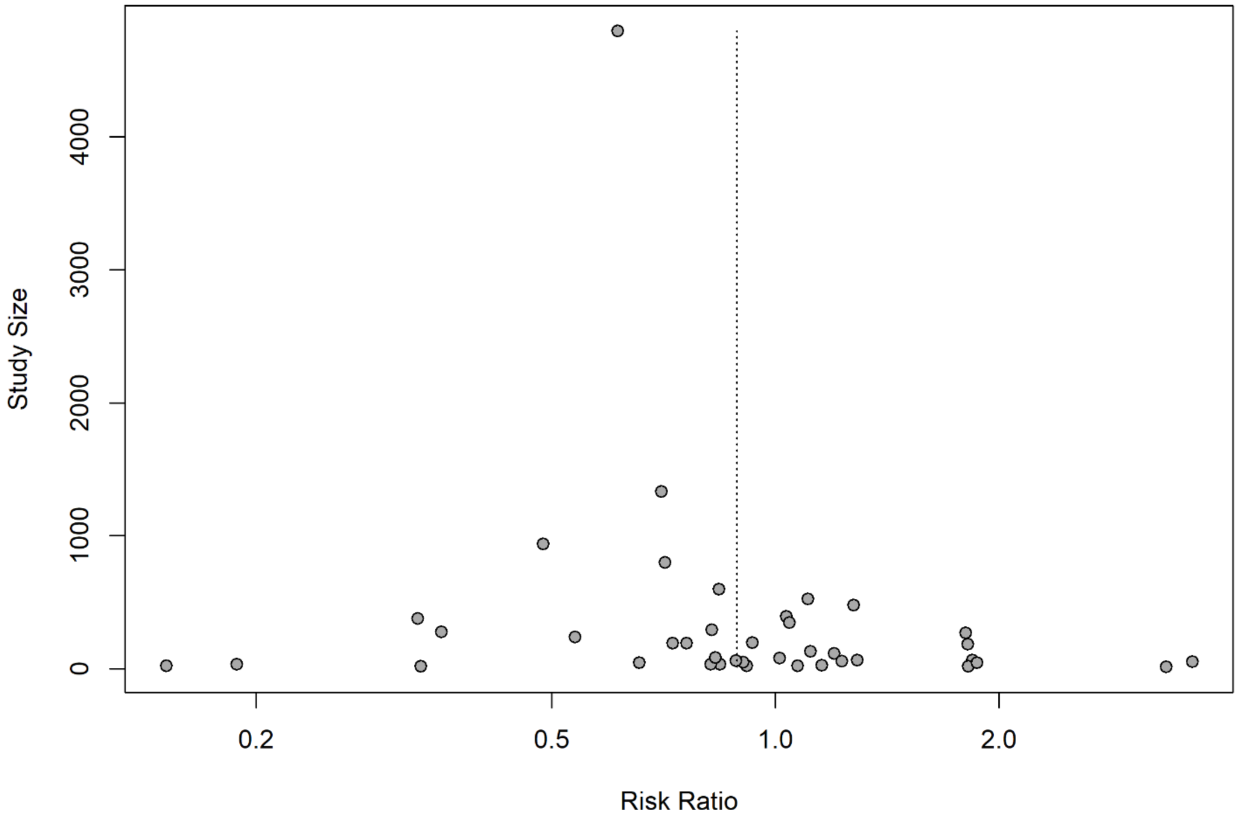

Supplementary Figure 14. Forest plot illustrating male-to-female case fatality ratio (CFR) ratios with age groups as subgroups.

Subgroup<br>Author (year) [study period]	Experimental<br>Deaths	 Total	Control<br>Deaths	 Total	CFR ratio	[95% CI]	Weight
**Neonates**							
Watson (1957) [1949–1954]	9	14	6	10	1.07	[0.57; 2.03]	2.6%
Watson (1957) [1950–1955]	10	11	4	8	1.82	[0.89; 3.73]	2.3%
Berman (1966) [1958–1965]	22	22	6	7	1.15	[0.87; 1.53]	5.4%
Bieler (1979) [1967–1978]	8	14	5	8	0.91	[0.45; 1.85]	2.3%
Bennhagen (1987) [1976–1976]	5	19	5	16	0.84	[0.30; 2.40]	1.3%
Yeung (1976)	2	15	2	5	0.33	[0.06; 1.79]	0.6%
Zaki (1990) [1983–1988]	7	25	3	20	1.87	[0.55; 6.31]	1.0%
Airede (1993) [1988–1990]	3	23	9	13	0.19	[0.06; 0.57]	1.2%
Tarvij Eslami (2012) [2005–2007]	6	33	4	27	1.23	[0.39; 3.91]	1.1%
Huang (2020) [2007–2013]	0	6	0	6			0.0%
**Random effects model**	**72**	**182**	**44**	**120**	**0.99**	**[0.70; 1.40]**	**17.7%**
**Prediction interval**						**[0.41; 2.38]**	
Heterogeneity: $I^2 = 46\%$, $\tau^2 = 0.11$, $\chi^2_8 = 14.8$ ($p = 0.063$)							
**Neonates and children**							
Bohr (1983) [1966–1976]	13	242	8	154	1.03	[0.44; 2.44]	1.8%
Namani (2012) [1997–2002]	4	162	8	115	0.35	[0.11; 1.15]	1.1%
Johnson (2007)	18	39	12	23	0.88	[0.53; 1.48]	3.4%
Sigauque (2008) [1998–2003]	16	36	7	29	1.84	[0.88; 3.86]	2.2%
Olson (2015) [2000–2007]	96	468	96	332	0.71	[0.55; 0.91]	5.7%
**Random effects model**	**147**	**947**	**131**	**653**	**0.87**	**[0.60; 1.26]**	**14.1%**
**Prediction interval**						**[0.30; 2.53]**	
Heterogeneity: $I^2 = 51\%$, $\tau^2 = 0.08$, $\chi^2_4 = 8.1$ ($p = 0.089$)							
**Children**							
Justitz (1970) [1955–1966]	8	138	11	102	0.54	[0.22; 1.29]	1.7%
Kaiser (1975) [1964–1973]	6	22	4	12	0.82	[0.29; 2.34]	1.3%
Lang (1977) [1971–1976]	13	121	9	78	0.93	[0.42; 2.07]	1.9%
Dawson (1976)	6	33	1	20	3.64	[0.47; 28.05]	0.4%
Lim (1989) [1984–1987]	0	16	1	7	0.15	[0.01; 3.30]	0.2%
Hanna (1991) [1984–1988]	11	149	5	122	1.80	[0.64; 5.04]	1.3%
Gedlu (1995) [1990–1994]	25	86	12	46	1.11	[0.62; 2.01]	2.9%
Molyneux (2002) [1997–2001]	104	337	96	261	0.84	[0.67; 1.05]	5.9%
Kirimi (2003) [1999–2000]	3	29	3	19	0.66	[0.15; 2.91]	0.7%
Banajeh (2014) [2000–2004]	72	864	56	472	0.70	[0.50; 0.98]	4.9%
Banajeh (2014) [2006–2010]	20	665	17	275	0.49	[0.26; 0.91]	2.7%
**Random effects model**	**268**	**2460**	**215**	**1414**	**0.80**	**[0.68; 0.94]**	**23.9%**
**Prediction interval**						**[0.67; 0.96]**	
Heterogeneity: $I^2 = 9\%$, $\tau^2 = < 0.01$, $\chi^2_{10} = 11$ ($p = 0.358$)							
**Children and adults**							
Wiebe (1972) [1965–1965]	9	91	14	103	0.73	[0.33; 1.60]	2.0%
Vibha (2010) [2004–2008]	22	322	12	58	0.33	[0.17; 0.63]	2.6%
**Adults**							
Bohr (1983) [1966–1976]	41	244	31	235	1.27	[0.83; 1.96]	4.0%
McMillan (2001) [1970–1998]	36	152	41	142	0.82	[0.56; 1.21]	4.4%
Pfister (1993) [1984–1989]	8	47	8	39	0.83	[0.34; 2.01]	1.7%
Moller (2000) [1997–1999]	1	8	0	9	3.35	[0.16; 71.79]	0.2%
Rabbani (2003) [1995–2002]	30	146	13	48	0.76	[0.43; 1.33]	3.1%
Celal (2004) [1996–2002]	21	110	8	76	1.81	[0.85; 3.88]	2.1%
**Random effects model**	**137**	**707**	**101**	**549**	**1.02**	**[0.76; 1.36]**	**15.5%**
**Prediction interval**						**[0.54; 1.93]**	
Heterogeneity: $I^2 = 20\%$, $\tau^2 = 0.03$, $\chi^2_5 = 6.3$ ($p = 0.280$)							
**Not specified**							
Shaper (1958) [1957–1957]	45	77	20	41	1.20	[0.83; 1.73]	4.6%
Hodges (1975) [1949–1973]	52	198	38	151	1.04	[0.73; 1.50]	4.6%
Chattopadhyay (1980) [1971–1978]	5	33	4	34	1.29	[0.38; 4.38]	1.0%
Faustini (2007) [1996–2000]	56	287	42	238	1.11	[0.77; 1.59]	4.6%
Perez (2010) [1998–2007]	1056	2783	1247	2015	0.61	[0.58; 0.65]	7.1%
De Almeida (2019) [2006–2017]	4	23	5	26	0.90	[0.28; 2.97]	1.0%
Park (2017) [2009–2016]	8	49	5	31	1.01	[0.36; 2.82]	1.3%
**Random effects model**	**1226**	**3450**	**1361**	**2536**	**0.94**	**[0.70; 1.25]**	**24.3%**
**Prediction interval**						**[0.42; 2.10]**	
Heterogeneity: $I^2 = 81\%$, $\tau^2 = 0.08$, $\chi^2_6 = 31.5$ ($p < 0.001$)							
**Random effects model**	**1881**	**8159**	**1878**	**5433**	**0.89**	**[0.78; 1.01]**	**100.0%**
**Prediction interval**						**[0.53; 1.49]**	
Heterogeneity: $I^2 = 66\%$, $\tau^2 = 0.06$, $\chi^2_{39} = 114.8$ ($p < 0.001$)							
Test for subgroup differences: $\chi^2_5 = 5.3$, df = 5 ($p = 0.380$)							

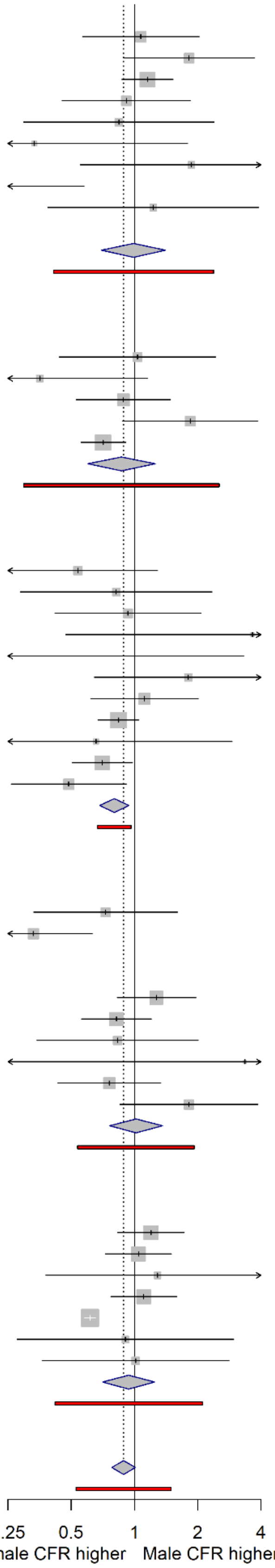

Supplementary Figure 15. Meta-analysis of case fatality ratios (CFR) with regions as subgroups (all studies with mean observation period after 1940).[§]

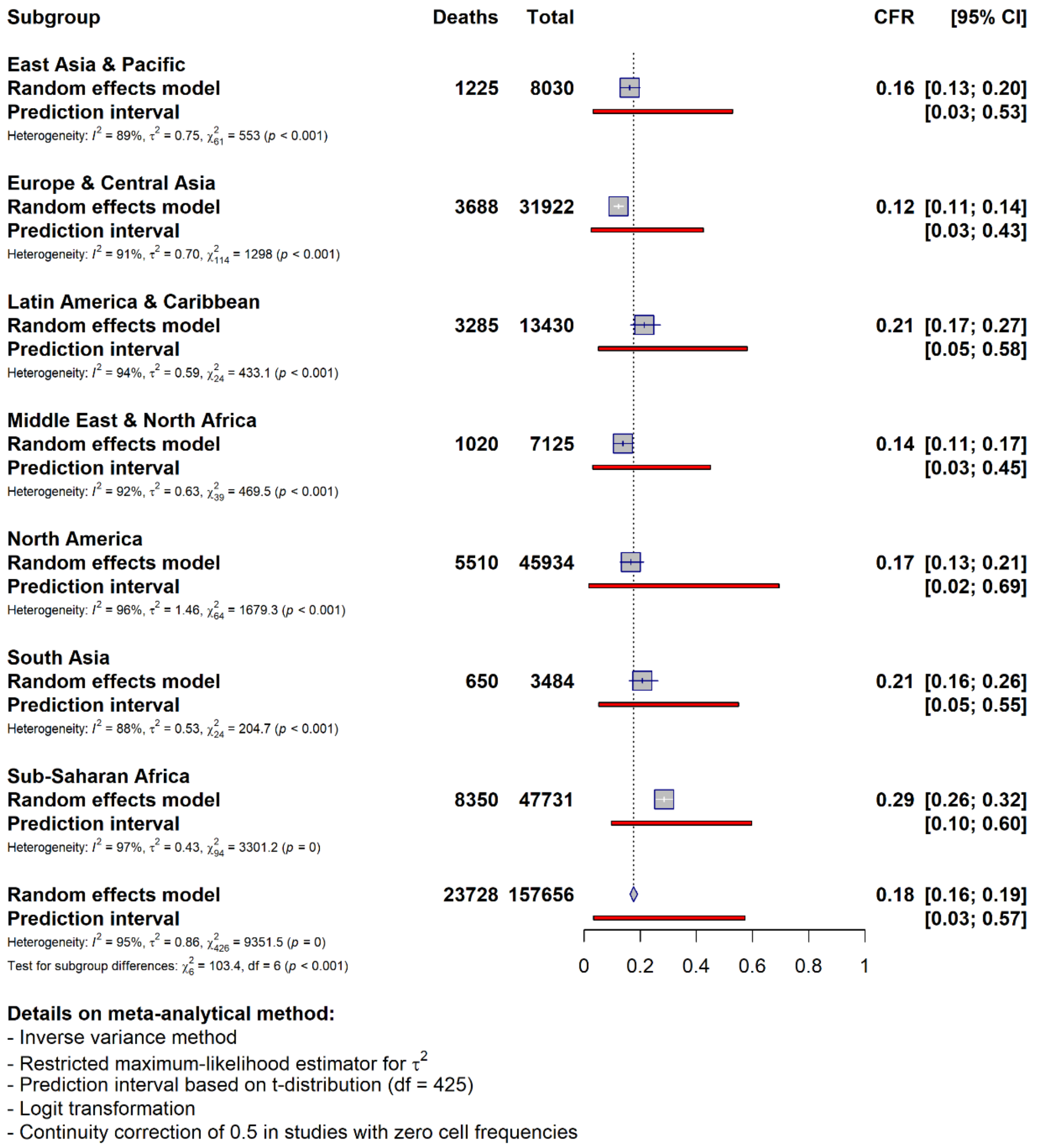

[§] Sub-Saharan Africa, k = 95; East Asia & Pacific, k = 60; Middle East & North Africa, k = 40; Europe & Central Asia, k = 115; South Asia, k = 25; Latin America & Caribbean, k = 24; North America, k = 65

Supplementary Figure 16. Meta-analysis of case fatality ratios (CFR) with regions as subgroups (all studies with mean observation period after 2000 only.[**]

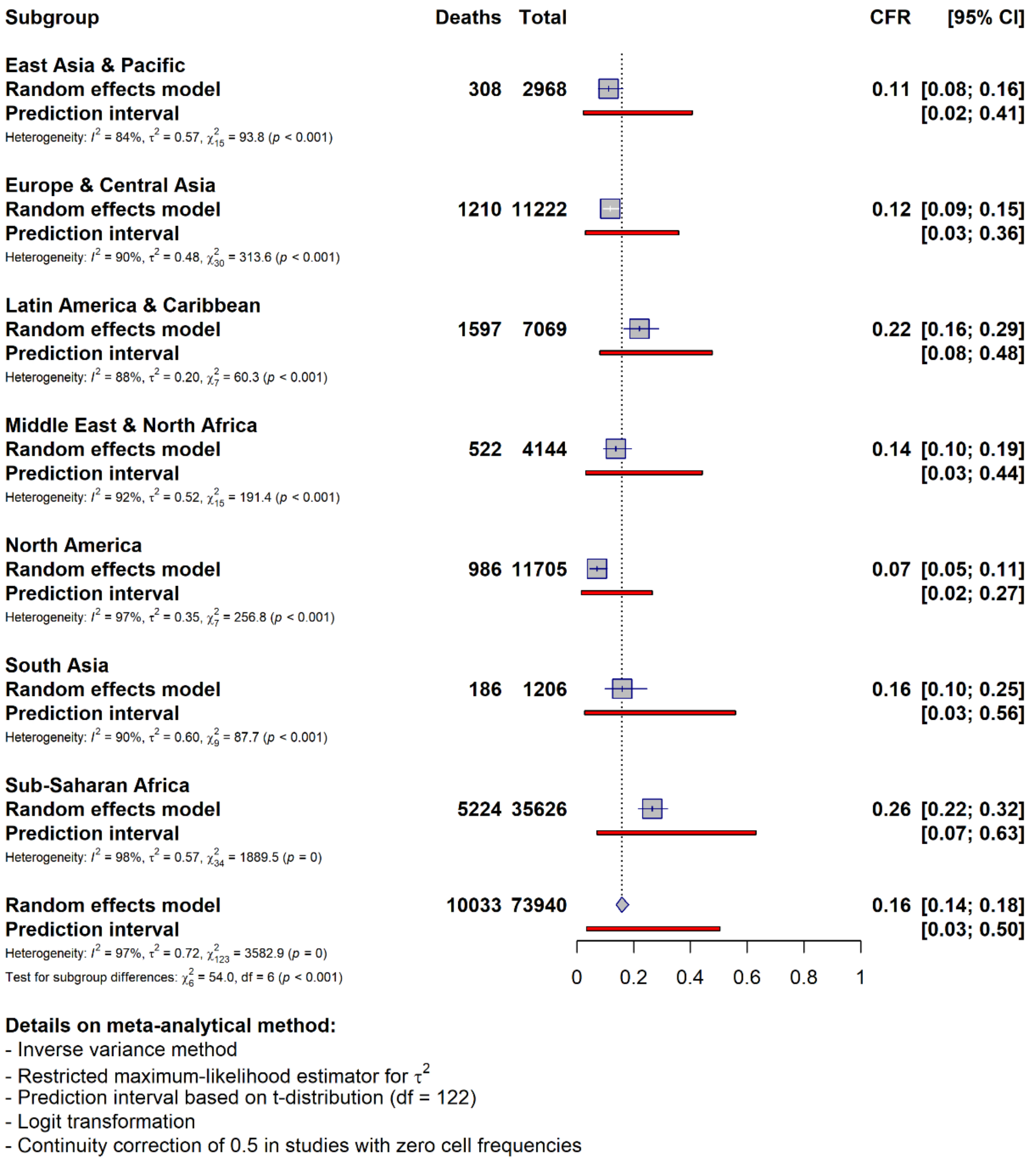

[**] Sub-Saharan Africa, k = 35; Middle East & North Africa, k = 17; Europe & Central Asia, k = 31; East Asia & Pacific, k = 16; South Asia, k = 10; Latin America & Caribbean, k = 8; North America, k = 8

Supplementary Figure 17. Case fatality ratio in bacterial meningitis with the Human Development Index as predictor, stratified by the age group (**A**, adults, ≥ 16 years old; **B**, children 2 months to 16 years old; **C**, neonates, < 2 months old).

(Dashed lines, 95% CI; dotted lines, prediction interval)

A

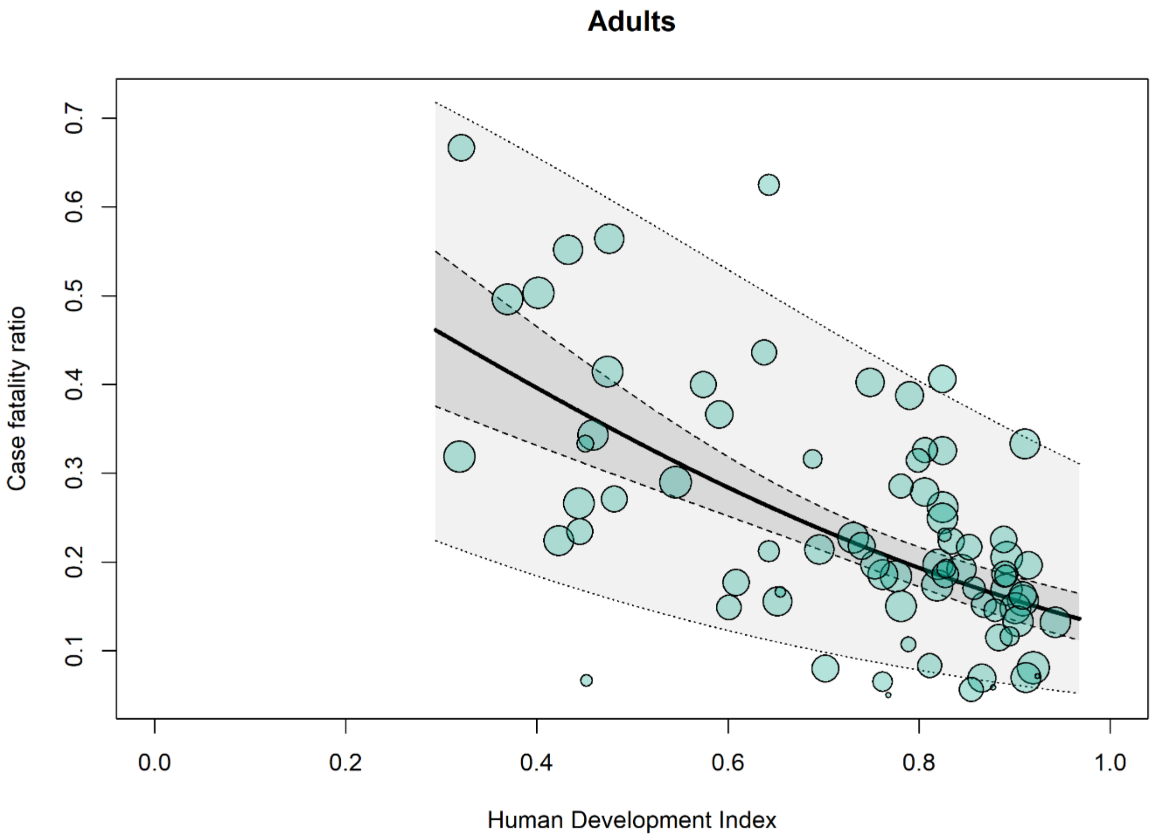

B

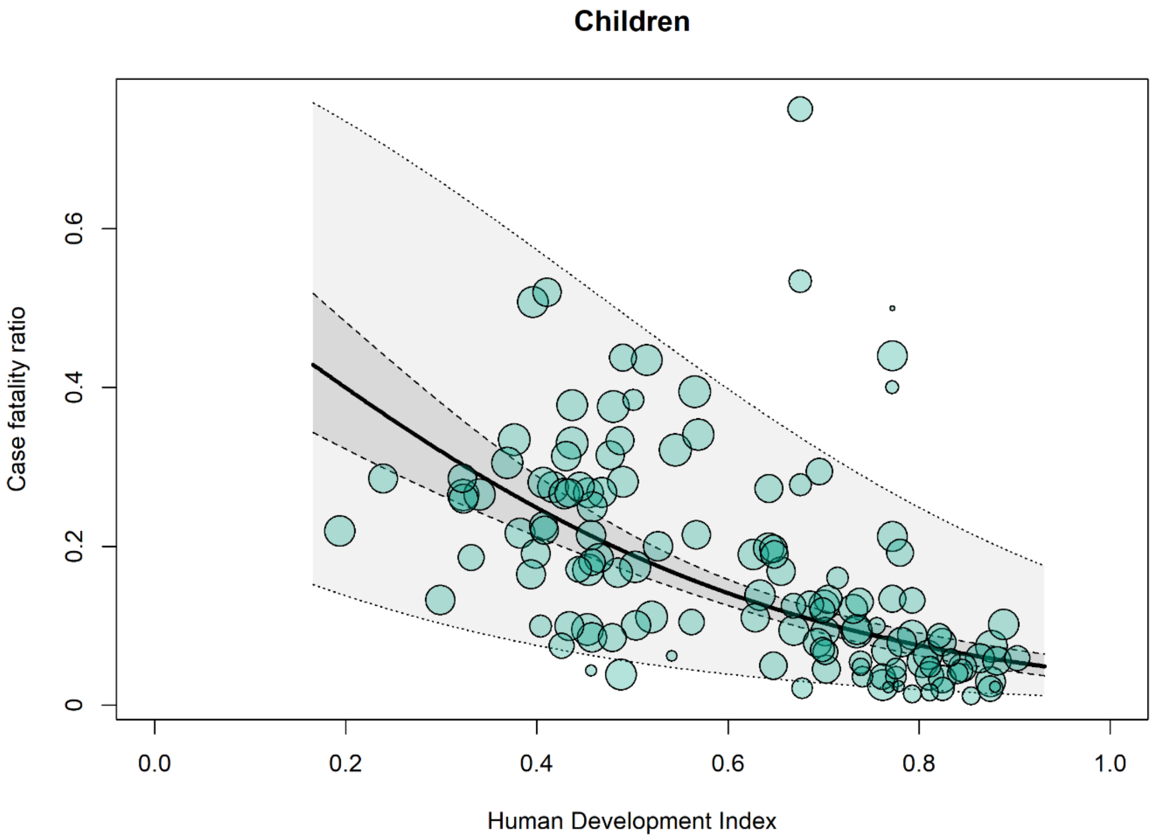
Children
Case fatality ratio
0
0.2
0.4
0.6
0.0
0.2
0.4
0.6
0.8
1.0
Human Development Index

C

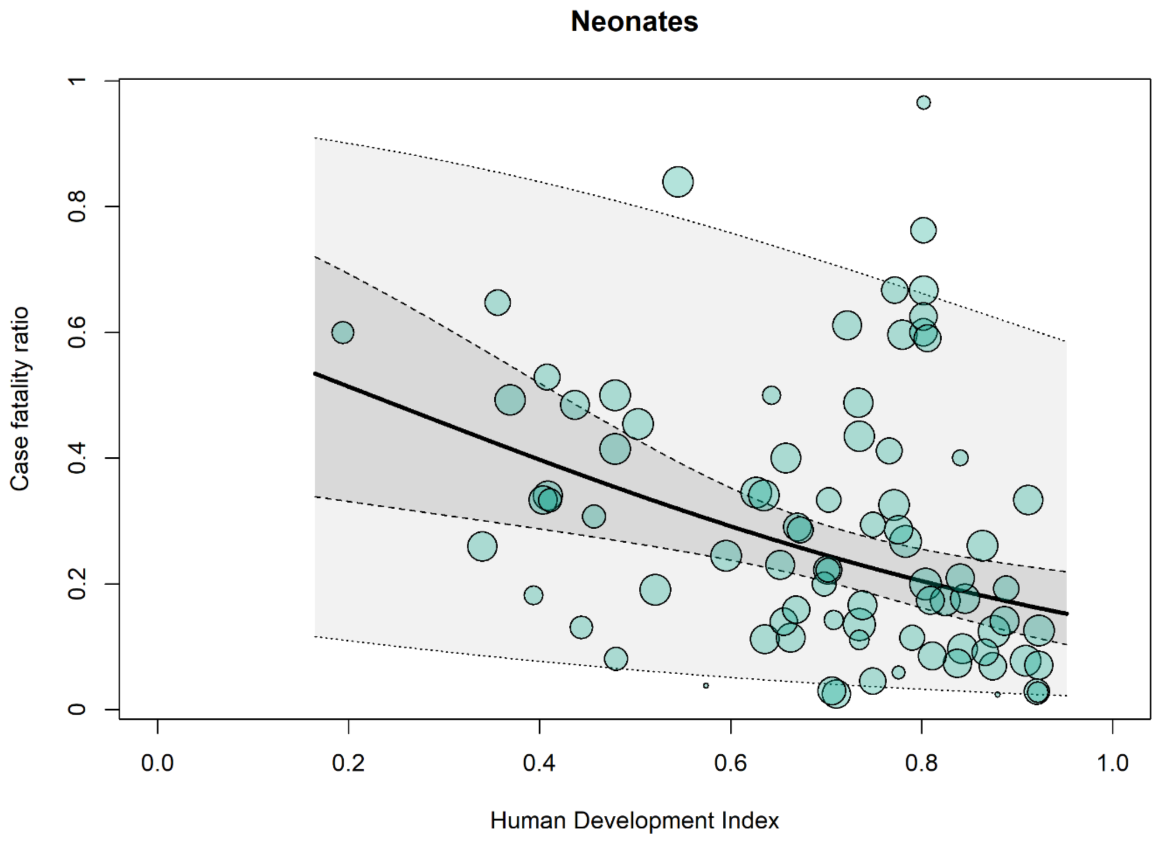
Neonates
Case fatality ratio
0
0.2
0.4
0.6
0.8
1
0.0
0.2
0.4
0.6
0.8
1.0
Human Development Index

Supplementary Figure 18. Case fatality ratio in bacterial meningitis with Gender Inequality Index (GII) as predictor. Studies with a mean observation period 1990 or later were included.

(dashed lines, 95% CI; dotted lines, prediction interval)

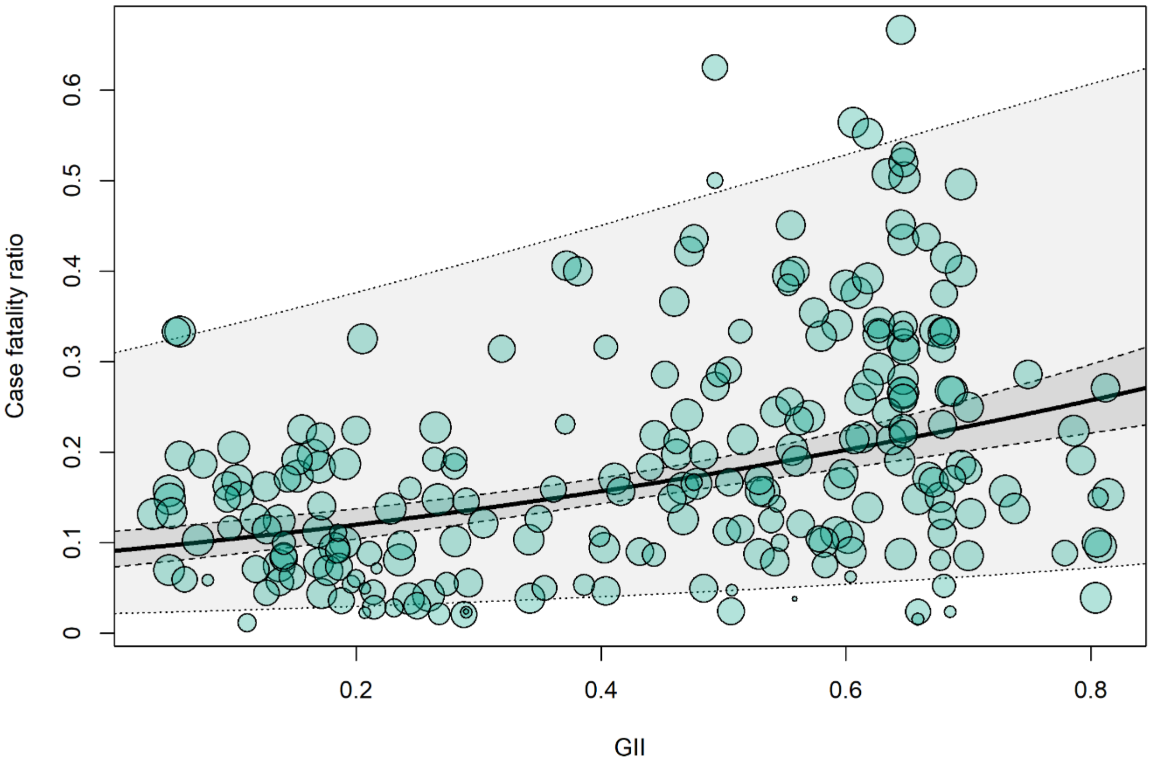

Supplementary Figure 19. Case fatality ratio in bacterial meningitis with Gender Inequality Index (GII) as predictor, stratified by the age group (**A**, adults, ≥ 16 years old; **B**, children 2 months to 16 years old; **C**, neonates, < 2 months old). Studies with a mean observation period 1990 or later were included.

(Dashed lines, 95% CI; dotted lines, prediction interval)

A

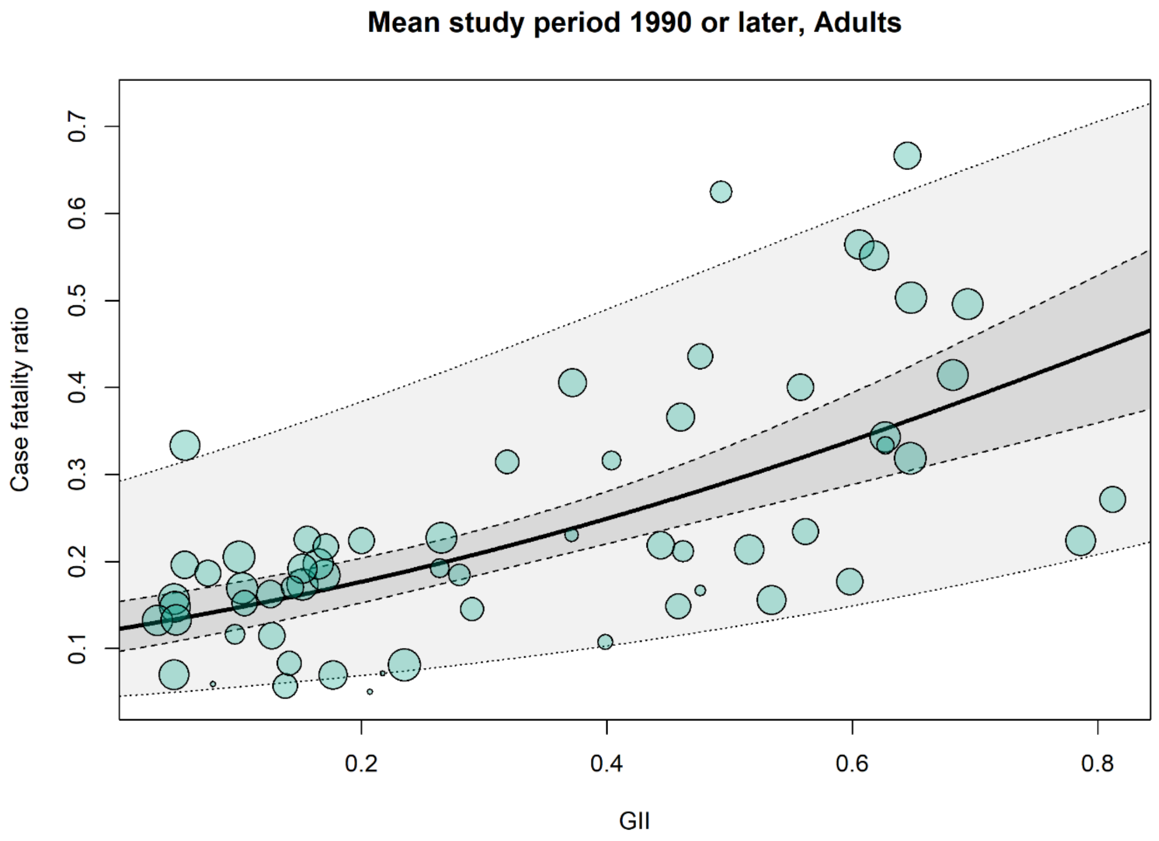

B

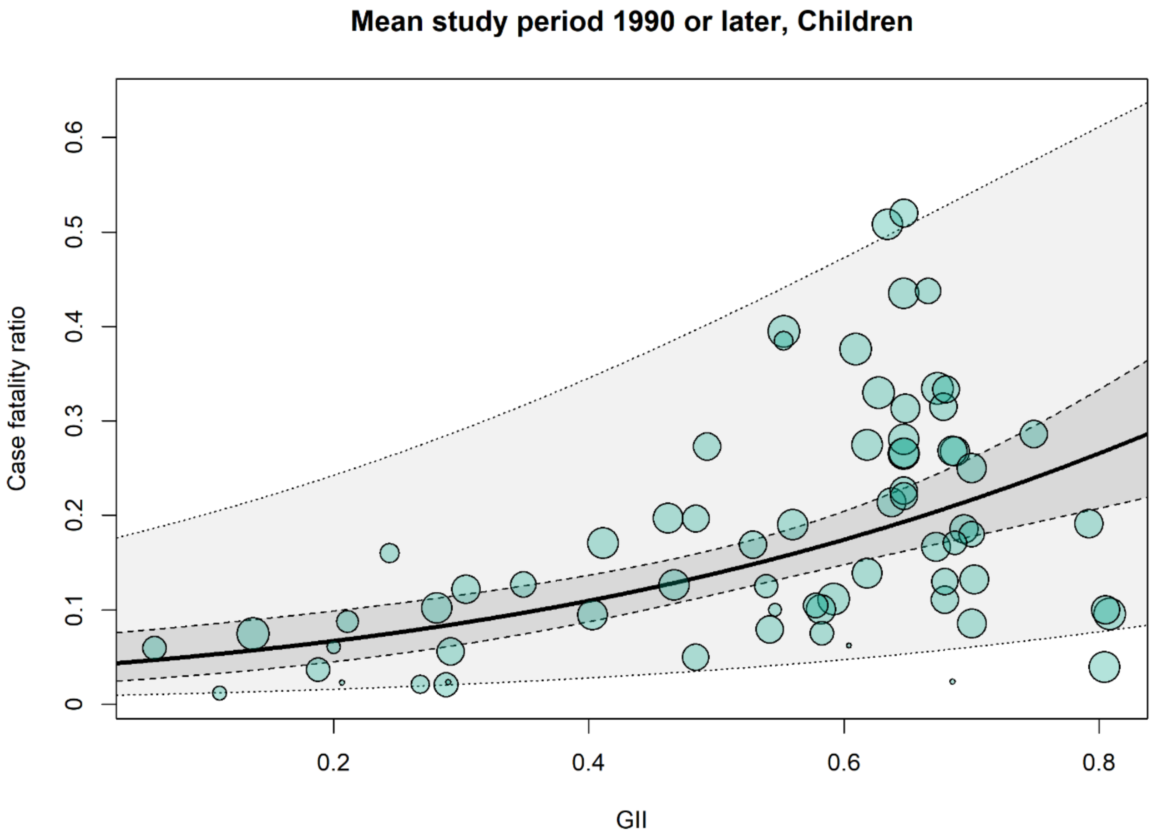
Mean study period 1990 or later, Children
Case fatality ratio
0
0.1
0.2
0.3
0.4
0.5
0.6
0.2
0.4
0.6
0.8
GII

C

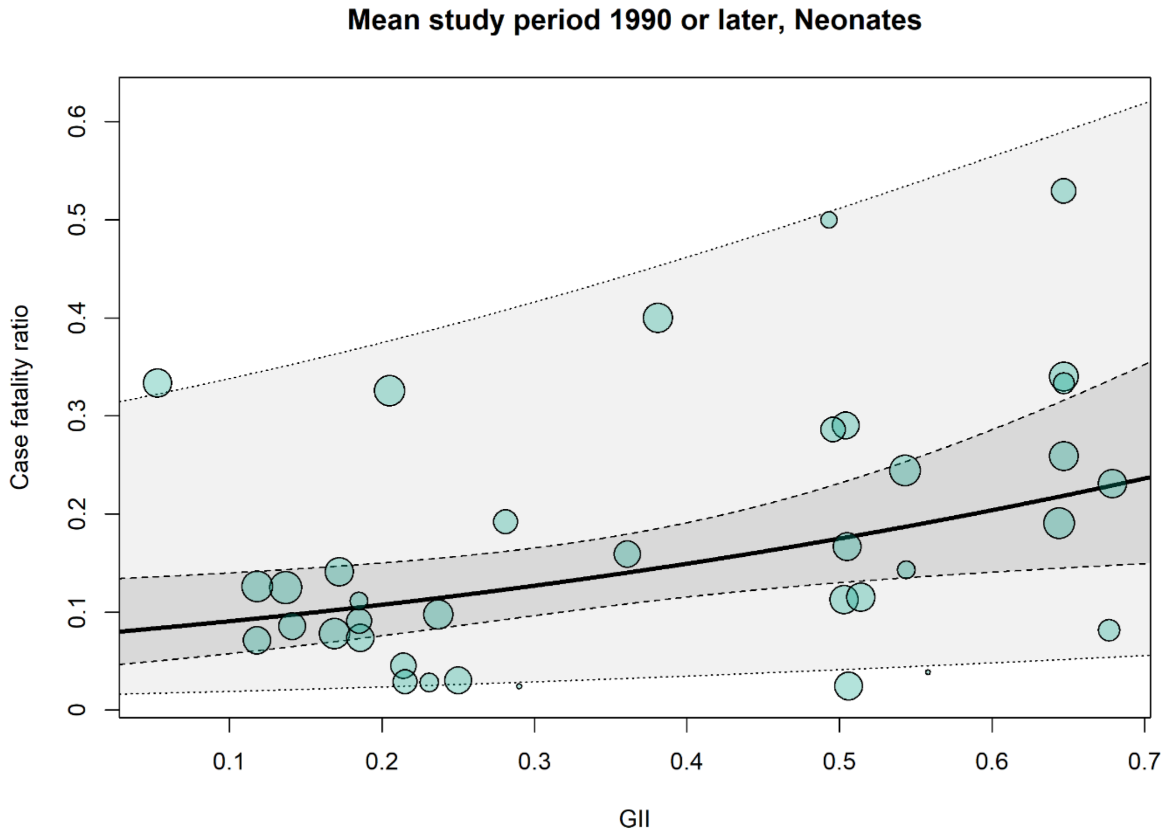
Mean study period 1990 or later, Neonates
Case fatality ratio
0
0.1
0.2
0.3
0.4
0.5
0.6
0.1
0.2
0.3
0.4
0.5
0.6
0.7
GII

Supplementary Figure 20. Case fatality ratio in bacterial meningitis with the Gini Index as predictor. Studies with a mean observation period 1960 or later were included.

(Dashed lines, 95% CI; dotted lines, prediction interval)

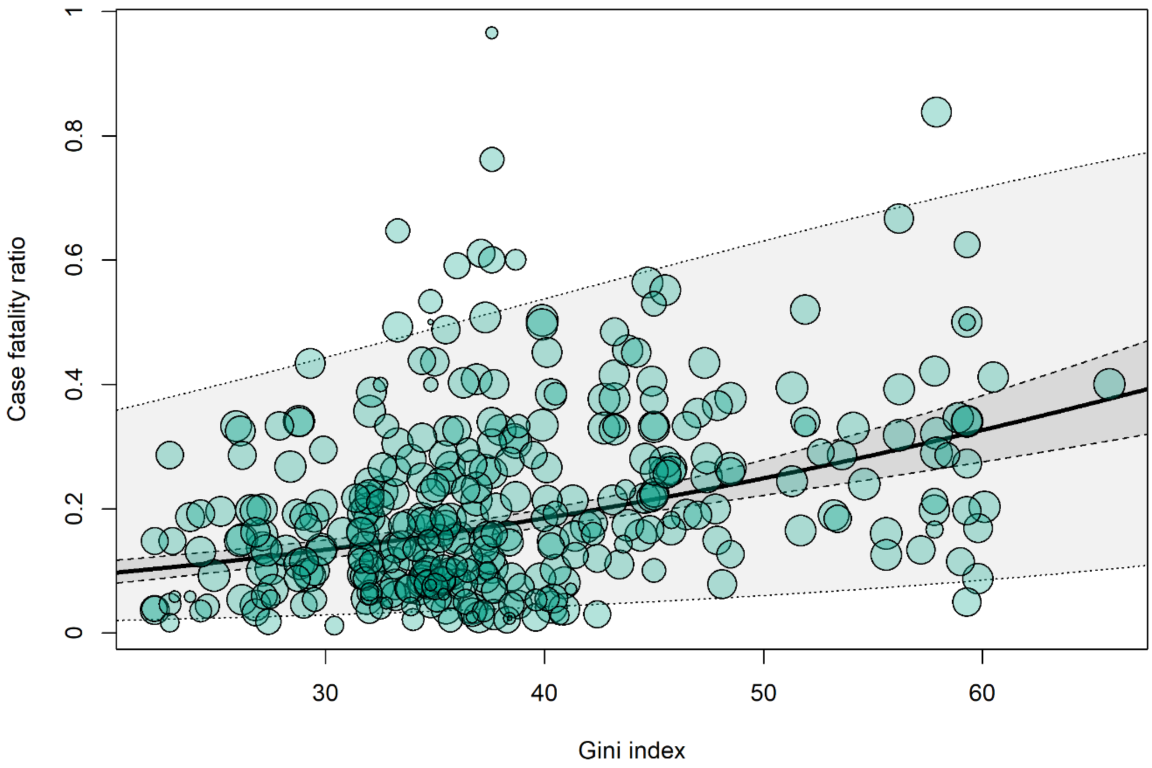

Supplementary Figure 21. Case fatality ratio in bacterial meningitis with the Gini Index as predictor, stratified by the age group (**A**, adults, ≥ 16 years old; **B**, children 2 months to 16 years old; **C**, neonates, < 2 months old).

(dashed lines, 95% CI; dotted lines, prediction interval)

A

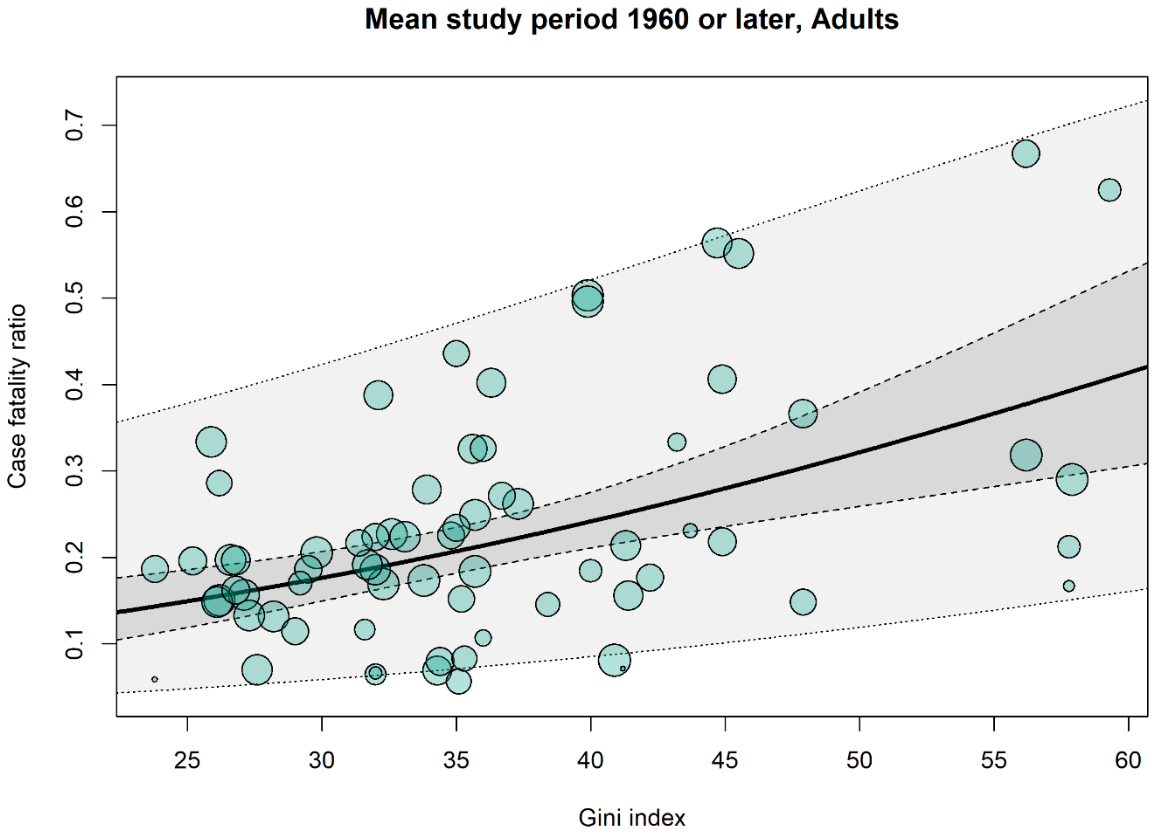

B

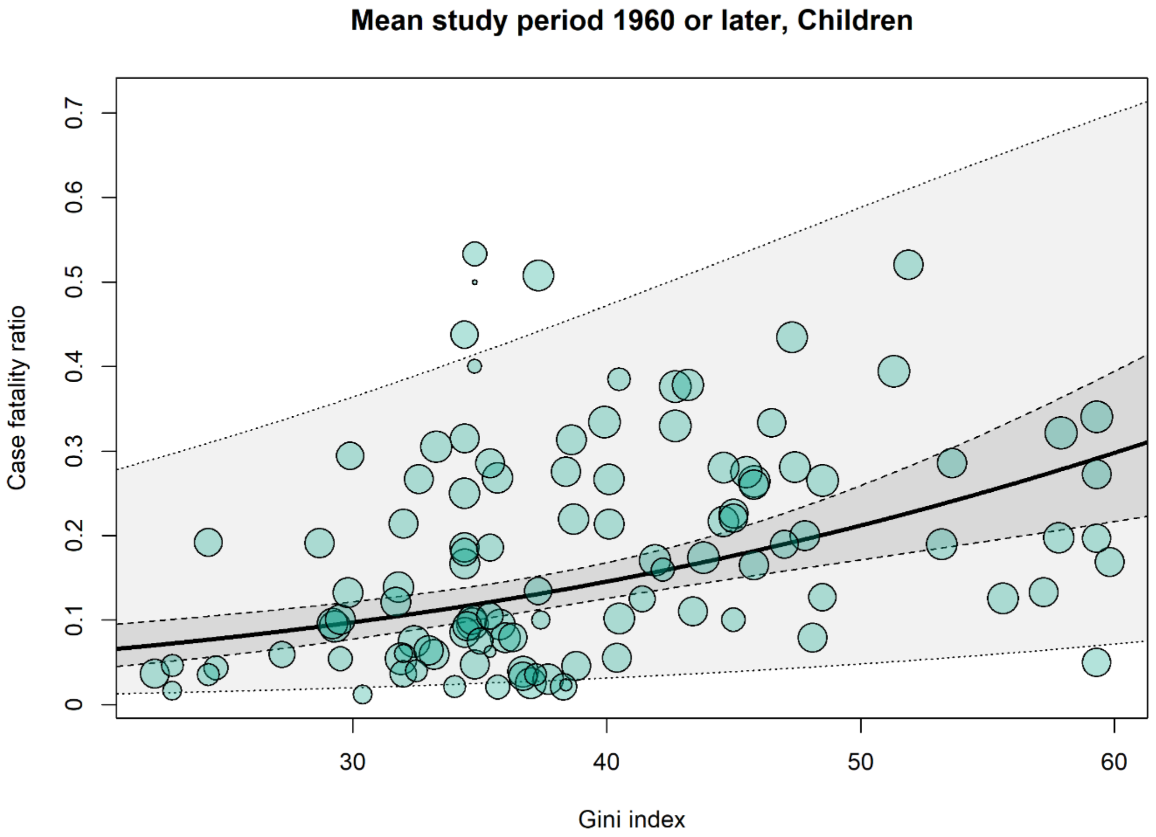
Mean study period 1960 or later, Children
Case fatality ratio
0
0.1
0.2
0.3
0.4
0.5
0.6
0.7
30
40
50
60
Gini index

C

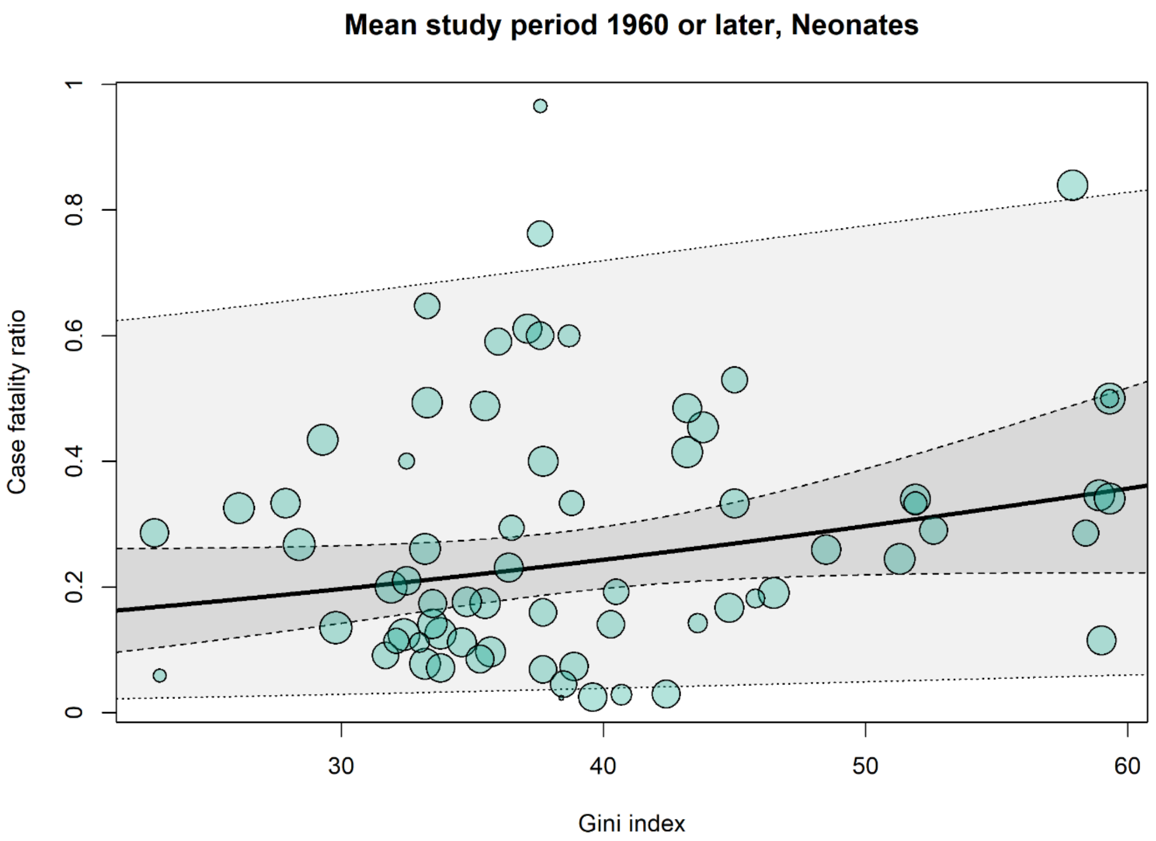
Mean study period 1960 or later, Neonates
Case fatality ratio
0
0.2
0.4
0.6
0.8
1
30
40
50
60
Gini index

Supplementary Figure 22. Case fatality ratio in bacterial meningitis with the Human Development Index as predictor (including mean study periods after 2000, sensitivity analysis).

(per HDI point, -2.0, 95% CI -2.8 to -1.3; R^2 21%; I^2 97%; k = 122; P < 0.001)

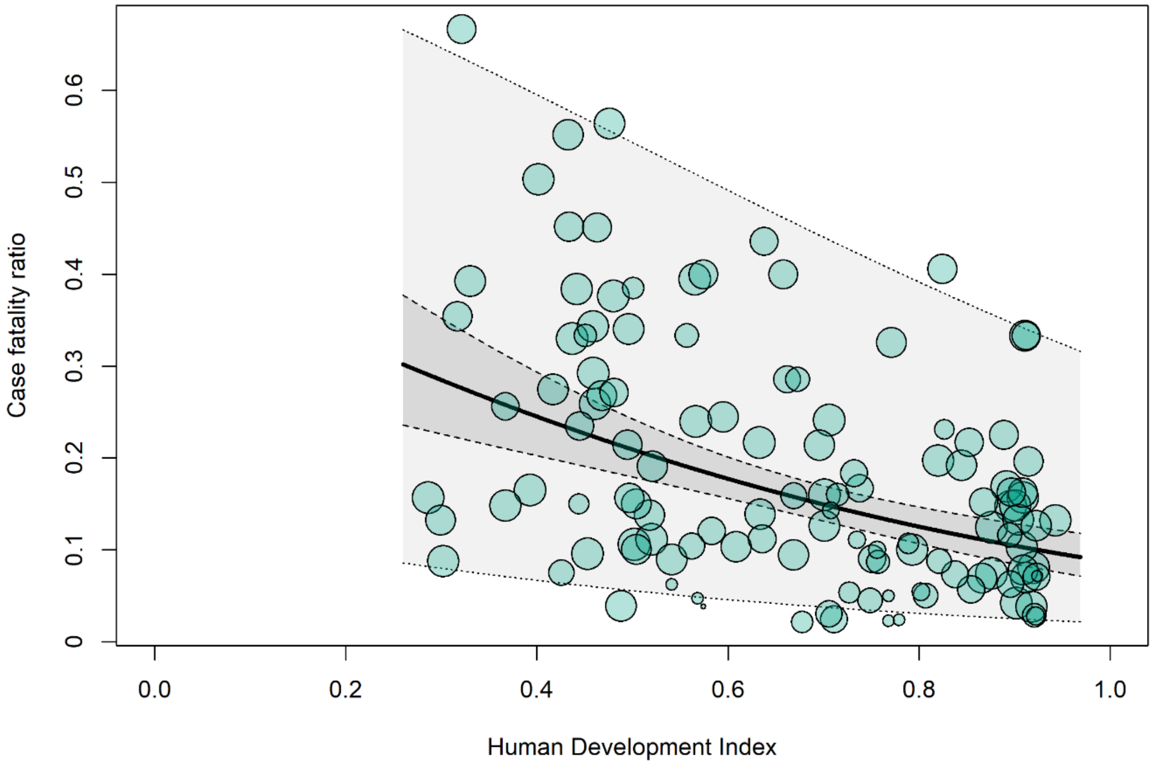

Supplementary Figure 23. Case fatality ratio in bacterial meningitis with the Gini Index as predictor (including mean study periods after 2000, sensitivity analysis).

(per Gini index point, 0.035, 95% CI 0.014 to 0.056; R^2 9%; I^2 96%; k = 109; P = 0.003)

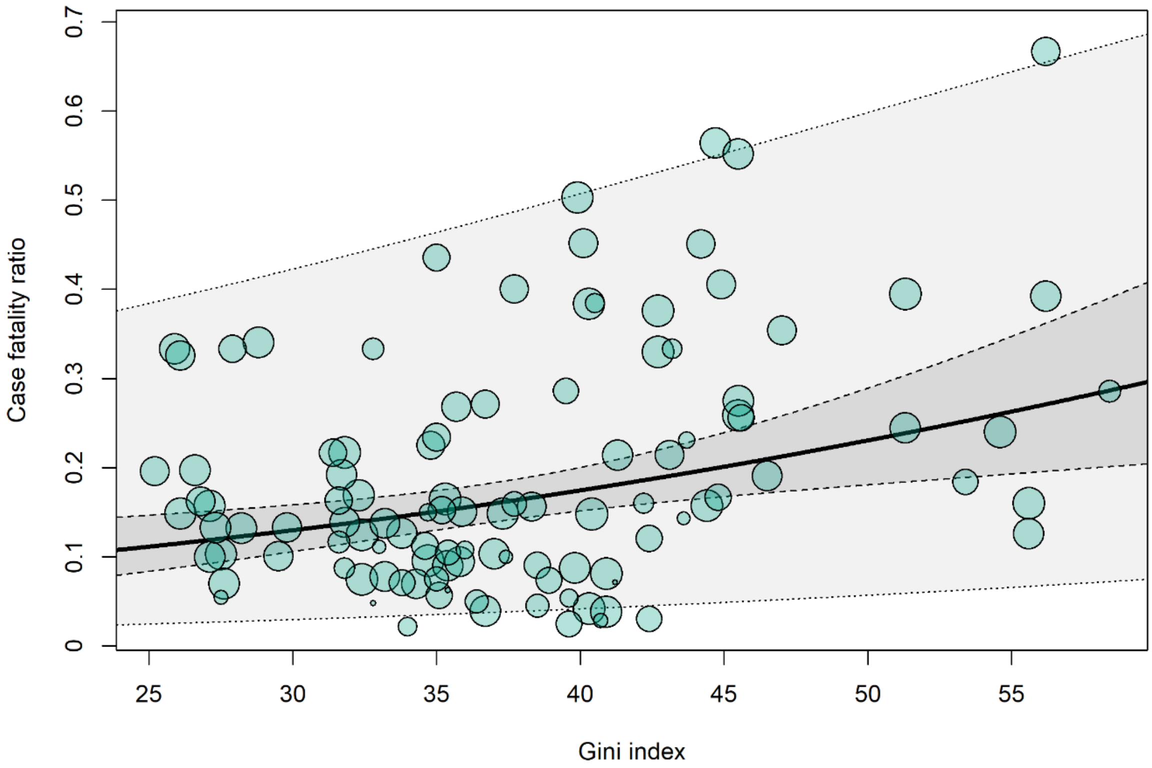

Supplementary Figure 24. Male-to-female case fatality ratio (CFR) ratio in bacterial meningitis with Gender Inequality Index as predictor (mean study period 1990 or later).

(Dashed lines, 95% CI; dotted lines, prediction interval)

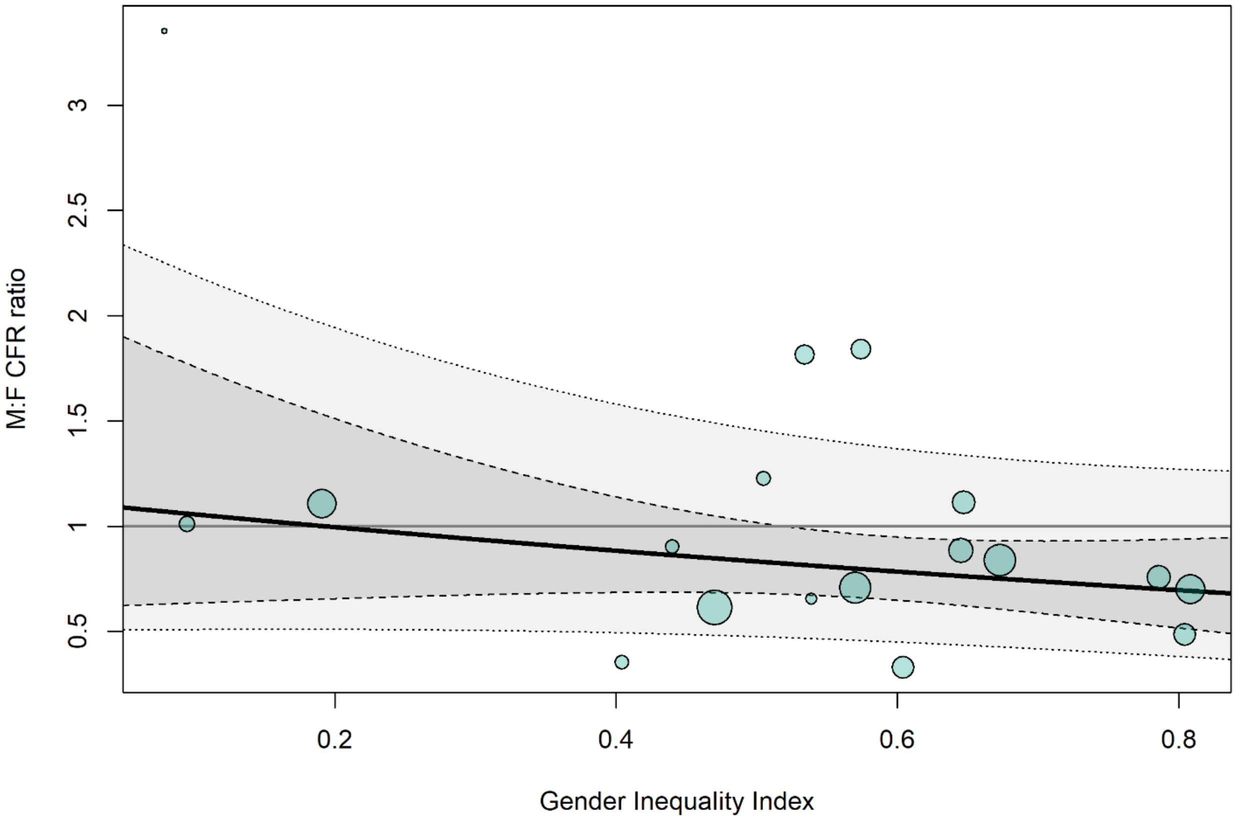

REFERENCES

1. Brainerd H, Bradley E. Treatment of Bacterial Meningitis with Penicillin, Sulfonamides, and Sera. *Calif Med* 1947; **66**(2): 57-62.
2. Smith ES. Purulent meningitis in infants and children; a review of 409 cases. *J Pediatr* 1954; **45**(4): 425-36.
3. Watson DG. Purulent neonatal meningitis; a study of forty-five cases. *J Pediatr* 1957; **50**(3): 352-60.
4. Shaper AG, Shaper L. Analysis of medical admissions to Mulago Hospital, 1957. *East Afr Med J* 1958; **35**(12): 647-78.
5. Eigler JO, Wellman WE, Rooke ED, Keith HM, Svien HJ. Bacterial meningits. I. General review (294 cases). *Proc Staff Meet Mayo Clin* 1961; **36**: 357-65.
6. Esrachowitz SR. Pyogenic meningitis--a study of 303 cases. *S Afr Med J* 1961; **35**: 101-4.
7. Groover RV, Sutherland JM, Landing BH. Purulent meningitis of newborn infants. Eleven-year experience in the antibiotic era. *N Engl J Med* 1961; **264**: 1115-21.
8. Carpenter RR, Petersdorf RG. The clinical spectrum of bacterial meningitis. *Am J Med* 1962; **33**: 262-75.
9. Quaade F, Kristensen KP. Purulent meningitis. A review of 658 cases. *Acta Med Scand* 1962; **171**: 543-50.
10. Yu JS, Grauaug A. Purulent Meningitis in the Neonatal Period. *Arch Dis Child* 1963; **38**(200): 391-6.
11. Gossage JD. Acute Purulent Meningitis in Children: Experience at the Hospital for Sick Children, Toronto. *Can Med Assoc J* 1964; **90**(10): 615-7.
12. Heycock JB, Noble TC. Pyogenic Meningitis in Infancy and Childhood. *Br Med J* 1964; **1**(5384): 658-62.
13. Wilson FM, Lerner AM. Etiology and Mortality of Purulent Meningitis at the Detroit Receiving Hospital. *N Engl J Med* 1964; **271**: 1235-8.
14. Schmuziger P, Wegmann T. [Purulent Meningitis--Therapy and Prognosis]. *Schweiz Med Wochenschr* 1965; **95**: 149-61.
15. Swartz MN, Dodge PR. Bacterial Meningitis--a Review of Selected Aspects. 1. General Clinical Features, Special Problems and Unusual Meningeal Reactions Mimicking Bacterial Meningitis. *N Engl J Med* 1965; **272**: 725-31 CONTD.
16. Berman PH, Banker BQ. Neonatal meningitis. A clinical and pathological study of 29 cases. *Pediatrics* 1966; **38**(1): 6-24.
17. Fortuine R. Acute purulent meningitis in Alaska natives: epidemiology, diagnosis and prognosis. *Can Med Assoc J* 1966; **94**(1): 19-22.
18. McNiel JR. Acute bacterial meningitis as seen in children of Eastern Saudi Arabia. *Clin Pediatr (Phila)* 1966; **5**(7): 437-8.
19. Donald G, McKendrick W. The treatment of pyogenic meningitis. *J Neurol Neurosurg Psychiatry* 1968; **31**(5): 528-31.
20. Fosson AR, Fine RN. Neonatal meningitis. Presentation and discussion of 21 cases. *Clin Pediatr (Phila)* 1968; **7**(7): 404-10.
21. Chevrie JJ, Aicardi J. Bacterial meningitis among newborn infants. *Clin Pediatr (Phila)* 1969; **8**(10): 562-3.
22. Justitz B. [Purulent meningitis in childhood with special reference to fatal clinical cases and defective healing since the introduction of antibiotic treatment]. *Arch Kinderheilkd* 1970; **181**(1): 40-62.
23. Overall JC, Jr. Neonatal bacterial meningitis. Analysis of predisposing factors and outcome compared with matched control subjects. *J Pediatr* 1970; **76**(4): 499-511.
24. Seriki O. Pyogenic meningitis in infancy and childhood. A survey of this disease based on observations of 156 African patients with a high mortality rate. *Clin Pediatr (Phila)* 1970; **9**(1): 17-21.
25. Jonsson M, Alvin A. A 12-year review of acute bacterial meningitis in Stockholm. *Scand J Infect Dis* 1971; **3**(2): 141-50.
26. Kendall AC. Acute bacterial meningitis in childhood. *Cent Afr J Med* 1971; **17**(5): 98-101.
27. McDonald R. Purulent meningitis in newborn babies: observations and comments based on a series of 82 patients. *Clin Pediatr (Phila)* 1972; **11**(8): 450-4.

28. Wiebe RA, Crast FW, Hall RA, Bass JW. Clinical factors relating to prognosis of bacterial meningitis. *South Med J* 1972; **65**(3): 257-64.

29. Fraser DW, Henke CE, Feldman RA. Changing patterns of bacterial meningitis in Olmsted County, Minnesota, 1935-1970. *J Infect Dis* 1973; **128**(3): 300-7.

30. Fraser DW, Darby CP, Koehler RE, Jacobs CF, Feldman RA. Risk factors in bacterial meningitis: Charleston County, South Carolina. *J Infect Dis* 1973; **127**(3): 271-7.

31. Floyd RF, Federspiel CF, Schaffner W. Bacterial meningitis in urban and rural Tennessee. *Am J Epidemiol* 1974; **99**(6): 395-407.

32. Fraser DW, Geil CC, Feldman RA. Bacterial meningitis in Bernalillo County, New Mexico: a comparison with three other American populations. *Am J Epidemiol* 1974; **100**(1): 29-34.

33. Santhanakrishnan BR, Baliga R, Raju VB. Purulent meningitis in the new born. *Indian J Pediatr* 1974; **41**(317): 218-23.

34. Chintu C, Bathirunathan N. Bacterial meningitis in infancy and childhood in Lusaka (One year prospective sturdy). *Med J Zambia* 1975; **9**(6): 150-7.

35. Fraser DW, Mitchell JE, Silverman LP, Feldman RA. Undiagnosed bacterial meningitis in Vermont children. *Am J Epidemiol* 1975; **102**(5): 394-9.

36. Hashemi C. Acute bacterial meningitis. Factors related to prognosis. *Indian J Pediatr* 1975; **42**(330): 209-14.

37. Hodges GR, Perkins RL. Acute bacterial meningitis: an analysis of factors influencing prognosis. *Am J Med Sci* 1975; **270**(3): 427-40.

38. Kaiser E, Fulop T, Szabo K. Purulent meningitis in infancy and childhood. *Acta Paediatr Acad Sci Hung* 1975; **16**(1): 13-22.

39. Dawson KP, Hammond N. Bacterial meningitis: a review of 53 patients. *N Z Med J* 1976; **84**(575): 351-3.

40. Goldacre MJ. Acute bacterial meningitis in childhood. Incidence and mortality in a defined population. *Lancet* 1976; **1**(7949): 28-31.

41. Yeung CY. Intrathecal antibiotic therapy for neonatal meningitis. *Arch Dis Child* 1976; **51**(9): 686-90.

42. Agranat O, Melmed S, Altmann G, Bank H. Bacterial and fungal meningitis in adults: a 22-year survey in a large community hospital in Israel. *Isr J Med Sci* 1977; **13**(12): 1151-62.

43. Finland M, Barnes MW. Acute bacterial meningitis at Boston City Hospital during 12 selected years, 1935-1972. *J Infect Dis* 1977; **136**(3): 400-15.

44. Gilsdorf JR. Bacterial meningitis in southwestern Alaska. *Am J Epidemiol* 1977; **106**(5): 388-91.

45. Lang SD. Bacterial meningitis in children. *N Z Med J* 1977; **86**(601): 511-4.

46. Moazami R, Raafat F, Aftandelians R, Arbabzadeh F, Erfani A. Acute bacterial meningitis in children (a retrospective survey of six and half years at a major pediatric center in Tehran, Iran). *Paediatr Indones* 1977; **17**(9-10): 281-8.

47. Hailemeskel H, Tafari N. Bacterial meningitis in childhood in an African city. Factors influencing aetiology and outcome. *Acta Paediatr Scand* 1978; **67**(6): 725-30.

48. Bieler-Niederer E. [Bacterial meningitis in newborn infants. A retrospective study from a pediatric clinic 1967-1978]. *Helv Paediatr Acta* 1979; **34**(6): 563-76.

49. Chattopadhyay B. Mortality from meningitis in a district general hospital--a review of 67 cases. *Public Health* 1980; **94**(2): 71-7.

50. Geiseler PJ, Nelson KE, Levin S, Reddi KT, Moses VK. Community-acquired purulent meningitis: a review of 1,316 cases during the antibiotic era, 1954-1976. *Rev Infect Dis* 1980; **2**(5): 725-45.

51. Horwitz SJ, Boxerbaum B, O'Bell J. Cerebral herniation in bacterial meningitis in childhood. *Ann Neurol* 1980; **7**(6): 524-8.

52. Perez-Yarza EG, Ruiz Benito C, Zudaire J, Solorzano C, Perez Trallero E. [Infections of the central nervous system. A review of 295 cases (author's transl)]. *An Esp Pediatr* 1980; **13**(5): 381-90.

53. Shann F, Germer S. Treatment of bacterial meningitis in children without intravenous fluids. *Med J Aust* 1981; **1**(11): 577-8.

54. Wotton KA, Stiver HG, Hildes JA. Meningitis in the central Arctic: a 4-year experience. *Can Med Assoc J* 1981; **124**(7): 887-90.

55. Davey PG, Cruikshank JK, McManus IC, Mahood B, Snow MH, Geddes AM. Bacterial meningitis--ten years experience. *J Hyg (Lond)* 1982; **88**(3): 383-401.
56. Guggenbichler JP. [Purulent meningitis in children. II. Treatment and prognosis]. *Padiatr Padol* 1982; **17**(1): 43-65.
57. Helwig H. [Right and wrong ways to treat meningitis (author's transl)]. *Monatsschr Kinderheilkd* 1982; **130**(5): 307-11.
58. Onile BA, Montefiore DG, Alausa OK, Ashiru JO. Bacterial meningitis: the first documentation of an epidemic in southern Nigeria. *Trans R Soc Trop Med Hyg* 1982; **76**(1): 41-4.
59. Bohr V, Hansen B, Jessen O, et al. Eight hundred and seventy-five cases of bacterial meningitis. Part I of a three-part series: clinical data, prognosis, and the role of specialised hospital departments. *J Infect* 1983; **7**(1): 21-30.
60. Guirguis N, Hafez K, El Kholy MA, Robbins JB, Gotschlich EC. Bacterial meningitis in Egypt: analysis of CSF isolates from hospital patients in Cairo, 1977-78. *Bull World Health Organ* 1983; **61**(3): 517-24.
61. Ispahani P. Bacterial meningitis in Nottingham. *J Hyg (Lond)* 1983; **91**(2): 189-201.
62. Gorse GJ, Thrupp LD, Nudleman KL, Wyle FA, Hawkins B, Cesario TC. Bacterial meningitis in the elderly. *Arch Intern Med* 1984; **144**(8): 1603-7.
63. McCracken GH, Jr. Management of bacterial meningitis in infants and children. Current status and future prospects. *Am J Med* 1984; **76**(5A): 215-23.
64. Mulder CJ, Zanen HC. A study of 280 cases of neonatal meningitis in The Netherlands. *J Infect* 1984; **9**(2): 177-84.
65. Mulla MI, Moosajee I, Rubidge CJ, Moosa A. Nutritional status of children with pyogenic meningitis. *J Trop Pediatr* 1984; **30**(6): 303-6.
66. Rodriguez WJ, Khan WN, Gold B, Feris J, Puig J, Sturla C. Ceftazidime in the treatment of meningitis in infants and children over one month of age. *Am J Med* 1985; **79**(2A): 52-5.
67. Schlech WF, 3rd, Ward JI, Band JD, Hightower A, Fraser DW, Broome CV. Bacterial meningitis in the United States, 1978 through 1981. The National Bacterial Meningitis Surveillance Study. *JAMA* 1985; **253**(12): 1749-54.
68. Shann F, Barker J, Poore P. Chloramphenicol alone versus chloramphenicol plus penicillin for bacterial meningitis in children. *Lancet* 1985; **2**(8457): 681-4.
69. Skoch MG, Walling AD. Meningitis: describing the community health problem. *American journal of public health* 1985; **75**(5): 550-2.
70. Valmari P. Primary diagnosis in a life-threatening childhood infection. A nationwide study on bacterial meningitis. *Ann Clin Res* 1985; **17**(6): 310-5.
71. Benderly A, Shehadeh N, Grief Z, Hayek T, Erde P, Etzioni A. Bacterial meningitis in infants two to six weeks old. *Helv Paediatr Acta* 1986; **41**(4): 311-5.
72. Jadavji T, Biggar WD, Gold R, Prober CG. Sequelae of acute bacterial meningitis in children treated for seven days. *Pediatrics* 1986; **78**(1): 21-5.
73. Rantakallio P, Leskinen M, von Wendt L. Incidence and prognosis of central nervous system infections in a birth cohort of 12,000 children. *Scand J Infect Dis* 1986; **18**(4): 287-94.
74. Yost GC, Kaplan AM, Bustamante R, Ellison C, Hargrave AF, Randall DL. Bacterial meningitis in Arizona American Indian children. *American journal of diseases of children (1960)* 1986; **140**(9): 943-6.
75. Bennhagen R, Svenningsen NW, Bekassy AN. Changing pattern of neonatal meningitis in Sweden. A comparative study 1976 vs. 1983. *Scand J Infect Dis* 1987; **19**(6): 587-93.
76. Girgis NI, Abu el Ella AH, Farid Z, Woody JN, Lissner C. Ceftriaxone compared with a combination of ampicillin and chloramphenicol in the treatment of bacterial meningitis in adults. *Drugs Exp Clin Res* 1987; **13**(8): 497-500.
77. Girgis NI, Abu el-Ella AH, Farid Z, Haberberger RL, Woody JN. Ceftriaxone alone compared to ampicillin and chloramphenicol in the treatment of bacterial meningitis. *Chemotherapy* 1988; **34 Suppl 1**: 16-20.
78. Kilpatrick ME, Mikhail IA, Girgis NI. Negative cultures of cerebrospinal fluid in partially treated bacterial meningitis. *Trop Geogr Med* 1987; **39**(4): 345-9.
79. Mir F, Aman S, Raza Khan S. Neonatal sepsis: a review with a study of 50 cases. *J Trop Pediatr* 1987; **33**(3): 131-5.

80. Salwen KM, Vikerfors T, Olcen P. Increased incidence of childhood bacterial meningitis. A 25-year study in a defined population in Sweden. *Scand J Infect Dis* 1987; **19**(1): 1-11.

81. Zimmerli W, Egli TF, Ritz R. [Prognostic factors in bacterial meningitis in adults. Retrospective analysis of 46 patients]. *Schweiz Med Wochenschr* 1987; **117**(23): 861-7.

82. Dawson KP, Abbott GD, Mogridge N. Bacterial meningitis in childhood: a 13 year review. *N Z Med J* 1988; **101**(857): 758-60.

83. Bell AH, Brown D, Halliday HL, McClure G, McReid M. Meningitis in the newborn--a 14 year review. *Arch Dis Child* 1989; **64**(6): 873-4.

84. Bhat BV, Verma IC, Puri RK, Srinivasan S, Nalini P. A profile of pyogenic meningitis in children. *J Indian Med Assoc* 1991; **89**(8): 224-7.

85. Cisse MF, Sow HD, Ouangre AR, Gaye A, Sow AI, Samb A, Fall M. [Bacterial meningitis in a pediatric hospital in a tropical zone]. *Med Trop (Mars)* 1989; **49**(3): 265-9.

86. Lim KW, Cheng HK. Bacterial meningitis--a four year survey in a paediatrics unit. *Ann Acad Med Singap* 1989; **18**(6): 649-54.

87. Martinez-Martin P, Saenz Lope E, Estevez Guerra E, Rapun Pac JL. [Infectious meningitis in the adult: 3-year clinical experience]. *Neurologia* 1989; **4**(3): 82-7.

88. Rosenthal J, Golan A, Dagan R. Bacterial meningitis with initial normal cerebrospinal fluid findings. *Isr J Med Sci* 1989; **25**(4): 186-8.

89. Sakakihara Y, Kamoshita S. Changing spectrum of pediatric neurologic disorders during 18 selected years, 1900-1980, at the Hospital of University of Tokyo. *Brain Dev* 1989; **11**(4): 251-6.

90. Shaltout AA, Auger LT, Awadallah NB, Hijazi Z, Johny M, Hajj KE, Kandil H. Morbidity and mortality of bacterial meningitis in Arab children. *J Trop Med Hyg* 1989; **92**(6): 402-6.

91. Bryan JP, de Silva HR, Tavares A, Rocha H, Scheld WM. Etiology and mortality of bacterial meningitis in northeastern Brazil. *Rev Infect Dis* 1990; **12**(1): 128-35.

92. Carter PE, Barclay SM, Galloway WH, Cole GF. Changes in bacterial meningitis. *Arch Dis Child* 1990; **65**(5): 495-8.

93. Choo KE, Ariffin WA, Ahmad T, Lim WL, Gururaj AK. Pyogenic meningitis in hospitalized children in Kelantan, Malaysia. *Ann Trop Paediatr* 1990; **10**(1): 89-98.

94. de Bary JB, Soro B, Seynaeve V, Schuermann L, Rey JL. [Purulent meningitis at a semi-rural hospital in the forest area of the Ivory Coast]. *Bull Soc Pathol Exot* 1990; **83**(4): 460-7.

95. Pomeroy SL, Holmes SJ, Dodge PR, Feigin RD. Seizures and other neurologic sequelae of bacterial meningitis in children. *N Engl J Med* 1990; **323**(24): 1651-7.

96. Salih MA. Childhood acute bacterial meningitis in the Sudan: an epidemiological, clinical and laboratory study. *Scand J Infect Dis Suppl* 1990; **66**: 1-103.

97. Wenger JD, Hightower AW, Facklam RR, Gaventa S, Broome CV. Bacterial meningitis in the United States, 1986: report of a multistate surveillance study. The Bacterial Meningitis Study Group. *J Infect Dis* 1990; **162**(6): 1316-23.

98. Zaki M, Daoud AS, al Saleh Q, Abd al Rasool MM. Bacterial meningitis in the newborn: a Kuwaiti experience. *J Trop Pediatr* 1990; **36**(2): 63-5.

99. Zaki M, Daoud AS, ElSaleh Q, West PW. Childhood bacterial meningitis in Kuwait. *J Trop Med Hyg* 1990; **93**(1): 7-11.

100. de Louvois J, Blackbourn J, Hurley R, Harvey D. Infantile meningitis in England and Wales: a two year study. *Arch Dis Child* 1991; **66**(5): 603-7.

101. Dufour JF, Waldvogel F. [Meningitis in adults in Geneva. Review of 257 cases]. *Schweiz Med Wochenschr Suppl* 1991; **35**: 1-37.

102. Hanna JN, Wild BE. Bacterial meningitis in children under five years of age in Western Australia. *Med J Aust* 1991; **155**(3): 160-4.

103. Kabra SK, Kumar P, Verma IC, et al. Bacterial meningitis in India: an IJP survey. *Indian J Pediatr* 1991; **58**(4): 505-11.

104. Minutillo C, Pemberton PJ, Cole M. Neonatal meningitis. *J Paediatr Child Health* 1991; **27**(3): 191-2.

105. Nathoo KJ, Pazvakavamba I, Chidede OS, Chirisa C. Neonatal meningitis in Harare, Zimbabwe: a 2-year review. *Ann Trop Paediatr* 1991; **11**(1): 11-5.
106. Olanrewaju DM, Olusanya O, Laditan AA. Acute bacterial meningitis in children. *West Afr J Med* 1991; **10**(1): 405-11.
107. Pecoul B, Varaine F, Keita M, et al. Long-acting chloramphenicol versus intravenous ampicillin for treatment of bacterial meningitis. *Lancet* 1991; **338**(8771): 862-6.
108. al-Jurayyan NA, al Mazyad AS, al-Nasser MN, al-Eissa YA, Abo-Bakr AM, Boohene AG, Familusi JB. Childhood bacterial meningitis in Al-Baha province, Saudi Arabia. *J Trop Med Hyg* 1992; **95**(3): 180-5.
109. Craig JC, Abbott GD, Mogridge NB. Ceftriaxone for paediatric bacterial meningitis: a report of 62 children and a review of the literature. *N Z Med J* 1992; **105**(945): 441-4.
110. Francis BM, Gilbert GL. Survey of neonatal meningitis in Australia: 1987-1989. *Med J Aust* 1992; **156**(4): 240-3.
111. Franco SM, Cornelius VE, Andrews BF. Long-term outcome of neonatal meningitis. *American journal of diseases of children (1960)* 1992; **146**(5): 567-71.
112. Mackie EJ, Shears P, Frimpong E, Mustafa-Kutana SN. A study of bacterial meningitis in Kumasi, Ghana. *Ann Trop Paediatr* 1992; **12**(2): 143-8.
113. Pallangyo K, Hakanson A, Lema L, et al. High HIV seroprevalence and increased HIV-associated mortality among hospitalized patients with deep bacterial infections in Dar es Salaam, Tanzania. *AIDS* 1992; **6**(9): 971-6.
114. Rasmussen HH, Sorensen HT, Moller-Petersen J, Mortensen FV, Nielsen B. Bacterial meningitis in elderly patients: clinical picture and course. *Age Ageing* 1992; **21**(3): 216-20.
115. Rothrock SG, Green SM, Wren J, Letai D, Daniel-Underwood L, Pillar E. Pediatric bacterial meningitis: is prior antibiotic therapy associated with an altered clinical presentation? *Ann Emerg Med* 1992; **21**(2): 146-52.
116. Shattuck KE, Chonmaitree T. The changing spectrum of neonatal meningitis over a fifteen-year period. *Clin Pediatr (Phila)* 1992; **31**(3): 130-6.
117. Srair HA, Aman H, al-Madan M, al-Khater M. Bacterial meningitis in Saudi children. *Indian J Pediatr* 1992; **59**(6): 719-21.
118. Tefuarani N, Vince JD. Purulent meningitis in children: outcome using a standard management regimen with chloramphenicol. *Ann Trop Paediatr* 1992; **12**(4): 375-83.
119. Thomas DG. Outcome of paediatric bacterial meningitis 1979-1989. *Med J Aust* 1992; **157**(8): 519-20.
120. Airede AI. Neonatal bacterial meningitis in the middle belt of Nigeria. *Dev Med Child Neurol* 1993; **35**(5): 424-30.
121. Ballantyne ES, Chaseling R, Miller JD. When should patients with bacterial meningitis be referred to a neurosurgical unit? *Scott Med J* 1993; **38**(3): 77-9.
122. Boehme C, Soto L, Rodriguez G, Serra J, Illesca V, Reydet P. [Three years of acute bacterial meningitis in the pediatric service at the Temuco Regional Hospital]. *Rev Med Chil* 1993; **121**(6): 633-8.
123. Brivet FG, Guibert M, Dormont J. Acute bacterial meningitis in adults. *N Engl J Med* 1993; **328**(23): 1712-3.
124. Durand ML, Calderwood SB, Weber DJ, Miller SI, Southwick FS, Caviness VS, Swartz MN. Acute bacterial meningitis in adults. A review of 493 episodes. *The New England journal of medicine* 1993; **328**(1): 21-8.
125. Fortnum HM, Davis AC. Epidemiology of bacterial meningitis. *Arch Dis Child* 1993; **68**(6): 763-7.
126. Liu CC, Chen JS, Lin CH, Chen YJ, Huang CC. Bacterial meningitis in infants and children in southern Taiwan: emphasis on Haemophilus influenzae type B infection. *J Formos Med Assoc* 1993; **92**(10): 884-8.
127. Pfister HW, Feiden W, Einhaupl KM. Spectrum of complications during bacterial meningitis in adults. Results of a prospective clinical study. *Arch Neurol* 1993; **50**(6): 575-81.
128. Ara JR, Cia P, Arribas JL, Aguirre JM, de Juan F, Marco Tello A. [Clinico-epidemiologic study of bacterial meningitis in Aragon]. *Med Clin (Barc)* 1994; **103**(16): 611-4.
129. Carroll KJ, Carroll C. A prospective investigation of the long-term auditory-neurological sequelae associated with bacterial meningitis: a study from Vanuatu. *J Trop Med Hyg* 1994; **97**(3): 145-50.
130. Chotpitayasunondh T. Bacterial meningitis in children: etiology and clinical features, an 11-year review of 618 cases. *Southeast Asian J Trop Med Public Health* 1994; **25**(1): 107-15.

131. Commey JO, Rodrigues OP, Akita FA, Newman M. Bacterial meningitis in children in southern Ghana. *East Afr Med J* 1994; **71**(2): 113-7.
132. Dagan R, Isaachson M, Lang R, Karpuch J, Block C, Amir J. Epidemiology of pediatric meningitis caused by Haemophilus influenzae type b, Streptococcus pneumoniae, and Neisseria meningitidis in Israel: a 3-year nationwide prospective study. Israeli Pediatric Bacteremia and Meningitis Group. *J Infect Dis* 1994; **169**(4): 912-6.
133. Ford H, Wright J. Bacterial meningitis in Swaziland: an 18 month prospective study of its impact. *J Epidemiol Community Health* 1994; **48**(3): 276-80.
134. Kallio MJ, Kilpi T, Anttila M, Peltola H. The effect of a recent previous visit to a physician on outcome after childhood bacterial meningitis. *JAMA* 1994; **272**(10): 787-91.
135. Moreno MT, Vargas S, Poveda R, Saez-Llorens X. Neonatal sepsis and meningitis in a developing Latin American country. *Pediatr Infect Dis J* 1994; **13**(6): 516-20.
136. Ozumba UC. Changing pattern of acute bacterial meningitis in Enugu, Nigeria. *East Afr Med J* 1994; **71**(5): 300-3.
137. Synnott MB, Morse DL, Hall SM. Neonatal meningitis in England and Wales: a review of routine national data. *Arch Dis Child Fetal Neonatal Ed* 1994; **71**(2): F75-80.
138. Ali Z. Neonatal meningitis: a 3-year retrospective study at the Mount Hope Women's Hospital, Trinidad, West Indies. *J Trop Pediatr* 1995; **41**(2): 109-11.
139. Almirante B, Cortes E, Pigrau C, Gasser I, del Valle O, Campos L, Pahissa A. [Treatment and outcome of pneumococcal meningitis in adults. Study of a recent series of 70 episodes]. *Med Clin (Barc)* 1995; **105**(18): 681-6.
140. Ciana G, Parmar N, Antonio C, Pivetta S, Tamburlini G, Cuttini M. Effectiveness of adjunctive treatment with steroids in reducing short-term mortality in a high-risk population of children with bacterial meningitis. *J Trop Pediatr* 1995; **41**(3): 164-8.
141. Daoud AS, al-Sheyyab M, Batchoun RG, Rawashdeh MO, Nussair MM, Pugh RN. Bacterial meningitis: still a cause of high mortality and severe neurological morbidity in childhood. *J Trop Pediatr* 1995; **41**(5): 308-10.
142. Gedlu E, Rahlenbeck SI. Pyogenic meningitis in children in north-western Ethiopia. *Ann Trop Paediatr* 1995; **15**(3): 243-7.
143. Kaaresen PI, Flaegstad T. Prognostic factors in childhood bacterial meningitis. *Acta Paediatr* 1995; **84**(8): 873-8.
144. Kilpi T, Peltola H, Jauhiainen T, Kallio MJ. Oral glycerol and intravenous dexamethasone in preventing neurologic and audiologic sequelae of childhood bacterial meningitis. The Finnish Study Group. *Pediatr Infect Dis J* 1995; **14**(4): 270-8.
145. Lecour H, Miranda AM, Nogueira JA, Abreu C. Update on the use of cefotaxime for pediatric meningitis in Portugal. *Diagn Microbiol Infect Dis* 1995; **22**(1-2): 125-7.
146. Lutsar I, Siirde T, Soopold T. Long term follow-up of Estonian children after bacterial meningitis. *Pediatr Infect Dis J* 1995; **14**(7): 624-5.
147. Patwari AK, Singh BS, Manorama DE. Inappropriate secretion of antidiuretic hormone in acute bacterial meningitis. *Ann Trop Paediatr* 1995; **15**(2): 179-83.
148. Salaun-Saraux P, Saraux A, Lepage P, et al. [Septic meningitis in children in Rwanda from 1983 to 1990. Retrospective study at the Kigali Hospital Center]. *Med Trop (Mars)* 1995; **55**(1): 41-5.
149. Singhi SC, Singhi PD, Srinivas B, Narakesri HP, Ganguli NK, Sialy R, Walia BN. Fluid restriction does not improve the outcome of acute meningitis. *Pediatr Infect Dis J* 1995; **14**(6): 495-503.
150. Ahmed AA, Salih MA, Ahmed HS. Post-endemic acute bacterial meningitis in Sudanese children. *East Afr Med J* 1996; **73**(8): 527-32.
151. Berg S, Trollfors B, Claesson BA, et al. Incidence and prognosis of meningitis due to Haemophilus influenzae, Streptococcus pneumoniae and Neisseria meningitidis in Sweden. *Scand J Infect Dis* 1996; **28**(3): 247-52.
152. Bergemann A, Karstaedt AS. The spectrum of meningitis in a population with high prevalence of HIV disease. *QJM* 1996; **89**(7): 499-504.
153. Gomes I, Melo A, Lucena R, et al. Prognosis of bacterial meningitis in children. *Arq Neuropsiquiatr* 1996; **54**(3): 407-11.

154. Ichiyama T, Hayashi T, Furukawa S. Cerebrospinal fluid concentrations of soluble tumor necrosis factor receptor in bacterial and aseptic meningitis. *Neurology* 1996; **46**(3): 837-8.
155. Ishikawa T, Asano Y, Morishima T, Nagashima M, Sobue G, Watanabe K, Yamaguchi H. Epidemiology of bacterial meningitis in children: Aichi Prefecture, Japan, 1984-1993. *Pediatr Neurol* 1996; **14**(3): 244-50.
156. Laguna del Estal P, Salgado Marques R, Calabrese Sanchez S, Murillas Angoitti J, Martin Alvarez E, Moya Mir MS. [Acute bacterial meningitis in adults: a clinical and developmental analysis of 100 cases]. *An Med Interna* 1996; **13**(11): 520-6.
157. Macaluso A, Pivetta S, Maggi RS, Tamburlini G, Cattaneo A. Dexamethasone adjunctive therapy for bacterial meningitis in children: a retrospective study in Brazil. *Ann Trop Paediatr* 1996; **16**(3): 193-8.
158. Qazi SA, Khan MA, Mughal N, et al. Dexamethasone and bacterial meningitis in Pakistan. *Arch Dis Child* 1996; **75**(6): 482-8.
159. Awasthi S, Moin S, Iyer SM, Rehman H. Modified Glasgow Coma Scale to predict mortality in children with acute infections of the central nervous system. *Natl Med J India* 1997; **10**(5): 214-6.
160. Grobler AC, Hay IT. Bacterial meningitis in children at Kalafong Hospital, 1990-1995. *S Afr Med J* 1997; **87**(8 Suppl): 1052-4.
161. Hussey G, Schaaf H, Hanslo D, et al. Epidemiology of post-neonatal bacterial meningitis in Cape Town children. *S Afr Med J* 1997; **87**(1): 51-6.
162. Imuekehme S, Obi J, Alakija W. Cerebro-spinal lactate status in childhood pyogenic meningitis in Nigeria. *J Trop Pediatr* 1997; **43**(6): 361-3.
163. Sigurdardottir B, Bjornsson OM, Jonsdottir KE, Erlendsdottir H, Gudmundsson S. Acute bacterial meningitis in adults. A 20-year overview. *Arch Intern Med* 1997; **157**(4): 425-30.
164. Sung RY, Senok AC, Ho A, Oppenheimer SJ, Davies DP. Meningitis in Hong Kong children, with special reference to the infrequency of haemophilus and meningococcal infection. *J Paediatr Child Health* 1997; **33**(4): 296-9.
165. Chang YC, Huang CC, Wang ST, Liu CC, Tsai JJ. Risk factors analysis for early fatality in children with acute bacterial meningitis. *Pediatr Neurol* 1998; **18**(3): 213-7.
166. Fernandez-Jaen A, Borque Andres C, del Castillo Martin F, Pena Garcia P, Vidal Lopez ML. [Bacterial meningitis in pediatrics. Study of 166 cases]. *An Esp Pediatr* 1998; **48**(5): 495-8.
167. Gutierrez A, Ramos MA, Sanz JC, Bernal A, Agirrezabal J, Casado Y, Martinez M. [Bacterial meningitis in emergency medicine. Factors associated with delay of antimicrobial therapy]. *Enferm Infecc Microbiol Clin* 1998; **16**(7): 302-6.
168. Honnas A, Petersen LT. Bacterial meningitis in a rural Kenyan hospital. *East Afr Med J* 1998; **75**(7): 396-401.
169. Hussain IH, Sofiah A, Ong LC, Choo KE, Musa MN, Teh KH, Ng HP. Haemophilus influenzae meningitis in Malaysia. *Pediatr Infect Dis J* 1998; **17**(9 Suppl): S189-90.
170. Imananagha KK, Peters EJ, Philip-Ephraim EE, Ekott JU, Imananagha LN, Ekure EN, Esin RA. Acute bacterial meningitis in a developing country: diagnosis related mortality among paediatric patients. *Cent Afr J Med* 1998; **44**(1): 11-5.
171. Kim KH, Sohn YM, Kang JH, et al. The causative organisms of bacterial meningitis in Korean children, 1986-1995. *J Korean Med Sci* 1998; **13**(1): 60-4.
172. Molyneux E, Walsh A, Phiri A, Molyneux M. Acute bacterial meningitis in children admitted to the Queen Elizabeth Central Hospital, Blantyre, Malawi in 1996-97. *Trop Med Int Health* 1998; **3**(8): 610-8.
173. Muller M, Merkelbach S, Hermes M, Konig J, Schimrigk K. Relationship between short-term outcome and occurrence of cerebral artery stenosis in survivors of bacterial meningitis. *J Neurol* 1998; **245**(2): 87-92.
174. Rios-Reategui E, Ruiz-Gonzalez L, Murguia-de-Sierra T. [Neonatal bacterial meningitis in a tertiary treatment center]. *Rev Invest Clin* 1998; **50**(1): 31-6.
175. Schutte CM, van der Meyden CH. A prospective study of Glasgow Coma Scale (GCS), age, CSF-neutrophil count, and CSF-protein and glucose levels as prognostic indicators in 100 adult patients with meningitis. *J Infect* 1998; **37**(2): 112-5.
176. Shembesh NM, el Bargathy SM, Rao BN, Kashbur IM. A prospective study of bacterial meningitis in children from north-eastern Libya. *Ann Trop Paediatr* 1998; **18**(3): 203-7.

177. Campagne G, Schuchat A, Djibo S, Ousseini A, Cisse L, Chippaux JP. Epidemiology of bacterial meningitis in Niamey, Niger, 1981-96. *Bull World Health Organ* 1999; **77**(6): 499-508.
178. Daoud AS, Batieha A, Al-Sheyyab M, Abuekteish F, Obeidat A, Mahafza T. Lack of effectiveness of dexamethasone in neonatal bacterial meningitis. *Eur J Pediatr* 1999; **158**(3): 230-3.
179. Dawson KG, Emerson JC, Burns JL. Fifteen years of experience with bacterial meningitis. *Pediatr Infect Dis J* 1999; **18**(9): 816-22.
180. Moyen G, Mbika-Cardorelle A. [Bacterial meningitis in infants and children at the Brazzaville University Hospital]. *Arch Pediatr* 1999; **6**(1): 108-9.
181. Nathoo KJ, Bannerman CH, Pirie DJ. Pattern of admissions to the paediatric medical wards (1995 to 1996) at Harare Hospital, Zimbabwe. *Cent Afr J Med* 1999; **45**(10): 258-63.
182. Okome-Nkoumou M, Loembe PM. [Bacterial meningitis in the adult. Study of 85 cases observed in the infectious disease unit of the Fondation Jeanne Ebori (F.J.E.), Libreville, Gabon]. *Bull Soc Pathol Exot* 1999; **92**(5): 288-91.
183. Palmer A, Weber M, Bojang K, McKay T, Adegbola R. Acute bacterial meningitis in The Gambia: a four-year review of paediatric hospital admissions. *J Trop Pediatr* 1999; **45**(1): 51-3.
184. Pena JA, Jimenez L. [Prognosis of bacterial meningitis]. *Rev Neurol* 1999; **29**(4): 311-5.
185. Silber E, Sonnenberg P, Ho KC, Koornhof HJ, Eintracht S, Morris L, Saffer D. Meningitis in a community with a high prevalence of tuberculosis and HIV infection. *J Neurol Sci* 1999; **162**(1): 20-6.
186. Struillou L, Ninin E, Berranger C, et al. [Community-acquired bacterial meningitis in the Loire-Atlantic region: evolution of pneumococcal and meningococcal sensitivity to penicillin]. *Presse Med* 1999; **28**(8): 389-94.
187. Tang LM, Chen ST, Hsu WC, Lyu RK. Acute bacterial meningitis in adults: a hospital-based epidemiological study. *QJM* 1999; **92**(12): 719-25.
188. Chotmongkol V, Techoruangwiwat C. Community acquired-bacterial meningitis in adults. *Southeast Asian J Trop Med Public Health* 2000; **31**(3): 506-8.
189. Gordon SB, Walsh AL, Chaponda M, et al. Bacterial meningitis in Malawian adults: pneumococcal disease is common, severe, and seasonal. *Clin Infect Dis* 2000; **31**(1): 53-7.
190. Klinger G, Chin CN, Beyene J, Perlman M. Predicting the outcome of neonatal bacterial meningitis. *Pediatrics* 2000; **106**(3): 477-82.
191. Moller K, Hogh P, Larsen FS, Strauss GI, Skinhoj P, Sperling BK, Knudsen GM. Regional cerebral blood flow during hyperventilation in patients with acute bacterial meningitis. *Clin Physiol* 2000; **20**(5): 399-410.
192. Nel E. Neonatal meningitis: mortality, cerebrospinal fluid, and microbiological findings. *J Trop Pediatr* 2000; **46**(4): 237-9.
193. Ray G, Aneja S, Jain M, Batra S. Evaluation of free radical status in CSF in childhood meningitis. *Ann Trop Paediatr* 2000; **20**(2): 115-20.
194. Zanelli S, Gillet Y, Stamm D, Lina G, Floret D. [Bacterial meningitis in infants 1 to 8 weeks old]. *Arch Pediatr* 2000; **7 Suppl 3**: 565s-71s.
195. Almuneef M, Alshaalan M, Memish Z, Alalola S. Bacterial meningitis in Saudi Arabia: the impact of Haemophilus influenzae type b vaccination. *J Chemother* 2001; **13 Suppl 1**: 34-9.
196. Berkley JA, Mwangi I, Ngetsa CJ, Mwarumba S, Lowe BS, Marsh K, Newton CR. Diagnosis of acute bacterial meningitis in children at a district hospital in sub-Saharan Africa. *Lancet* 2001; **357**(9270): 1753-7.
197. Bonsu BK, Harper MB. Fever interval before diagnosis, prior antibiotic treatment, and clinical outcome for young children with bacterial meningitis. *Clin Infect Dis* 2001; **32**(4): 566-72.
198. Holt DE, Halket S, de Louvois J, Harvey D. Neonatal meningitis in England and Wales: 10 years on. *Arch Dis Child Fetal Neonatal Ed* 2001; **84**(2): F85-9.
199. Johnson WB, Adedoyin OT, Abdulkarim AA, Olanrewaju WI. Bacterial pathogens and outcome determinants of childhood pyogenic meningitis in Ilorin, Nigeria. *Afr J Med Med Sci* 2001; **30**(4): 295-303.
200. Madhi SA, Madhi A, Petersen K, Khoosal M, Klugman KP. Impact of human immunodeficiency virus type 1 infection on the epidemiology and outcome of bacterial meningitis in South African children. *Int J Infect Dis* 2001; **5**(3): 119-25.

201. McMillan DA, Lin CY, Aronin SI, Quagliarello VJ. Community-acquired bacterial meningitis in adults: categorization of causes and timing of death. *Clinical infectious diseases : an official publication of the Infectious Diseases Society of America* 2001; **33**(7): 969-75.
202. Miner JR, Heegaard W, Mapes A, Biros M. Presentation, time to antibiotics, and mortality of patients with bacterial meningitis at an urban county medical center. *J Emerg Med* 2001; **21**(4): 387-92.
203. Neuman HB, Wald ER. Bacterial meningitis in childhood at the Children's Hospital of Pittsburgh: 1988-1998. *Clin Pediatr (Phila)* 2001; **40**(11): 595-600.
204. Sahai S, Mahadevan S, Srinivasan S, Kanungo R. Childhood bacterial meningitis in Pondicherry, South India. *Indian J Pediatr* 2001; **68**(9): 839-41.
205. Weiss DP, Coplan P, Guess H. Epidemiology of bacterial meningitis among children in Brazil, 1997-1998. *Rev Saude Publica* 2001; **35**(3): 249-55.
206. Ahsan T, Shahid M, Mahmood T, et al. Role of dexamethasone in acute bacterial meningitis in adults. *J Pak Med Assoc* 2002; **52**(6): 233-9.
207. Barboza AG, Ioli P, Zamarbide I, Estrago MI, Castineiras F, de Wouters L. [A study of the incidence and a descriptive analysis of adult non-tuberculous primary bacterial meningitis in a population in Argentina]. *Rev Neurol* 2002; **35**(6): 508-12.
208. Beyrer K, Dreesman J, Thielen H, Windorfer A. [Surveillance system for assessing central nervous infections in Lower Saxony 1998-2000]. *Gesundheitswesen* 2002; **64**(6): 336-43.
209. Chan YC, Wilder-Smith A, Ong BK, Kumarasinghe G, Wilder-Smith E. Adult community acquired bacterial meningitis in a Singaporean teaching hospital. A seven-year overview (1993-2000). *Singapore Med J* 2002; **43**(12): 632-6.
210. Chinchankar N, Mane M, Bhave S, et al. Diagnosis and outcome of acute bacterial meningitis in early childhood. *Indian Pediatr* 2002; **39**(10): 914-21.
211. Duke T, Mokela D, Frank D, Michael A, Paulo T, Mgone J, Kurubi J. Management of meningitis in children with oral fluid restriction or intravenous fluid at maintenance volumes: a randomised trial. *Ann Trop Paediatr* 2002; **22**(2): 145-57.
212. Hemalatha R, Bhaskaram P, Balakrishna N, Saraswathi I. Association of tumour necrosis factor alpha & malnutrition with outcome in children with acute bacterial meningitis. *Indian J Med Res* 2002; **115**: 55-8.
213. Lopez Sastre J, Castrillo" GdH. [Neonatal meningitis. Epidemiological study of the Grupo de Hospitales Castrillo]. *An Esp Pediatr* 2002; **56**(6): 556-63.
214. Migliani R, Clouzeau J, Decousser JW, et al. [Non-tubercular bacterial meningitis in children in Antananarivo, Madagascar]. *Arch Pediatr* 2002; **9**(9): 892-7.
215. Molyneux EM, Walsh AL, Forsyth H, et al. Dexamethasone treatment in childhood bacterial meningitis in Malawi: a randomised controlled trial. *Lancet* 2002; **360**(9328): 211-8.
216. Mwangi I, Berkley J, Lowe B, Peshu N, Marsh K, Newton CR. Acute bacterial meningitis in children admitted to a rural Kenyan hospital: increasing antibiotic resistance and outcome. *Pediatr Infect Dis J* 2002; **21**(11): 1042-8.
217. Oostenbrink R, Moons KG, Derksen-Lubsen G, Grobbee DE, Moll HA. Early prediction of neurological sequelae or death after bacterial meningitis. *Acta Paediatr* 2002; **91**(4): 391-8.
218. Al-Mazrou YY, Musa EK, Abdalla MN, Al-Jeffri MH, Al-Hajjar SH, Mohamed OM. Disease burden and case management of bacterial meningitis among children under 5 years of age in Saudi Arabia. *Saudi Med J* 2003; **24**(12): 1300-7.
219. Chang CJ, Chang WN, Huang LT, et al. Neonatal bacterial meningitis in southern Taiwan. *Pediatr Neurol* 2003; **29**(4): 288-94.
220. Flores-Cordero JM, Amaya-Villar R, Rincon-Ferrari MD, Leal-Noval SR, Garnacho-Montero J, Llanos-Rodriguez AC, Murillo-Cabezas F. Acute community-acquired bacterial meningitis in adults admitted to the intensive care unit: clinical manifestations, management and prognostic factors. *Intensive Care Med* 2003; **29**(11): 1967-73.
221. Kirimi E, Tuncer O, Arslan S, et al. Prognostic factors in children with purulent meningitis in Turkey. *Acta Med Okayama* 2003; **57**(1): 39-44.
222. Rabbani MA, Khan AA, Ali SS, Ahmad B, Baig SM, Khan MA, Wasay M. Spectrum of complications and mortality of bacterial meningitis: an experience from a developing country. *J Pak Med Assoc* 2003; **53**(12): 580-3.

223. Celal A, Faruk GM, Salih H, Kemal CM, Serife A, Faruk KO. Characteristics of acute bacterial meningitis in Southeast Turkey. *Indian J Med Sci* 2004; **58**(8): 327-33.
224. Khwannimit B, Chayakul P, Geater A. Acute bacterial meningitis in adults: a 20 year review. *Southeast Asian J Trop Med Public Health* 2004; **35**(4): 886-92.
225. Luca V, Gessner BD, Luca C, et al. Incidence and etiological agents of bacterial meningitis among children <5 years of age in two districts of Romania. *Eur J Clin Microbiol Infect Dis* 2004; **23**(7): 523-8.
226. Ostergaard C, Benfield T, Lundgren JD, Eugen-Olsen J. Soluble urokinase receptor is elevated in cerebrospinal fluid from patients with purulent meningitis and is associated with fatal outcome. *Scand J Infect Dis* 2004; **36**(1): 14-9.
227. Sallam AK. Etiology and presentation of acute bacterial meningitis in children at Al-Thawrah Hospital, Sana'a, Yemen. *J Ayub Med Coll Abbottabad* 2004; **16**(4): 40-3.
228. Singhi SC, Khetarpal R, Baranwal AK, Singhi PD. Intensive care needs of children with acute bacterial meningitis: a developing country perspective. *Ann Trop Paediatr* 2004; **24**(2): 133-40.
229. van de Beek D, de Gans J, Spanjaard L, Weisfelt M, Reitsma JB, Vermeulen M. Clinical features and prognostic factors in adults with bacterial meningitis. *N Engl J Med* 2004; **351**(18): 1849-59.
230. Wiersinga WJ, van Dellen QM, Spanjaard L, van Kan HJ, Groen AL, Wetsteyn JC. High mortality among patients with bacterial meningitis in a rural hospital in Tanzania. *Ann Trop Med Parasitol* 2004; **98**(3): 271-8.
231. Amsalu S, Assefa A. Meningitis in children beyond the neonatal period in Gondar University hospital. *Ethiop Med J* 2005; **43**(3): 175-80.
232. Bekondi C, Bernede C, Passone N, Minssart P, Kamalo C, Mbolidi D, Germani Y. Primary and opportunistic pathogens associated with meningitis in adults in Bangui, Central African Republic, in relation to human immunodeficiency virus serostatus. *Int J Infect Dis* 2006; **10**(5): 387-95.
233. Deeks SL, MacDonald DM, Squires SG, Medaglia A, Tam T. Bacterial meningitis in Canada: hospitalizations (1994-2001). *Can Commun Dis Rep* 2005; **31**(23): 241-7.
234. Farag H, Abdel-Fattah M, Youssri A. Epidemiological, clinical and prognostic profile of acute bacterial meningitis among children in Alexandria, Egypt. *Indian Journal of Medical Microbiology* 2005; **23**(2): 95-101.
235. Hui AC, Ng KC, Tong PY, Mok V, Chow KM, Wu A, Wong LK. Bacterial meningitis in Hong Kong: 10-years' experience. *Clin Neurol Neurosurg* 2005; **107**(5): 366-70.
236. Lucena R, Fonseca N, Nunes L, et al. Intra-hospital lethality among infants with pyogenic meningitis. *Pediatr Neurol* 2005; **32**(3): 180-3.
237. May M, Daley AJ, Donath S, Isaacs D, Australasian Study Group for Neonatal I. Early onset neonatal meningitis in Australia and New Zealand, 1992-2002. *Arch Dis Child Fetal Neonatal Ed* 2005; **90**(4): F324-7.
238. Odetola FO, Bratton SL. Characteristics and immediate outcome of childhood meningitis treated in the pediatric intensive care unit. *Intensive Care Med* 2005; **31**(1): 92-7.
239. Ogunlesi TA, Okeniyi JA, Oyelami OA. Pyogenic meningitis in Ilesa, Nigeria. *Indian Pediatr* 2005; **42**(10): 1019-23.
240. Al Khorasani A, Banajeh S. Bacterial profile and clinical outcome of childhood meningitis in rural Yemen: a 2-year hospital-based study. *J Infect* 2006; **53**(4): 228-34.
241. Bregani ER, Tarsia P, Pujades E, Van Tien T, Arioli M, Ziglioli E. The 2001 meningitis epidemic in south Chad. *Minerva Med* 2006; **97**(2): 161-73.
242. Elsaid MF, Flamerzi AA, Bessisso MS, Elshafie SS. Acute bacterial meningitis in Qatar. *Saudi Med J* 2006; **27**(2): 198-204.
243. Garges HP, Moody MA, Cotten CM, et al. Neonatal meningitis: what is the correlation among cerebrospinal fluid cultures, blood cultures, and cerebrospinal fluid parameters? *Pediatrics* 2006; **117**(4): 1094-100.
244. Mbelesso P, Tatangba-Bakozo A, Fikouma V. [Bacterial meningiditis in adult patients in Central African hospitals]. *Bull Soc Pathol Exot* 2006; **99**(4): 261-3.
245. Molyneux E, Riordan FA, Walsh A. Acute bacterial meningitis in children presenting to the Royal Liverpool Children's Hospital, Liverpool, UK and the Queen Elizabeth Central Hospital in Blantyre, Malawi: a world of difference. *Ann Trop Paediatr* 2006; **26**(1): 29-37.
246. Pizon AF, Bonner MR, Wang HE, Kaplan RM. Ten years of clinical experience with adult meningitis at an urban academic medical center. *J Emerg Med* 2006; **30**(4): 367-70.

247. Shabani IS, Al-Ateeqi W, Abu-Shanab O, El-Sori H, Omar N, Ahmed HF, Al-Musallam M. Childhood meningitis in Kuwait: epidemiology of etiologic agents and the need for pneumococcal disease prevention. *Med Princ Pract* 2006; **15**(6): 431-5.
248. Singhi SC, Bansal A. Serum cortisol levels in children with acute bacterial and aseptic meningitis. *Pediatr Crit Care Med* 2006; **7**(1): 74-8.
249. Afifi S, Wasfy MO, Azab MA, et al. Laboratory-based surveillance of patients with bacterial meningitis in Egypt (1998-2004). *Eur J Clin Microbiol Infect Dis* 2007; **26**(5): 331-40.
250. Boisier P, Mainassara HB, Sidikou F, Djibo S, Kairo KK, Chanteau S. Case-fatality ratio of bacterial meningitis in the African meningitis belt: we can do better. *Vaccine* 2007; **25 Suppl 1**: A24-9.
251. Dauchy FA, Gruson D, Chene G, et al. Prognostic factors in adult community-acquired bacterial meningitis: a 4-year retrospective study. *Eur J Clin Microbiol Infect Dis* 2007; **26**(10): 743-6.
252. Faustini A, Arca M, Fusco D, Perucci CA. Prognostic factors and determinants of fatal outcome due to bacterial meningitis in the Lazio region of Italy, 1996-2000. *Int J Infect Dis* 2007; **11**(2): 137-44.
253. Johnson AW, Adedoyin OT, Abdul-Karim AA, Olanrewaju AW. Childhood pyogenic meningitis: clinical and investigative indicators of etiology and outcome. *J Natl Med Assoc* 2007; **99**(8): 937-47.
254. Krebs VL, Costa GA. Clinical outcome of neonatal bacterial meningitis according to birth weight. *Arq Neuropsiquiatr* 2007; **65**(4B): 1149-53.
255. Lepur D, Barsic B. Community-acquired bacterial meningitis in adults: antibiotic timing in disease course and outcome. *Infection* 2007; **35**(4): 225-31.
256. Nguyen TH, Tran TH, Thwaites G, et al. Dexamethasone in Vietnamese adolescents and adults with bacterial meningitis. *N Engl J Med* 2007; **357**(24): 2431-40.
257. Peltola H, Roine I, Fernandez J, et al. Adjuvant glycerol and/or dexamethasone to improve the outcomes of childhood bacterial meningitis: a prospective, randomized, double-blind, placebo-controlled trial. *Clin Infect Dis* 2007; **45**(10): 1277-86.
258. Scarborough M, Gordon SB, Whitty CJ, et al. Corticosteroids for bacterial meningitis in adults in sub-Saharan Africa. *N Engl J Med* 2007; **357**(24): 2441-50.
259. Theodoridou MN, Vasilopoulou VA, Atsali EE, Pangalis AM, Mostrou GJ, Syriopoulou VP, Hadjichristodoulou CS. Meningitis registry of hospitalized cases in children: epidemiological patterns of acute bacterial meningitis throughout a 32-year period. *BMC Infect Dis* 2007; **7**: 101.
260. Airede KI, Adeyemi O, Ibrahim T. Neonatal bacterial meningitis and dexamethasone adjunctive usage in Nigeria. *Niger J Clin Pract* 2008; **11**(3): 235-45.
261. Bercion R, Bobossi-Serengbe G, Gody JC, Beyam EN, Manirakiza A, Le Faou A. Acute bacterial meningitis at the 'Complexe Pediatrique' of Bangui, Central African Republic. *J Trop Pediatr* 2008; **54**(2): 125-8.
262. Franco-Paredes C, Lammoglia L, Hernandez I, Santos-Preciado JI. Epidemiology and outcomes of bacterial meningitis in Mexican children: 10-year experience (1993-2003). *Int J Infect Dis* 2008; **12**(4): 380-6.
263. Lagunju IA, Falade AG, Akinbami FO, Adegbola R, Bakare RA. Childhood bacterial meningitis in Ibadan, Nigeria--antibiotic sensitivity pattern of pathogens, prognostic indices and outcome. *Afr J Med Med Sci* 2008; **37**(2): 185-91.
264. Lazzarini L, Toti M, Fabris P, et al. Clinical features of bacterial meningitis in Italy: a multicenter prospective observational study. *J Chemother* 2008; **20**(4): 478-87.
265. Mongelluzzo J, Mohamad Z, Ten Have TR, Shah SS. Corticosteroids and mortality in children with bacterial meningitis. *JAMA* 2008; **299**(17): 2048-55.
266. Pelkonen T, Roine I, Monteiro L, et al. Acute childhood bacterial meningitis in Luanda, Angola. *Scand J Infect Dis* 2008; **40**(11-12): 859-66.
267. Sigauque B, Roca A, Sanz S, et al. Acute bacterial meningitis among children, in Manhica, a rural area in Southern Mozambique. *Acta Trop* 2008; **105**(1): 21-7.
268. Cabellos C, Verdaguer R, Olmo M, et al. Community-acquired bacterial meningitis in elderly patients: experience over 30 years. *Medicine (Baltimore)* 2009; **88**(2): 115-9.
269. Dzupova O, Rozsypal H, Prochazka B, Benes J. Acute bacterial meningitis in adults: predictors of outcome. *Scand J Infect Dis* 2009; **41**(5): 348-54.

270. Gurley ES, Hossain MJ, Montgomery SP, et al. Etiologies of bacterial meningitis in Bangladesh: results from a hospital-based study. *Am J Trop Med Hyg* 2009; **81**(3): 475-83.
271. Ishihara M, Kamei S, Taira N, et al. Hospital-based study of the prognostic factors in adult patients with acute community-acquired bacterial meningitis in Tokyo, Japan. *Intern Med* 2009; **48**(5): 295-300.
272. Roca A, Bassat Q, Morais L, et al. Surveillance of acute bacterial meningitis among children admitted to a district hospital in rural Mozambique. *Clin Infect Dis* 2009; **48 Suppl 2**: S172-80.
273. Tiskumara R, Fakharee SH, Liu CQ, et al. Neonatal infections in Asia. *Arch Dis Child Fetal Neonatal Ed* 2009; **94**(2): F144-8.
274. Traore Y, Tameklo TA, Njanpop-Lafourcade BM, et al. Incidence, seasonality, age distribution, and mortality of pneumococcal meningitis in Burkina Faso and Togo. *Clin Infect Dis* 2009; **48 Suppl 2**: S181-9.
275. Abdulrab A, Algobaty F, Salem AK, Mohammed YA. Acute bacterial meningitis in adults: a hospital based study in Yemen. *Jpn J Infect Dis* 2010; **63**(2): 128-31.
276. Aletayeb MH, Ahmad FS, Masood D. Eleven-year study of causes of neonatal bacterial meningitis in Ahvaz, Iran. *Pediatr Int* 2010; **52**(3): 463-6.
277. Ba O, Fleming JA, Dieye Y, et al. Hospital surveillance of childhood bacterial meningitis in Senegal and the introduction of Haemophilus influenzae type b conjugate vaccine. *Am J Trop Med Hyg* 2010; **83**(6): 1330-5.
278. Bentlin MR, Ferreira GL, Rugolo LM, Silva GH, Mondelli AL, Rugolo Junior A. Neonatal meningitis according to the microbiological diagnosis: a decade of experience in a tertiary center. *Arq Neuropsiquiatr* 2010; **68**(6): 882-7.
279. Cho HK, Lee H, Kang JH, et al. The causative organisms of bacterial meningitis in Korean children in 1996-2005. *J Korean Med Sci* 2010; **25**(6): 895-9.
280. Erdem H, Kilic S, Coskun O, et al. Community-acquired acute bacterial meningitis in the elderly in Turkey. *Clin Microbiol Infect* 2010; **16**(8): 1223-9.
281. Hudeckova H, Jesenak M, Maria A, Svihrova V, Banovcin P. National analysis of bacterial meningitis in Slovakia, 1997-2007. *Public Health Rep* 2010; **125**(1): 129-36.
282. Mankhambo LA, Banda DL, Group IPDS, et al. The role of angiogenic factors in predicting clinical outcome in severe bacterial infection in Malawian children. *Crit Care* 2010; **14**(3): R91.
283. Moon SY, Chung DR, Kim SW, et al. Changing etiology of community-acquired bacterial meningitis in adults: a nationwide multicenter study in Korea. *Eur J Clin Microbiol Infect Dis* 2010; **29**(7): 793-800.
284. Perez AE, Dickinson FO, Rodriguez M. Community acquired bacterial meningitis in Cuba: a follow up of a decade. *BMC Infect Dis* 2010; **10**: 130.
285. Su CM, Chang WN, Tsai NW, Huang CR, Wang HC, Lu CH. Clinical features and outcome of community-acquired bacterial meningitis in adult patients with liver cirrhosis. *Am J Med Sci* 2010; **340**(6): 452-6.
286. Talbert AW, Mwaniki M, Mwarumba S, Newton CR, Berkley JA. Invasive bacterial infections in neonates and young infants born outside hospital admitted to a rural hospital in Kenya. *Pediatr Infect Dis J* 2010; **29**(10): 945-9.
287. Vibha D, Bhatia R, Prasad K, Srivastava MV, Tripathi M, Singh MB. Clinical features and independent prognostic factors for acute bacterial meningitis in adults. *Neurocrit Care* 2010; **13**(2): 199-204.
288. Ajdukiewicz KM, Cartwright KE, Scarborough M, et al. Glycerol adjuvant therapy in adults with bacterial meningitis in a high HIV seroprevalence setting in Malawi: a double-blind, randomised controlled trial. *Lancet Infect Dis* 2011; **11**(4): 293-300.
289. Pelkonen T, Roine I, Cruzeiro ML, Pitkaranta A, Kataja M, Peltola H. Slow initial beta-lactam infusion and oral paracetamol to treat childhood bacterial meningitis: a randomised, controlled trial. *Lancet Infect Dis* 2011; **11**(8): 613-21.
290. Thigpen MC, Whitney CG, Messonnier NE, et al. Bacterial meningitis in the United States, 1998-2007. *N Engl J Med* 2011; **364**(21): 2016-25.
291. Vashishtha VM, Garg A, John TJ. Etiology of acute bacterial meningitis in hospitalized children in western Uttar Pradesh. *Indian Pediatr* 2011; **48**(12): 985-6.
292. Organization" WH. Meningitis in Burkina Faso, Chad, Niger, Nigeria and Ghana: 2010 epidemic season. *Wkly Epidemiol Rec* 2011; **86**(15): 143-51.
293. Fonseca de Souza S, Costa Mda C, Paim JS, Natividade MS, Pereira SM, Andrade AM, Teixeira MG. Bacterial meningitis and living conditions. *Rev Soc Bras Med Trop* 2012; **45**(3): 323-8.

294. Juganariu G, Miftode E, Teodor D, Leca D, Dorobat CM. Clinical features and course of bacterial meningitis in children. *Rev Med Chir Soc Med Nat Iasi* 2012; **116**(3): 722-6.
295. Kra O, Ouattara B, Aba T, Kadjane NJ, Kadjo K, Bissagnene E, Kadio A. [Morbidity and mortality from infectious diseases at the Military Hospital of Abidjan, Cote d'Ivoire]. *Med Sante Trop* 2012; **22**(1): 75-8.
296. Namani S, Milenkovic Z, Kuchar E, Koci R, Mehmeti M. Mortality from bacterial meningitis in children in Kosovo. *J Child Neurol* 2012; **27**(1): 46-50.
297. Nansera D, Max I, Annet K, Gessner BD. Bacterial meningitis among children under the age of 2 years in a high human immunodeficiency virus prevalence area after Haemophilus influenzae type b vaccine introduction. *J Paediatr Child Health* 2012; **48**(4): 324-8.
298. Tarvij Eslami S, Nassirian H, Mojgan BM, Bahieh ZZ, Elham H, Alimohamad N, Ehsan S. Comparison of cerebrospinal fluid in newborns and in infants </= 2 months old with or without meningitis. *Pediatr Int* 2012; **54**(3): 336-40.
299. Vazquez JA, Adducci Mdel C, Coll C, Godoy Monzon D, Iserson KV. Acute meningitis prognosis using cerebrospinal fluid interleukin-6 levels. *J Emerg Med* 2012; **43**(2): 322-7.
300. Ben Hamouda H, Ben Haj Khalifa A, Hamza MA, Ayadi A, Soua H, Khedher M, Sfar MT. [Clinical outcome and prognosis of neonatal bacterial meningitis]. *Arch Pediatr* 2013; **20**(9): 938-44.
301. Butsashvili M, Kandelaki G, Eloshvili M, Chlikadze R, Imnadze P, Avaliani N. Surveillance of bacterial meningitis in the country of Georgia, 2006-2010. *J Community Health* 2013; **38**(4): 724-6.
302. Ergaz Z, Benenson S, Cohen MJ, Braunstein R, Bar-Oz B. No change in antibiotic susceptibility patterns in the neonatal ICU over two decades. *Pediatr Crit Care Med* 2013; **14**(2): 164-70.
303. Kavuncuoglu S, Gursoy S, Turel O, Aldemir EY, Hosaf E. Neonatal bacterial meningitis in Turkey: epidemiology, risk factors, and prognosis. *J Infect Dev Ctries* 2013; **7**(2): 73-81.
304. Khowaja AR, Mohiuddin S, Cohen AL, et al. Mortality and neurodevelopmental outcomes of acute bacterial meningitis in children aged <5 years in Pakistan. *J Pediatr* 2013; **163**(1 Suppl): S86-S91 e1.
305. Mahmoudi S, Zandi H, Pourakbari B, Ashtiani MT, Mamishi S. Acute bacterial meningitis among children admitted into an Iranian referral children's hospital. *Jpn J Infect Dis* 2013; **66**(6): 503-6.
306. Porobic-Jahic H, Piljic D, Jahic R, Ahmetagic S, Numanovic F. Etiology of bacterial meningitis in children in Tuzla Canton. *Med Arch* 2013; **67**(1): 13-6.
307. Scott S, Altanseseg D, Sodbayer D, et al. Impact of Haemophilus influenzae Type b conjugate vaccine in Mongolia: prospective population-based surveillance, 2002-2010. *J Pediatr* 2013; **163**(1 Suppl): S8-S11.
308. Snaebjarnardottir K, Erlendsdottir H, Reynisson IK, et al. Bacterial meningitis in children in Iceland, 1975-2010: a nationwide epidemiological study. *Scand J Infect Dis* 2013; **45**(11): 819-24.
309. Teleb N, Pilishvili T, Van Beneden C, et al. Bacterial meningitis surveillance in the Eastern Mediterranean region, 2005-2010: successes and challenges of a regional network. *J Pediatr* 2013; **163**(1 Suppl): S25-31.
310. Banajeh SM, Ashoor O, Al-Magramy AS. Childhood very severe pneumonia and meningitis-related hospitalization and death in Yemen, before and after introduction of H. influenzae type b (Hib) vaccine. *East Mediterr Health J* 2014; **20**(7): 431-41.
311. Bodilsen J, Dalager-Pedersen M, Schonheyder HC, Nielsen H. Dexamethasone treatment and prognostic factors in community-acquired bacterial meningitis: a Danish retrospective population-based cohort study. *Scand J Infect Dis* 2014; **46**(6): 418-25.
312. Levy C, Varon E, Taha MK, Bechet S, Bonacorsi S, Cohen R, Bingen E. [Changes in bacterial meningitis in French children resulting from vaccination]. *Arch Pediatr* 2014; **21**(7): 736-44.
313. Molyneux EM, Kawaza K, Phiri A, et al. Glycerol and acetaminophen as adjuvant therapy did not affect the outcome of bacterial meningitis in Malawian children. *Pediatr Infect Dis J* 2014; **33**(2): 214-6.
314. Namani SA, Koci RA, Qehaja-Bucaj E, Ajazaj-Berisha L, Mehmeti M. The epidemiology of bacterial meningitis in Kosovo. *J Infect Dev Ctries* 2014; **8**(7): 823-30.
315. Okike IO, Johnson AP, Henderson KL, et al. Incidence, etiology, and outcome of bacterial meningitis in infants aged <90 days in the United kingdom and Republic of Ireland: prospective, enhanced, national population-based surveillance. *Clin Infect Dis* 2014; **59**(10): e150-7.

316. Thornorethardottir A, Erlendsdottir H, Sigurethardottir B, Harethardottir H, Reynisson IK, Gottfreethsson M, Guethmundsson S. Bacterial meningitis in adults in Iceland, 1995-2010. *Scand J Infect Dis* 2014; **46**(5): 354-60.
317. Correa-Lima AR, de Barros Miranda-Filho D, Valenca MM, Andrade-Valenca L. Risk Factors for Acute Symptomatic Seizure in Bacterial Meningitis in Children. *J Child Neurol* 2015; **30**(9): 1182-5.
318. Hu R, Gong Y, Wang Y. Relationship of Serum Procalcitonin Levels to Severity and Prognosis in Pediatric Bacterial Meningitis. *Clin Pediatr (Phila)* 2015; **54**(12): 1141-4.
319. Kamoun F, Dowlut MB, Ameur SB, et al. Neonatal purulent meningitis in southern Tunisia: Epidemiology, bacteriology, risk factors and prognosis. *Fetal Pediatr Pathol* 2015; **34**(4): 233-40.
320. Lin MC, Chiu NC, Chi H, Ho CS, Huang FY. Evolving trends of neonatal and childhood bacterial meningitis in northern Taiwan. *J Microbiol Immunol Infect* 2015; **48**(3): 296-301.
321. Mora Mora LA, Arco Espinosa ME, Plumet J, Micheli F. [Community acquired bacterial meningitis in patients over 60]. *Medicina (B Aires)* 2015; **75**(6): 367-72.
322. Olson D, Lamb MM, Gaensbauer JT, Todd JK, Halsey NA, Asturias EJ, Guatemala Pediatric Bacterial Surveillance Working G. Risk Factors for Death and Major Morbidity in Guatemalan Children with Acute Bacterial Meningitis. *Pediatr Infect Dis J* 2015; **34**(7): 724-8.
323. Shrestha RG, Tandukar S, Ansari S, et al. Bacterial meningitis in children under 15 years of age in Nepal. *BMC Pediatr* 2015; **15**: 94.
324. Softic I, Tahirovic H, Hasanhodzic M. Neonatal bacterial meningitis: Results from a cross-sectional hospital based study. *Acta Med Acad* 2015; **44**(2): 117-23.
325. Tan J, Kan J, Qiu G, Zhao D, Ren F, Luo Z, Zhang Y. Clinical Prognosis in Neonatal Bacterial Meningitis: The Role of Cerebrospinal Fluid Protein. *PLoS One* 2015; **10**(10): e0141620.
326. Bari A, Zeeshan F, Zafar A, Ejaz H, Iftikhar A, Rathore AW. Childhood Acute Bacterial Meningitis: Clinical Spectrum, Bacteriological Profile and Outcome. *J Coll Physicians Surg Pak* 2016; **26**(10): 822-6.
327. Baunbaek-Knudsen G, Solling M, Farre A, Benfield T, Brandt CT. Improved outcome of bacterial meningitis associated with use of corticosteroid treatment. *Infect Dis (Lond)* 2016; **48**(4): 281-6.
328. Coldiron ME, Salou H, Sidikou F, et al. Case-Fatality Rates and Sequelae Resulting from Neisseria meningitidis Serogroup C Epidemic, Niger, 2015. *Emerg Infect Dis* 2016; **22**(10): 1827-9.
329. Glimaker M, Brink M, Naucler P, Sjolin J. Betamethasone and dexamethasone in adult community-acquired bacterial meningitis: a quality registry study from 1995 to 2014. *Clin Microbiol Infect* 2016; **22**(9): 814 e1- e7.
330. Kambire D, Soeters HM, Ouedraogo-Traore R, et al. Nationwide Trends in Bacterial Meningitis before the Introduction of 13-Valent Pneumococcal Conjugate Vaccine-Burkina Faso, 2011-2013. *PLoS One* 2016; **11**(11): e0166384.
331. Wee LY, Tanugroho RR, Thoon KC, et al. A 15-year retrospective analysis of prognostic factors in childhood bacterial meningitis. *Acta Paediatr* 2016; **105**(1): e22-9.
332. Gudina EK, Tesfaye M, Adane A, et al. Adjunctive dexamethasone therapy in unconfirmed bacterial meningitis in resource limited settings: is it a risk worth taking? *BMC Neurol* 2016; **16**(1): 153.
333. Hasbun R, Rosenthal N, Balada-Llasat JM, et al. Epidemiology of Meningitis and Encephalitis in the United States, 2011-2014. *Clin Infect Dis* 2017; **65**(3): 359-63.
334. Kaburi BB, Kubio C, Kenu E, Nyarko KM, Mahama JY, Sackey SO, Afari EA. Evaluation of the enhanced meningitis surveillance system, Yendi municipality, northern Ghana, 2010-2015. *BMC Infect Dis* 2017; **17**(1): 306.
335. Kafle DR, Subedi M, Thapa M. Outcome of Patients with Meningitis and Encephalitis at Tertiary Care Hospital in Eastern Nepal. *Kathmandu Univ Med J (KUMJ)* 2017; **15**(57): 40-4.
336. Lien CY, Huang CR, Tsai WC, et al. Epidemiologic trend of adult bacterial meningitis in southern Taiwan (2006-2015). *J Clin Neurosci* 2017; **42**: 59-65.
337. Ouchenir L, Renaud C, Khan S, et al. The Epidemiology, Management, and Outcomes of Bacterial Meningitis in Infants. *Pediatrics* 2017; **140**(1).
338. Park BS, Kim SE, Park SH, et al. Procalcitonin as a potential predicting factor for prognosis in bacterial meningitis. *J Clin Neurosci* 2017; **36**: 129-33.
339. Polkowska A, Toropainen M, Ollgren J, Lyytikainen O, Nuorti JP. Bacterial meningitis in Finland, 1995-2014: a population-based observational study. *BMJ Open* 2017; **7**(5): e015080.

340. Sadeq H, Husain EH, Alkoot A, et al. Childhood meningitis in Kuwait in the era of post pneumococcal conjugate vaccination: A multicenter study. *J Infect Public Health* 2017; **10**(6): 766-9.
341. Wall EC, Mukaka M, Denis B, et al. Goal directed therapy for suspected acute bacterial meningitis in adults and adolescents in sub-Saharan Africa. *PLoS One* 2017; **12**(10): e0186687.
342. Amare AT, Kebede ZT, Welch HD. Epidemiology of bacterial meningitis in children admitted to Gondar University Hospital in the post pneumococcal vaccine era. *Pan Afr Med J* 2018; **31**: 193.
343. Jumanne S, Meda J, Hokororo A, Leshabari K. Clinical Predictors of Malaria, Acute Bacterial Meningitis and Treatment Outcomes among Febrile Children Admitted with Altered Mental Status in Northwestern Tanzania. *J Trop Pediatr* 2018; **64**(5): 426-33.
344. Kumar M, Tripathi S, Kumar H, Singh SN. Predictors of Poor Outcome in Neonates with Pyogenic Meningitis in a Level-Three Neonatal Intensive Care Unit of Developing Country. *J Trop Pediatr* 2018; **64**(4): 297-303.
345. Brown BL, Fidell A, Ingolia G, Murad E, Beckham JD. Infectious causes and outcomes in patients presenting with cerebral spinal fluid pleocytosis. *J Neurovirol* 2019; **25**(4): 448-56.
346. De Almeida SM, Barros NC, Petterle R, Nogueira K. Comparison of cerebrospinal fluid lactate with physical, cytological, and other biochemical characteristics as prognostic factors in acute bacterial meningitis. *Arq Neuropsiquiatr* 2019; **77**(12): 871-80.
347. El-Naggar W, Afifi J, McMillan D, Toye J, Ting J, Yoon EW, Shah PS. Epidemiology of Meningitis in Canadian Neonatal Intensive Care Units. *Pediatr Infect Dis J* 2019; **38**(5): 476-80.
348. Fuentes-Antras J, Ramirez-Torres M, Osorio-Martinez E, Lorente M, Lorenzo-Almoros A, Lorenzo O, Gorgolas M. Acute Community-Acquired Bacterial Meningitis: Update on Clinical Presentation and Prognostic factors. *New Microbiol* 2019; **41**(4): 81-7.
349. Haydar SM, Hallit SR, Hallit RR, Salameh PR, Faddoul LJ, Chahine BA, Malaeb DN. Adherence to international guidelines for the treatment of meningitis infections in Lebanon. *Saudi Med J* 2019; **40**(3): 260-5.
350. Larsen F, Brandt CT, Larsen L, et al. Risk factors and prognosis of seizures in adults with community-acquired bacterial meningitis in Denmark: observational cohort studies. *BMJ Open* 2019; **9**(7): e030263.
351. Mwenda JM, Soda E, Weldegebriel G, et al. Pediatric Bacterial Meningitis Surveillance in the World Health Organization African Region Using the Invasive Bacterial Vaccine-Preventable Disease Surveillance Network, 2011-2016. *Clin Infect Dis* 2019; **69**(Suppl 2): S49-S57.
352. Pruitt CM, Neuman MI, Shah SS, et al. Factors Associated with Adverse Outcomes among Febrile Young Infants with Invasive Bacterial Infections. *J Pediatr* 2019; **204**: 177-82 e1.
353. Sonko MA, Dube FS, Okoi CB, et al. Changes in the Molecular Epidemiology of Pediatric Bacterial Meningitis in Senegal After Pneumococcal Conjugate Vaccine Introduction. *Clin Infect Dis* 2019; **69**(Suppl 2): S156-S63.
354. Tagbo BN, Bancroft RE, Fajolu I, et al. Pediatric Bacterial Meningitis Surveillance in Nigeria From 2010 to 2016, Prior to and During the Phased Introduction of the 10-Valent Pneumococcal Conjugate Vaccine. *Clin Infect Dis* 2019; **69**(Suppl 2): S81-S8.
355. Tsolenyanu E, Bancroft RE, Sesay AK, et al. Etiology of Pediatric Bacterial Meningitis Pre- and Post-PCV13 Introduction Among Children Under 5 Years Old in Lome, Togo. *Clin Infect Dis* 2019; **69**(Suppl 2): S97-S104.
356. Huang YH, Yan JH, Kuo KC, Wu WT, Su CM, Chiu IM. Early antibiotics use in young infants with invasive bacterial infection visiting emergency department, a single medical center's experience. *Pediatr Neonatol* 2020; **61**(2): 155-9.
357. Johansson Kostenniemi U, Karlsson L, Silfverdal SA, Mehle C. MeningiSSS: A New Predictive Score to Support Decision on Invasive Procedures to Monitor or Manage the Intracerebral Pressure in Children with Bacterial Meningitis. *Neurocrit Care* 2020; **32**(2): 586-95.
358. Liu G, He S, Zhu X, Li Z. Early onset neonatal bacterial meningitis in term infants: the clinical features, perinatal conditions, and in-hospital outcomes: A single center retrospective analysis. *Medicine (Baltimore)* 2020; **99**(42): e22748.
359. Loutfi A, M ELH, Jayche S, et al. Epidemiological, Cytochemical and Bacteriological Profile of Meningitis among Adults and Children in North West of Morocco. *Pak J Biol Sci* 2020; **23**(7): 891-7.

360. Matulyte E, Kiveryte S, Paulauskiene R, Liukpetryte E, Vaikutyte R, Matulionyte R. Retrospective analysis of the etiology, clinical characteristics and outcomes of community-acquired bacterial meningitis in the University Infectious Diseases Centre in Lithuania. *BMC Infect Dis* 2020; **20**(1): 733.
361. Peros T, van Schuppen J, Bohte A, Hodiamont C, Aronica E, de Haan T. Neonatal bacterial meningitis versus ventriculitis: a cohort-based overview of clinical characteristics, microbiology and imaging. *Eur J Pediatr* 2020; **179**(12): 1969-77.
362. Pomar V, de Benito N, Mauri A, Coll P, Gurgui M, Domingo P. Characteristics and outcome of spontaneous bacterial meningitis in patients with diabetes mellitus. *BMC Infect Dis* 2020; **20**(1): 292.
363. Tubiana S, Varon E, Biron C, et al. Community-acquired bacterial meningitis in adults: in-hospital prognosis, long-term disability and determinants of outcome in a multicentre prospective cohort. *Clin Microbiol Infect* 2020; **26**(9): 1192-200.
364. Adil SM, Hodges SE, Charalambous LT, et al. Paediatric bacterial meningitis in the USA: outcomes and healthcare resource utilization of nosocomial versus community-acquired infection. *J Med Microbiol* 2021; **70**(1).
365. Aimbudlop K, Bruminhent J, Kiertiburanakul S. Infectious causes of acute meningitis among Thai adults in a university hospital. *J Infect Chemother* 2021; **27**(2): 198-204.
366. Bumburidi Y, Utepbergenova G, Yerezhepov B, et al. Etiology of acute meningitis and encephalitis from hospital-based surveillance in South Kazakhstan oblast, February 2017-January 2018. *PLoS One* 2021; **16**(5): e0251494.
367. Kumar D, Pannu AK, Dhibar DP, Singh R, Kumari S. The epidemiology and clinical spectrum of infections of the central nervous system in adults in north India. *Trop Doct* 2021; **51**(1): 48-57.
368. Pelkonen T, Urtti S, Cardoso O, Kyaw MH, Roine I, Peltola H. Risk factors for death in suspected severe bacterial infection in infants aged <90 days in Luanda, Angola. *Int J Infect Dis* 2021; **106**: 223-7.
369. Salmanov AG, Ishchak OM, Dobarin Scapital A C, Susidko OM, Mosendz OV, Korniyenko SM, Voloshyn OA. Perinatal Infections in Ukraine: Results of a Multicenter Study. *Wiad Lek* 2021; **74**(9 cz 1): 2025-32.
370. Savonius O, Rugemalira E, Roine I, Cruzeiro ML, Peltola H, Pelkonen T. Extended Continuous beta-Lactam Infusion With Oral Acetaminophen in Childhood Bacterial Meningitis: A Randomized, Double-blind Clinical Trial. *Clin Infect Dis* 2021; **72**(10): 1738-44.
371. Sunwoo JS, Shin HR, Lee HS, et al. A hospital-based study on etiology and prognosis of bacterial meningitis in adults. *Sci Rep* 2021; **11**(1): 6028.
372. Wong CH, Duque JR, Wong JSC, et al. Epidemiology and Trends of Infective Meningitis in Neonates and Infants Less than 3 Months Old in Hong Kong. *Int J Infect Dis* 2021; **111**: 288-94.
